# Determinants of substrate specificity in a catalytically diverse family of acyl-ACP thioesterases from plants

**DOI:** 10.1186/s12870-022-04003-y

**Published:** 2023-01-02

**Authors:** Rebecca S. Kalinger, Owen Rowland

**Affiliations:** Department of Biology and Institute of Biochemistry, Carleton University, 1125 Colonel By Drive, Ottawa, Ontario K1S 5B6 Canada

**Keywords:** Fatty acid, Enzyme engineering, Lipid synthesis, Substrate specificity, Structure-function, Acyl-ACP thioesterase, Methylketone

## Abstract

**Background:**

ACYL-LIPID THIOESTERASES (ALTs) are a subclass of plastid-localized, fatty acyl-acyl carrier protein (ACP) thioesterase enzymes from plants. They belong to the single hot dog-fold protein family. ALT enzymes generate medium-chain (C6-C14) and C16 fatty acids, methylketone precursors (β-keto fatty acids), and 3-hydroxy fatty acids when expressed heterologously in *E. coli*. The diverse substrate chain-length and oxidation state preferences of ALTs set them apart from other plant acyl-ACP thioesterases, and ALTs show promise as metabolic engineering tools to produce high-value medium-chain fatty acids and methylketones in bacterial or plant systems. Here, we used a targeted motif-swapping approach to explore connections between ALT protein sequence and substrate specificity. Guided by comparative motif searches and computational modelling, we exchanged regions of amino acid sequence between ALT-type thioesterases from *Arabidopsis thaliana, Medicago truncatula,* and *Zea mays* to create chimeric ALT proteins.

**Results:**

Comparing the activity profiles of chimeric ALTs in *E. coli* to their wild-type counterparts led to the identification of interacting regions within the thioesterase domain that shape substrate specificity and enzyme activity. Notably, the presence of a 31-CQH[G/C]RH-36 motif on the central α-helix was shown to shift chain-length specificity towards 12–14 carbon chains, and to be a core determinant of substrate specificity in ALT-type thioesterases with preference for 12–14 carbon 3-hydroxyacyl- and β-ketoacyl-ACP substrates. For an ALT containing this motif to be functional, an additional 108-KXXA-111 motif and compatible sequence spanning aa77–93 of the surrounding β-sheet must also be present, demonstrating that interactions between residues in these regions of the catalytic domain are critical to thioesterase activity. The behaviour of chimeric enzymes in *E. coli* also indicated that aa77–93 play a significant role in dictating whether an ALT will prefer ≤10-carbon or ≥ 12-carbon acyl chain-lengths, and aa91–96 influence selectivity for substrates of fully or partially reduced oxidation states. Additionally, aa64–67 on the hot dog-fold β-sheet were shown to be important for enabling an ALT to act on 3-hydroxy fatty acyl-ACP substrates.

**Conclusions:**

By revealing connections between thioesterase sequence and substrate specificity, this study is an advancement towards engineering recombinant ALTs with product profiles suited for specific applications.

**Supplementary Information:**

The online version contains supplementary material available at 10.1186/s12870-022-04003-y.

## Introduction

Medium-chain fatty acids (MCFAs), usually defined as being 6 to 14 carbons in length, and their derivatives are industrially valuable chemicals. They have numerous uses as components of aviation and diesel biofuels, plasticizers, detergents, and antimicrobials, among others [1–4]. In plants and prokaryotes, de novo fatty acid synthesis begins when acetyl-CoA is carboxylated to malonyl-CoA. The malonyl group is then linked to acyl carrier protein (ACP) via a transacylation reaction. A sequence of four enzymatic reactions follows: condensation of acetyl-CoA with malonyl-ACP to form β-ketoacyl-ACP, reduction to 3-hydroxyacyl-ACP, dehydration to enoyl-ACP, and finally, a second reduction to a fully reduced fatty acyl-ACP extended by two carbons [5, 6]. This four-reaction cycle repeats, beginning with condensation of the growing acyl-ACP with malonyl-ACP, and the acyl chain is sequentially elongated by two-carbon units [5, 6]. In plastids, the major site of de novo fatty acid synthesis in plants, the acyl chain typically reaches 16–18 carbons in length before an acyl thioesterase enzyme cleaves the thioester bond that links the fatty acyl chain to the phosphopantetheine cofactor carried by the ACP, releasing a free fatty acid [5, 7]. However, certain tissue-specific acyl thioesterases interrupt the fatty acid synthesis cycle earlier, cleaving acyl chains before they reach 16–18 carbons. In this way, acyl thioesterases are responsible for fatty acid chain length diversity in particular plant tissue types. There is significant research interest in using fatty acyl thioesterases as metabolic engineering tools to drive fatty acid production towards desired chain lengths and increase MCFA accumulation in oilseed plants or microbial systems [8].

Some oilseed plants naturally accumulate MCFAs in seed triacylglycerols by the action of seed-expressed medium-chain acyl-ACP thioesterases of the FatB class [9–11]. These plastid-localized thioesterases are capable of cleaving a fatty acyl chain from ACP when it is between 6 and 14 carbons long, thereby releasing MCFAs. FatB-type thioesterases have been the focus of most studies to date where heterologous expression of plant thioesterases has been used to increase MCFA production in plants or microbes [12–19]. However, plants and green microalgae possess a second class of plastid-localized, medium-chain acyl-ACP thioesterase known as ALTs (ACYL LIPID THIOESTERASES) [20]. Both FatB- and ALT-type thioesterases belong to the hot dog-fold protein superfamily, named for their catalytic domain structure consisting of a central alpha-helix partially wrapped in a beta-sheet, although ALTs are comprised of a single hot dog-fold thioesterase domain instead of the double hot dog-fold characteristic of FatBs [20–22].

The ability of ALT-type thioesterases to act on a wider range of substrates also distinguishes them from medium-chain FatB-type thioesterases. In addition to cleaving fully reduced acyl chains to release MCFAs, ALTs can act on 3-hydroxyacyl-ACP and β-ketoacyl-ACP intermediates of fatty acid biosynthesis to produce medium-chain 3-hydroxy and β-keto fatty acids [20, 23–25]. METHYLKETONE SYNTHASE 2 (MKS2), an ALT from wild tomato (*Solanum hirsutum* subsp. *glabratum*), and MKS2 orthologues in other solanaceous plants, produce β-keto fatty acids that are enzymatically converted to defensive methylketones (MKs) by companion decarboxylases [23, 25]. β-keto fatty acids are also readily decarboxylated in vitro by brief exposure to heat under acidic conditions [23]. MKs are widely used in the flavouring and fragrance industries and as blending agents for biofuels, while 3-hydroxy fatty acids have applications as components of lubricants and surfactants [26–32]. To date, medium-chain methylketones and 3-hydroxy fatty acids are largely sourced from fossil hydrocarbons, and engineering a sustainable means of producing them remains a challenge [26].

While medium-chain FatBs are only found to date in species with high medium-chain triglyceride content in their seed oil, most plant species, including green microalgae, possess ALTs [20]. Multiple, paralogous ALT-encoding genes are usually present in vascular plant genomes [20, 24]. Heterologous expression of ALTs from 11 diverse plant species in *E. coli* demonstrated that while certain ALTs share general chain-length and oxidation state preferences (i.e. preference for 6–8 carbon or 12–14 carbon chains, preference for β-keto fatty acyl-ACPs or fully reduced acyl-ACPs), no two ALTs have the exact same product profiles in *E. coli* [20, 24]. Typically, an ALT will act on substrates of varied oxidation states and multiple chain lengths. For instance, an ALT with preference for C14-β-ketoacyl-ACPs may also display substantial activity towards fully reduced C8–16 acyl-ACPs [24]. ALTs are therefore potential sustainable sources of many valuable MCFAs, 3-hydroxy fatty acids, and methylketone precursors with numerous industrial uses [20, 24]. At the same time, however, their broad substrate specificities limit their use as metabolic engineering tools, since an ALT will generate some less desirable products alongside compounds of interest for specific applications. Engineering recombinant ALTs with targeted activity profiles would be a worthwhile endeavour, but a detailed understanding of what dictates their chain length and oxidation state preferences is required for this.

Protein mutagenesis through domain-swapping has been used to shed light on the basis of chain-length specificity in medium-chain FatB-type thioesterases, and drive their substrate preferences toward desired chain lengths. By exchanging combinations of similarly-sized sequence fragments between two medium-chain FatB enzymes with differing chain length preferences from the oilseed plant *Cuphea viscossima*, Jing et al. (2018) determined that the N-terminal hot dog-fold domain of medium-chain specific FatBs is responsible for dictating chain length preference, while the C-terminal domain cleaves the thioester bond connecting the fatty acyl chain to ACP [33]. Ziesack et al. (2018) created a chimeric thioesterase with greatly increased affinity for 8:0 acyl-ACP, and another that exhibited both medium- and long-chain thioesterase activities, by sequentially exchanging sequence fragments between an 8:0-specific and a 14:0-specific FatB-type thioesterase from another *Cuphea* species [34].

According to the ThYme (thioester-active enzyme) database, ALT enzymes belong to the Type 9 subfamily of single hot dog-fold thioesterases, within a phylogenetic clade that includes bacterial enzymes such as the YbgC and YbaW medium-chain acyl-CoA thioesterases, and 4-hydroxybenzoyl-CoA thioesterases [35]. Little research has been done on how protein sequence relates to substrate specificity in this clade, and an iterative domain-swapping approach was an attractive starting point for investigating the determinants of substrate specificity in ALTs. However, since ALTs are relatively small proteins, with a single thioesterase domain of ~ 150 residues, and because paralogous ALTs typically share ~ 60–90% sequence identity, the above-described methods for FatBs are not as suitable for ALTs. Smaller sequence fragments could be exchanged between paralogous ALTs to yield informative results, but this would likely be inefficient given the high degree of sequence identity between them, and the large number of recombinant proteins that would need to be analyzed. Additionally, while separate domains are responsible for chain-length specificity and catalytic action in FatBs, the sequence of a single domain necessarily dictates chain-length specificity, oxidation state specificity, and enzyme activity in ALTs, with any mutations having the potential to influence all three [33]. The promiscuity of ALT enzymes presents another complication. FatB-type thioesterases usually generate only one or two fatty acid chain-lengths as major products, and high-throughput screening methods such as colourimetric assays or overexpression in microbial fatty acid auxotrophs can be used to preliminarily identify mutants with altered substrate specificity or activity [36]. The ability of individual ALTs to accommodate many substrates of various chain-lengths, and especially various oxidation states, means a single screening technique will not capture all mutants with interesting product profiles.

Given these challenges, we opted instead for a more targeted approach. Through comparative motif searches, we identified short motifs shared among ALTs with similar chain-length and oxidation state specificities in *E. coli* – specifically, a six-amino-acid (aa) motif on the central alpha-helix of the hot dog-fold domain and a four-amino-acid motif nearby on the underlying beta-sheet. Computational models of the predicted three-dimensional structure of ALTs suggested that residues in these motifs contribute to substrate binding cavity formation. To investigate their potential role in influencing substrate specificity, the identified regions of sequence were exchanged between suitable pairs of ALT enzymes from the model plant *Arabidopsis thaliana,* the eudicot *Medicago truncatula*, and the monocot *Zea mays* to create chimeric ALTs. We report on how these motifs and other regions of the hot dog-fold domain influence ALT chain-length and oxidation state specificities, as determined by comparing the product profiles of chimeric ALT enzymes in *E. coli* to those of ALTs found in nature.

## Results

### Two short amino acid motifs are consistently found in ALTs that have preference for β-ketoacyl-ACP and 3-hydroxyacyl-ACP substrates

**Fig. 1:**
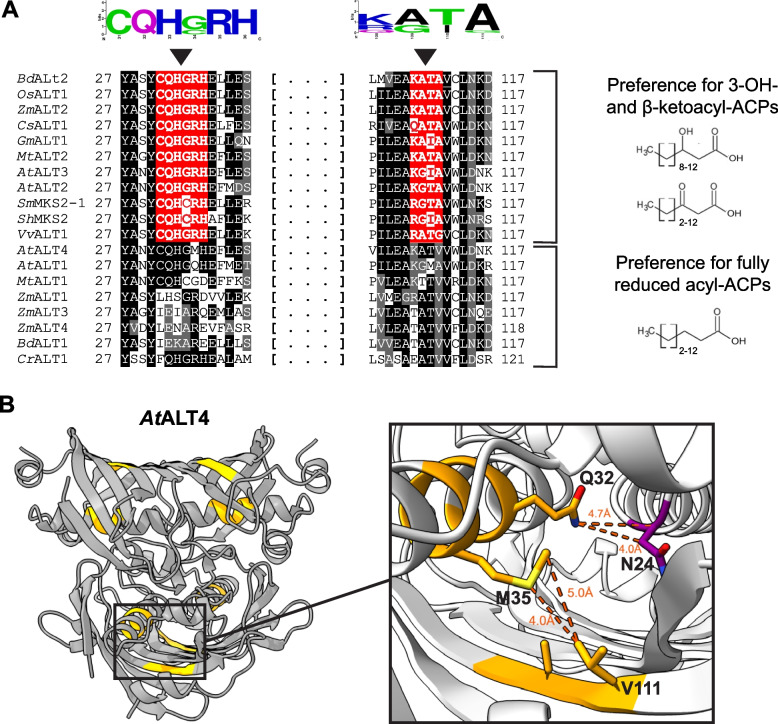
Two amino acid motifs consistently occur in ALTs that prefer β-keto and 3-hydroxy acyl-ACP substrates. **A** Motifs that assort with ALT oxidation state specificity (31-CQH[G/C]RH-36, 108-KXXA-111) are highlighted on a partial alignment of the thioesterase domain sequences of ALT-type thioesterases from diverse plant species, which have been previously characterized in *E. coli* using endogenous acyl-ACP pools [20, 23, 24]. Residues are numbered beginning from the N-terminus of the hot-dog fold thioesterase domain; a predicted N-terminal plastid targeting sequence has been excluded [20, 24]. Protein sequences were aligned using ClustalW and sorted by oxidation state specificity in *E. coli*, and motifs of interest were identified using the STREME program [37, 38]. *At = Arabidopsis thaliana, Bd = Brachypodium distachyon, Cr = Chlamydomonas reinhardtii, Cs = Cannabis sativa, Gm = Glycine max, Mt = Medicago truncatula, Os = Oryza sativa* subsp. *japonica, Sh = Solanum hirsutum* subsp. *glabratum, Sm = Solanum melongena, Vv = Vitis vinifera, Zm = Zea mays*. **B** The structural positions of aa31–36 and aa108–111 are indicated on a three-dimensional model of the predicted *Arabidopsis thaliana* ALT4 homotetramer, generated with AlphaFold 2.0 and HSYMDOCK [39–41]. Examination of this model at the amino acid level shows that the side-chains of certain residues within these motifs are within potential interacting distance of one another (≤5 Å) and a predicted catalytic site residue, N24. Models were visualized in ChimeraX 1.2.5 software [42]

We sought to identify residues that were conserved among ALT-type thioesterases with similar product profiles by aligning the hot dog-fold domain sequences of 19 ALT proteins from diverse plant species, and ordering the aligned sequences according to their known chain-length and oxidation state preferences in *E. coli* [24, 37] (File S1). Comparative motif searches conducted using the STREME program (minimum motif length = 3aa) did not identify any significant features when aligned ALTs were grouped by chain-length specificity (6–10 carbon versus 12–16 carbon chains preferred) [38]. However, when ALT sequences were grouped according to their oxidation state preferences, two motifs located at amino acid residues (aa) 31–36 and 108–111 of the catalytic domain consistently occurred in thioesterases with known preference for β-keto and 3-OH fatty acyl-ACPs over fully reduced fatty acyl-ACPs (Fig. 1A). The first of these motifs reads 31-CQH[G/C]RH-36 (Fig. 1A). In ALT-type thioesterases that primarily produce fully reduced fatty acids in *E. coli*, this motif is altered at one or more residues. These substitutions occur at variable positions, even among ALTs with similar chain-length preferences. For instance, while *A. thaliana* ALT1 and *Z. mays* ALT1 both favour C12-C14 chains, aa31–36 of *At*ALT1 read “CQHG**Q**H”, differing only from the 31-CQH[G/C]RH-36 motif at position 35, while aa31–36 of *Zm*ALT1 read “LHSGRD” (Fig. 1A). The second conserved motif identified reads 108-[K/R/Q][A/G][I/T][A/G]-111. Since the vast majority of ALTs that prefer β-keto and 3-OH acyl-ACP substrates possess Lys at position 108 and Ala at position 111, this motif will henceforward be referred to as 108-KXXA-111. Notably, in ALT proteins with preference for fully reduced acyl-ACPs, the Ala111 or Gly111 residue is usually replaced with Val111 (Fig. 1A, Fig. S1).

The structural positions of the identified motifs were investigated using computational models of the predicted ALT active unit (Fig. 1B, Fig. S1) [39–44]. The predicted tertiary structure of the ALT thioesterase domain generated by AlphaFold 2.0 consists of a 20-residue central alpha-helix surrounded by a 5–6 strand antiparallel beta-sheet, similar to its closest homologue with a known crystal structure, the *E. coli* YbgC thioesterase (PDB ID 5 T06) (Fig. 1B, Fig. S1) [39, 40, 45]*.* As single hot-dog fold thioesterases form active units of homo- or heterodimers, tetramers, or higher-order oligomers in vivo*,* the HSYMDOCK symmetric multimer docking program was used to predict the multimeric structure of ALT enzymes [41]. The most likely homo-oligomeric assembly predicted by HSYMDOCK was a tetramer with dihedral symmetry, again resembling *Ec*YbgC (Fig. 1B) [41, 45]. According to these models, aa31–36 are part of the thioesterase domain’s central α-helix, a core feature of hot dog-fold domain structure. Residues 108–111 are nearby on the surrounding β-sheet, with the side-chain of aa35 in close proximity (≤5 Å) to aa111 (Fig. 1B). Additionally, the side chains of residue 32 and Asn24, the latter of which is conserved in nearly all ALT enzymes, are within 5 Å of one another (Fig. 1B, File S1). When models of the ALT active unit are superposed with that of *Ec*YbgC, Asn24 aligns structurally with a predicted catalytic His25 residue of YbgC [35, 45, 46] (Fig. 1B, Fig. S1). Given that the two motifs found to assort with oxidation state preference in ALTs occupy central positions within the thioesterase domain and are in proximity of one another and key active site residues, we hypothesized that they may be major determinants of substrate specificity in ALT-type thioesterases.

### Disruption of the 31-CQH[G/C]RH-36 motif on the central alpha-helix of the ALT thioesterase domain significantly alters chain-length and oxidation state preference

**Fig. 2:**
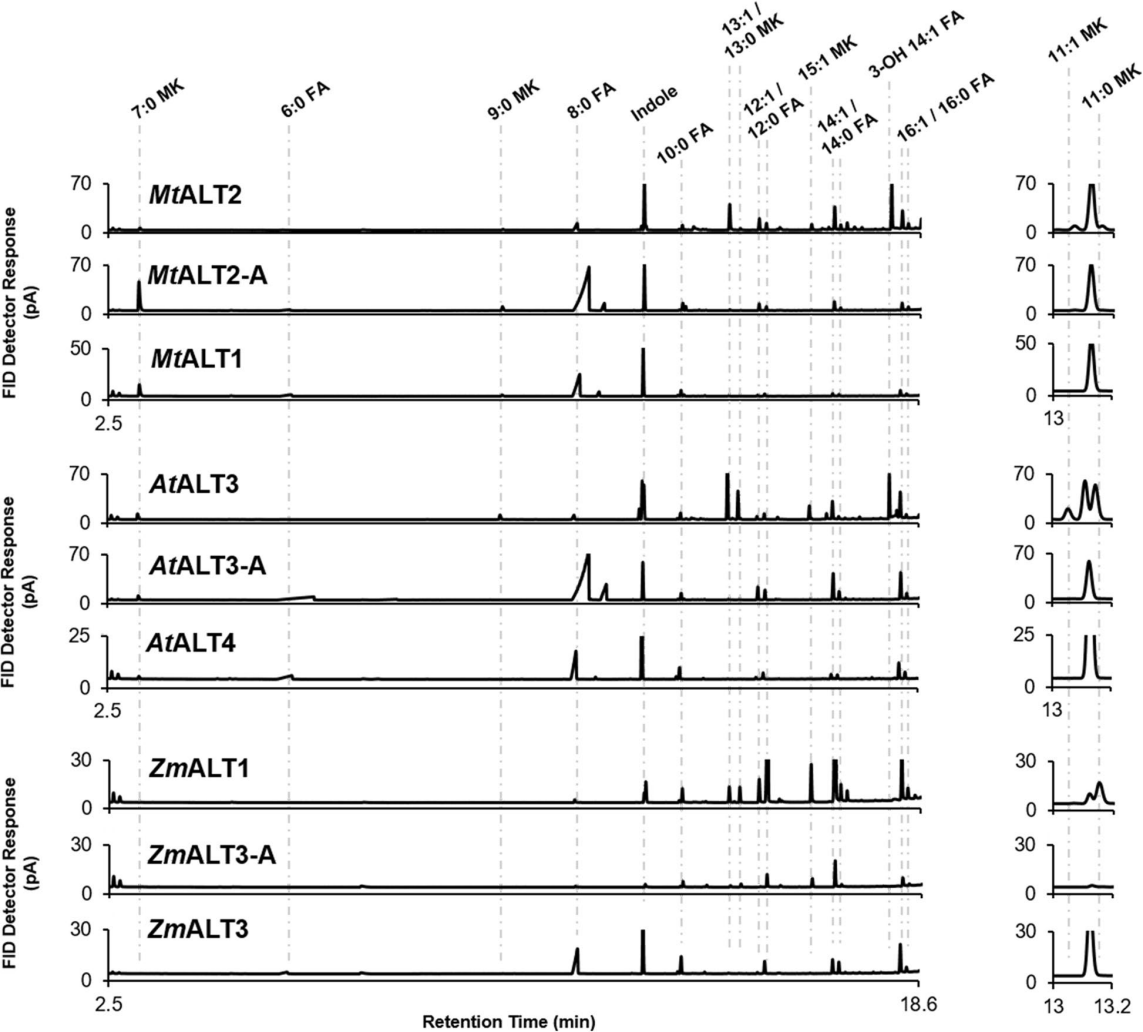
GC-FID chromatograms of secreted lipids from K27(DE3) *E. coli* strains expressing wild-type or mutant ALTs. β-keto fatty acids produced by ALTs were decarboxylated to methylketones with heat and acid treatment prior to identification and quantification by GC-FID and GC-MS analysis. Compounds corresponding to peaks are labelled above the chromatograms (FA = fully reduced fatty acids, MK = methylketone, 3-OH FA = 3-hydroxy fatty acid). In the *Mt*ALT2-A mutant, aa31–36 have been replaced with 31-CQHCGD-36 from *Mt*ALT1, while the rest of the *Mt*ALT2 protein backbone was left unaltered. The *At*ALT3-A mutant contains a single R35M substitution, such that aa31–36 reads 31-CQHGMH-36, like *At*ALT4. In the *Zm*ALT1-A mutant, 31-CQHGRH-36 have been replaced with 31-IEIARQ-36 from *Zm*ALT3, with the rest of the *Zm*ALT1 backbone remaining intact

**Fig. 3:**
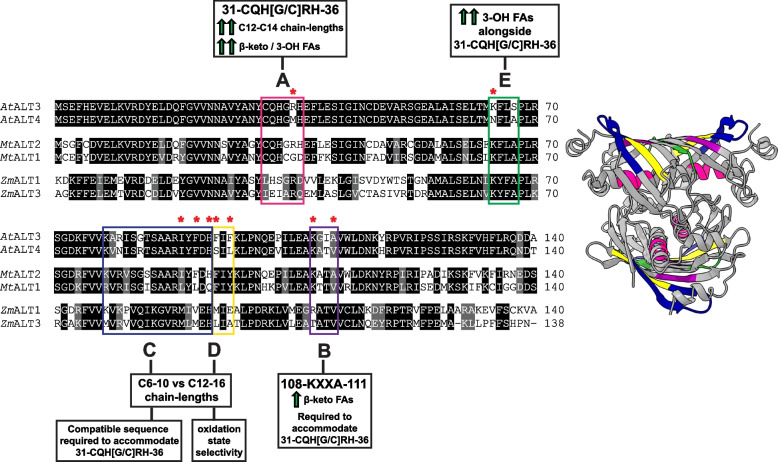
Sequence fragments exchanged between ALT pairs to create chimeric thioesterases and their contributions to substrate specificity. Regions exchanged between ALT enzymes are annotated on pairwise alignments of the hot-dog fold domain sequences of *At*ALT3/4 from *Arabidopsis thaliana, Mt*ALT1/2 from *Medicago truncatula,* and *Zm*ALT1/3 from *Zea mays,* and highlighted on a three-dimensional model of the predicted ALT homotetramer. These are annotated as follows: A = aa31–36, B = aa108–111, C = aa78–93, D = aa94–96, E = aa64–67. A brief summary of how each region influences ALT chain-length and oxidation specificity, as could be deduced from the behaviour of mutant ALT enzymes in *E. coli*, is also provided. Residues predicted to be of particular importance in dictating ALT substrate specificity based on experimental results or computational modelling of protein structure are marked with asterisks. Sequence pairs were aligned using the Needleman-Wünsch algorithm, and structural models of the ALT catalytic domain were generated with AlphaFold 2.0 and HSYMDOCK [39, 40, 47]

A targeted motif-swapping approach was used to investigate whether the motifs identified above influence ALT substrate specificity. The 31-CQH[G/C]RH-36 motif was disrupted in an ALT that normally prefers β-keto and 3-hydroxy acyl-ACP substrates in *E. coli*, and introduced into an ALT that normally shows preference for fully reduced substrates. Pairs of mutant ALT proteins were synthesized where aa31–36 of the hot dog-fold domain were exchanged between one ALT with preference for 14:1 β-ketoacyl- and 3-hydroxyacyl-ACPs, and another ALT from the same species with preference for 8:0 acyl-ACP (Fig. 2, Fig. 3, File S1-S2). The rest of the catalytic domain sequence was unaltered. Specifically, sequence was exchanged between *Mt*ALT2 and *Mt*ALT1 from *Medicago truncatula,* and *At*ALT3 and *At*ALT4 from *Arabidopsis thaliana* to create mutant ALTs. These mutants were annotated with the modifier -A, e.g. “*Mt*ALT2-A”. In *Mt*ALT1, aa31–36 read 31-CQHC**GD**-36. *At*ALT4 and *At*ALT3 only differ within aa31–36 at position 35, and so an *At*ALT3 R35M mutant (*At*ALT3-A) and an *At*ALT4 M35R mutant (*At*ALT4-A) were generated by overlap-extension PCR (Figs. 2 and 3, File S1-S2, Table S1).

The product profiles of the mutant thioesterases were analyzed via expression in K27(DE3) *E. coli* and compared to their wild-type counterparts (Figs. S2-S3, Table S2). The K27(DE3) strain harbours a loss-of-function mutation in the *fadD* acyl-CoA synthetase gene, preventing degradation of fatty acids in the cell by β-oxidation [48]. This results in the accumulation of free fatty acids in the cell, which are subsequently exported into the culture medium. Compounds secreted into the medium were identified and quantified by GC-FID and GC-MS. As the β-keto fatty products of ALTs are too unstable to be detected directly, media samples were treated with heat and acid prior to analysis to chemically convert any β-keto fatty acids present to methylketones [15, 23–25]. This shortens the chain length by one carbon.

**Fig. 4:**
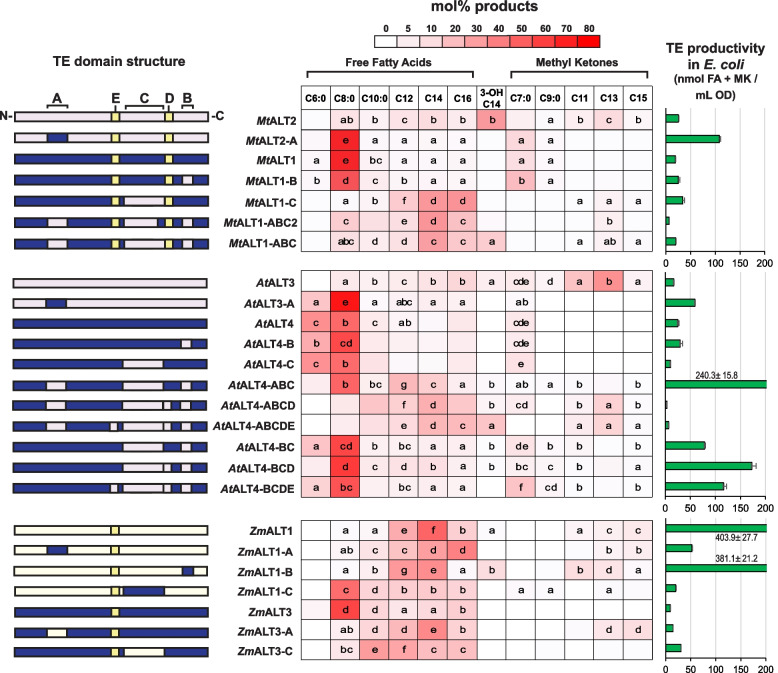
Structural composition, substrate specificity, and productivity of wild-type and mutant ALTs in *E. coli*. The structural composition of chimeric ALT proteins is represented by coloured blocks. Sequence fragments exchanged between ALT pairs to create chimeric enzyme variants are annotated as follows: A = aa31–36, B = aa108–111, C = aa78–93, D = aa94–96, E = aa64–67. Residue positions are numbered beginning from the N-terminus of the hot-dog fold thioesterase domain; a predicted N-terminal plastid targeting sequence has been excluded [20, 24]. Production of individual fatty acid and methylketone species (in mol% of total products), on average, by wild-type and chimeric ALTs in *E. coli* is summarized in three tables, with darker cell shading representing greater proportions (scale shown above tables). Lowercase letters approximate sample means. Mean values in each column sharing a common letter did not differ significantly according to the appropriate statistical test (Tukey’s HSD test or Dunn’s test at the *α* = 0.01 significance level). Spaces that do not contain letters represent mean values that did not differ statistically from zero, or from an *E. coli* strain harbouring an empty pET28a vector according to a right-tailed Student’s *t-*test (*p* < 0.05, *p*-values adjusted using the Holm-Šidak correction). β-keto fatty acids produced by ALTs were decarboxylated to methylketones prior to identification and quantification by GC-MS and GC-FID analysis. The total productivity of ALTs, in units of nmol / mL OD_600_, is represented by green bars. All quantities reported are the average of triplicate samples (*n* = 3), and error reported on these values represents ± SE. ALT variants that did not display detectable thioesterase activity in K27(DE3) *E. coli* were omitted from this figure

Altering the 31-CQH[G/C]RH-36 motif in an ALT with preference for C14 3-OH and β-keto acyl-ACPs led to large-scale changes in oxidation state and chain-length specificity. The *Mt*ALT2-A and *At*ALT3-A mutants adopted similar substrate specificities to *Mt*ALT1 and *At*ALT4, respectively, now showing strong preference for fully reduced substrates and for C8:0 acyl-ACP chains (Figs. 2, 3, and 4, Fig. S2, Table S2). *Mt*ALT1 primarily generates 8:0 FA (71.5 ± 5.8 mol% total product), with 8:0 β-keto FA, the precursor of 7:0 MK, as a secondary product (10.3 ± 1.5 mol%), while the major products of *Mt*ALT2 in *E. coli* are 3-OH 14:1 FA (34.6 ± 2.7 mol%) and the 14:1 β-keto FA precursor of 13:1 MK (11.8 ± 1.1 mol%) (Figs. 2 and 4, Files S2-S4). The *Mt*ALT2-A mutant lost its affinity for > 10 carbon acyl chains, with 8:0 FA and the precursor of 7:0 MK now comprising 75.4 ± 8.3 mol% and 12.8 ± 2.5 mol%, respectively, of its total product yield in *E. coli* (Figs. 2, 3, and 4, Fig. S2, Table S2). The primary products of the *At*ALT3-A mutant were 8:0 FA (69.0 ± 1.8 mol% total product) and 6:0 FA (16.7 ± 0.5 mol% total product), similar to *At*ALT4 (Figs. 2, 3, and 4, Fig. S2, Table S2) [15, 24]. Additionally, cells expressing *At*ALT3-A could no longer generate β-keto or 3-hydroxy fatty acids, aside from small amounts of 8:0 β-keto FA (1.4 ± 0.3 mol% total product), which is a minor product of *At*ALT4 (6.5 ± 1.3 mol% total product). Cultures expressing the *Mt*ALT2-A and *At*ALT3-A variants accumulated nearly 40 times as much 8:0 FA / mL OD_600_ than those expressing wild-type *Mt*ALT2 and *At*ALT3 (Figs. 2 and 4, Table S2).

**Fig. 5:**
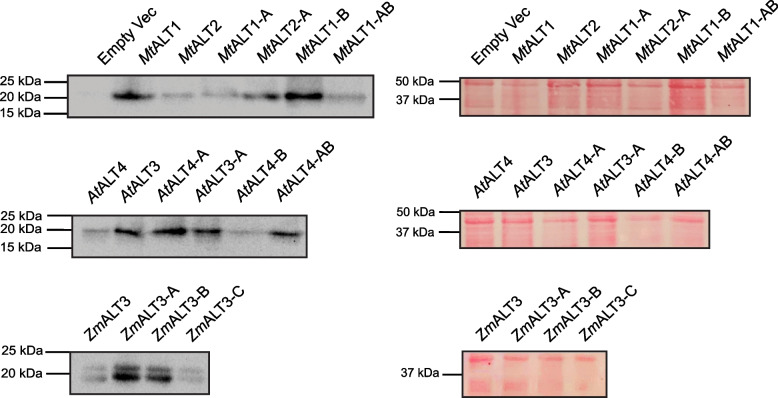
Recombinant ALT-like thioesterases are expressed successfully in K27(DE3) *E. coli*. Left: Immunoblots of crude cell lysates from K27(DE3) *E. coli* strains expressing wild-type or mutant ALT thioesterases show a prominent band at ~ 20 kDa, which is not present in samples from a strain harbouring an empty pET28a vector. 10 *μ*g of total protein was loaded into each lane. Expressed ALTs carried an N-terminal T7-tag, and ALT protein bands were detected by probing the membrane with an anti-T7 mouse monoclonal primary antibody and an anti-mouse horseradish peroxidase-conjugated secondary antibody. All membranes were imaged at 59 s exposure using a BioRad ChemiDoc XRS+ system with ImageLab v6.0.1 software. Images taken from different membranes are separated. Right: Membranes used for immunodetection of ALT proteins stained with Ponceau S following transfer from Tris-Tricine SDS-PAGE gels. All Ponceau-stained membranes were imaged under white light, with an exposure time of 1/30 s. Full-length membranes are displayed in Fig. S4

Conversely, and rather surprisingly, introducing the 31-CQH[G/C]RH-36 motif into *At*ALT4 or *Mt*ALT1 did not give the resulting mutants the ability to generate 12–16 carbon β-keto or 3-OH fatty acids. *E. coli* strains expressing the *At*ALT4-A and *Mt*ALT1-A mutants did not produce significantly increased quantities of any FAs or MK precursors as compared to a strain harbouring an empty pET-28a vector, suggesting that these thioesterases were inactive (Fig. S2, Table S2). Immunoblotting of induced cell lysates ruled out deficiencies in recombinant protein expression as the cause of these observations (Fig. 5, Fig. S4).

Additional *Mt*ALT1 and *At*ALT4 variants were created to assess whether the second motif found to assort with ALT oxidation state specificity, 108-KXXA-111, was required to accommodate the 31-CQH[G/C]RH-36 motif and produce active mutant thioesterases. Residues 108–111 of *Mt*ALT1 and *At*ALT4 were exchanged both alone and in combination with aa31–36 from *Mt*ALT2 or *At*ALT3, respectively (Figs. 3 and 4, Files S1-S2). These residues were shown to influence oxidation state and chain-length specificity by fine-tuning the thioesterase’s affinity for substrates that it is naturally capable of accommodating. When the 108-KXXA-111 motif was introduced alone into *At*ALT4, the resulting *At*ALT4-B mutant generally retained the chain-length preferences of the wild-type enzyme, producing 8:0 FA as its primary product and 6:0 FA as its secondary product (Fig. 4, Fig. S2, Table S2). While 8:0 FA made up a slightly greater proportion (53.8 ± 18.3 mol%) of this variant’s total products as compared to wild-type *At*ALT4, total 6:0 FA accumulation (24.2 ± 4.9 mol%) was slightly decreased (Fig. 4, Table S2). The *Mt*ALT1-B variant displayed significantly increased production of 6:0 FA and 8:0 β-keto fatty acid, with a concomitant decrease in 8:0 FA production. (Fig. 4, Fig. S2, Table S2). The proportion of 6:0 FA generated by this mutant increased by 15.1 mol% compared to wild-type *Mt*ALT1, while that of the 8:0 β-keto FA precursor of 7:0 MK increased by 7.7 mol% (Fig. 4, Files S2- S4). This was balanced by a simultaneous 25.7 mol% decrease in 8:0 FA accumulation. Attempts to shift mutant ALT substrate specificity towards ≥C12 3-hydroxyacyl and β-ketoacyl-ACPs remained unsuccessful, as introduction of the 108-KXXA-111 motif alongside the 31-CQH[G/C]RH-36 motif in either the *At*ALT4 or *Mt*ALT1 backbone still resulted in inactivated mutant enzymes (Fig. S2, Table S2).

### Exchanging residues 31–36 and 108–111 of the thioesterase domain between ALT enzymes that do not possess the 31-CQH[G/C]RH-36 and 108-KXXA-111 motifs also results in changes to chain-length and oxidation state specificity

To further explore the effects of aa31–36 and aa108–111 on ALT substrate specificity, we also exchanged sequence encoding these residues between two ALT enzymes from *Zea mays*, *Zm*ALT1 and *Zm*ALT3, which possess neither the 31-CQH[G/C]RH-36 nor 108-KXXA-111 motifs (Figs. 1, 3, and 4, File S1). *Zm*ALT3 primarily generates 8:0 FA and exclusively acts on fully reduced fatty acyl-ACPs in *E. coli*, while *Zm*ALT1 produces the precursors of C11-C15 MKs (49.5 ± 4.3 nmol / mL OD) and trace amounts of C14 3-OH FAs (2.5 ± 0.4 nmol / mL OD) alongside its major products of 14:1 FA and 12:0 FA (Figs. 2 and 4, Figs. S2-S3, Table S2) [24]*.* When aa31–36 were exchanged between *Zm*ALT1/3, both chimeric mutants, *Zm*ALT1-A and *Zm*ALT3-A, were functional (Figs. 3 and 4, Fig. S2). The *Zm*ALT3-A chimera took on an activity profile that closely resembled *Zm*ALT1, with its chain-length preferences shifted toward C12–14 acyl chains. Its most abundant products in *E. coli* were 14:1 FA (43.2 ± 6.5 mol%) and 12:0 FA (21.3 ± 3.0 mol%) (Figs. 2 and 4, Fig. S2, Table S2). Notably, it also gained the capability to act on β-keto acyl-ACPs like *Zm*ALT1, as C13–15 MK precursors represented 15.9 ± 0.8 mol% of its product output (Fig. 4, Figs. S2-S3, Table S2). On the other hand, replacing aa31–36 in the *Zm*ALT1 backbone with the corresponding sequence from *Zm*ALT3 did not cause the *Zm*ALT1-A chimera to resemble *Zm*ALT3. Its chain-length preferences shifted towards longer, rather than shorter chain-lengths, with 16:1 FA as its major product in *E. coli* (33.8 ± 0.7 mol%) (Fig. 4, Fig. S2, Table S2).

*Zm*ALT1 and *Zm*ALT3 only differ at position 108 within aa108–111 of the thioesterase domain. *Zm*ALT1 reads 108-**R**ATV-111 in this region, while *Zm*ALT3 reads 108-**T**ATV-111 (Figs. 1 and 3). A R108T *Zm*ALT1 mutant (*Zm*ALT1-B) and T108R *Zm*ALT3 mutant (*Zm*ALT3-B) were created via overlap-extension PCR (Figs. 1, 3, and 4, Files S1-S2, Table S1). The *Zm*ALT1-B mutant retained its preference for fully reduced 12–14 carbon acyl-ACPs, but it also displayed increased overall affinity for β-ketoacyl- and 3-hydroxyacyl-ACPs as a result of the R108T substitution (Figs. 3 and 4, Fig. S2, Table S2). This was unexpected, as aa108–111 of *Zm*ALT1-B were now identical to those of *Zm*ALT3, which is not naturally capable of producing MK precursors and 3-OH FAs in *E. coli*. The β-keto fatty acid precursors of 11–13 carbon MKs now made up 17.8 ± 3.3 mol% of the *Zm*ALT1-B mutant’s products in *E. coli* as compared to 11.8 ± 1.5 mol% for wild-type *Zm*ALT1, and 14:1 3-OH fatty acid, which is only a trace product of *Zm*ALT1, now comprised 9.5 ± 1.3 mol% of the *Zm*ALT1-B mutant’s product output (Fig. 4, Fig. S2-S4). This was balanced by a large decrease in this mutant’s affinity for 16-carbon acyl-ACP substrates (Fig. 4, Fig. S2, Table S2). The *Zm*ALT3-B mutant did not display thioesterase activity despite being expressed successfully in *E. coli*, indicating that *Zm*ALT3 cannot accommodate a mutation from Thr108 to Arg108 (Figs. 3, 4, and 5, Fig. S2, Table S2).

The behaviour of these *Zm*ALT1 and *Zm*ALT3 variants generally aligned with that of the *At*ALT and *Mt*ALT variants described previously; mutating aa108–111 could modulate chain-length and oxidation state preferences by adjusting the variant’s affinity for each of its natural substrates, while altering aa31–36 could enable the mutant to act on certain acyl-ACPs or prevent it from binding substrates that the wild-type ALT normally accommodates.

### Residues 78–93 of the ALT hot dog-fold domain can influence substrate chain-length specificity

Aside from 31-CQH[G/C]RH-36 and 108-KXXA-111, no other motifs that consistently assorted with substrate preference in ALT enzymes could be identified through comparative motif searches. Therefore, regions of the thioesterase domain that were generally less well-conserved among paralogous ALTs were prioritized next as mutagenesis targets. Residues 78–93 of the ALT hot dog-fold domain, which span β3-β4, represent one such region, and residues 78–93 of *Mt*ALT1, *Zm*ALT3, and *Zm*ALT1 were exchanged with the corresponding sequence from *Mt*ALT2, *Zm*ALT1, and *Zm*ALT3, respectively (Figs. 3 and 4, File S2).

In all three of the resulting chimeras, *Mt*ALT1-C, *Zm*ALT1-C, and *Zm*ALT3-C, dramatic changes in chain-length specificity were observed. Replacing aa77–93 with sequence from ALTs that prefer 12–16 carbon substrates caused the chain-length preferences of the *Mt*ALT1-C and *Zm*ALT3-C chimeras to shift away from 8:0 acyl chains (Figs. 3 and 4, Fig. S2, Table S2). C12–14 FAs and C16 FAs made up 51.7 ± 6.3 mol% and 36.8 ± 7.9 mol%, respectively, of the products of *Mt*ALT1-C in *E. coli*, and over half of the *Zm*ALT3-C mutant’s output consisted of C12–16 FAs (58.9 ± 2.2 mol%) (Fig. 4, Fig. S2, Table S2). Bacteria expressing the *Mt*ALT1-C mutant also began to produce small but significant amounts (≤3.6 mol%) of the precursors of C11-C15 methylketones, which are natural products of *Mt*ALT2 in *E. coli*. The opposite effect on chain-length specificity was observed in the *Zm*ALT1-C chimera, which adopted preference for 8:0 acyl chains (Figs. 3 and 4, Fig. S2, Table S2). 8:0 FA, which is only a trace product of *Zm*ALT1 in *E. coli* (0.8 ± 0.1 mol% total products), was the most abundant compound produced by the *Zm*ALT1-C mutant, representing 42.9 ± 5.2 mol% of its products (Figs. 3 and 4, Fig. S2, Table S2). While average total lipid accumulation (per mL OD) in *E. coli* cultures expressing the *Zm*ALT3-C chimera was approximately three times greater than in *Zm*ALT3-expressing cultures, bacteria expressing the *Zm*ALT1-C chimera suffered a 20-times decrease in comparison to cultures expressing *Zm*ALT1 (Fig. 4, Table S2). Immunoblotting analysis revealed that *Zm*ALT3-C and *Zm*ALT1-C are expressed at similar levels to their wild-type counterparts in *E. coli*, indicating that these observations reflect changes in mutant thioesterase activity rather than fluctuations in recombinant protein expression accumulation (Fig. S4).

*At*ALT3 and *At*ALT4 only differ at positions 79, 80, and 83 within aa78–93, so these residues were exchanged with those from *At*ALT3 to create the *At*ALT4-C mutant (Figs. 3 and 4, Fig. S2, Table S2). Unlike *Mt*ALT1-C and *Zm*ALT3-C, however, the chain-length preferences of this mutant remained unaltered. *At*ALT4-C continued to produce 8:0 and 6:0 FAs as its major products, in similar proportions to wild-type *At*ALT4 (Fig. 4, Fig. S2, Table S2).

### The 108-KXXA-111 motif and compatible sequence at aa78–93 of the ALT hot dog-fold domain are required to accommodate the 31-CQH[G/C]RH-36 motif

Given that aa78–93 of the ALT catalytic domain exerted influence on chain-length specificity in some cases, we investigated the effects of mutating these residues in conjunction with previously explored motifs. Replacing aa31–36 of the existing *At*ALT4-C and *Mt*ALT1-C chimeras, where aa78–93 had already been mutated, with 31-CQHGRH-36 led to a loss of thioesterase activity (Fig. S2, Table S2). Surprisingly, subsequent introduction of the 108-KXXA-111 motif in these variants, such that it and the 31-CQHGRH-36 motif were both intact, rescued thioesterase activity (Figs. 3 and 4, Fig. S2, Table S2). The re-activated chimeras were named *Mt*ALT1-ABC and *At*ALT4-ABC (Fig. 4, Fig. S2, File S2).

Since *At*ALT3/4 share identical sequence beyond position 83 within aa78–93, but *Mt*ALT1/2 differ at several positions beyond aa83, additional *Mt*ALT1 chimeras were created to assess whether aa84–93 contributed to the restoration of thioesterase activity in the *At*ALT4-ABC and *Mt*ALT1-ABC variants. This was found to be the case, since an *Mt*ALT1 variant where only aa78–83 were exchanged with the corresponding sequence from *Mt*ALT2 alongside aa31–36 and aa108–111 did not show signs of activity (File S2, Fig. S2, Table S2)*.* Thioesterase activity was restored in a chimera where aa78–90, aa31–36, and 108–111 were identical to *Mt*ALT2, however, the productivity of this chimera (*Mt*ALT1-ABC2) was severely compromised (Figs. 3 and 4, Fig. S2, Table S2).

The *At*ALT4-ABC chimera was the first *At*ALT4 variant to have a substantially different product profile from wild-type *At*ALT4 (Fig. 4, Fig. S2, Table S2). While 8:0 FA remained the major product of *At*ALT4-ABC, this variant’s affinity for 12–14 carbon acyl chains increased significantly. It also no longer produced 6:0 FA, which accounts for 33.6 ± 9.3 mol% of the products of wild-type *At*ALT4 (Fig. 4, Fig. S2). The proportion of 3-hydroxy fatty acid products generated by this variant (3.7 ± 1.9 mol%) was now statistically identical to wild-type *At*ALT3 (4.7 ± 1.7 mol%) (Fig. 4, Fig. S2). Interestingly, total FA + MK accumulation in *E. coli* expressing the *At*ALT4-ABC variant was nearly ten times greater than in *At*ALT4-expressing bacteria, increasing to 244.0 ± 15.8 nmol / mL OD from 25.0 ± 2.7 nmol / mL OD, although immunoblotting analysis demonstrated that this was due to increased protein accumulation in *E. coli* (Fig. 4, Table S2, Fig. S4).

Comparison of the activity profiles of the *Mt*ALT1-ABC and *Mt*ALT1-ABC2 chimeras to one another and to the *Mt*ALT1-C mutant also indicated that residues 91 and 93 of the ALT thioesterase domain influence selectivity towards 3-hydroxy acyl-ACP substrates (Fig. 3). *Mt*ALT1-ABC and *Mt*ALT1-ABC2 only differ at these two positions, with the *Mt*ALT1-ABC chimera possessing Phe91 and His93 from *Mt*ALT2 instead of Leu91 and Gln93 (Figs. 3 and 4, Fig. S2, Table S2). 3-OH 14:1 FA, which is the primary product of *Mt*ALT2, made up 16.1 ± 2.0 mol% of products in *E. coli* expressing *Mt*ALT1-ABC, while bacteria expressing *Mt*ALT1-ABC2 only produced it in trace amounts (1.9 ± 0.5 mol%) (Figs. 3 and 4, Fig. S2, Table S2). 3-hydroxy fatty acids were not detected in samples from bacteria expressing the *Mt*ALT1-C variant, which contains aa78–93 identical to *Mt*ALT2, but not the 31-CQHGRH-36 motif.

### Residues 94–96 and 64–67 of the ALT catalytic domain also contribute to oxidation state specificity

Further investigations were conducted with the *At*ALT3/4 pair, as it was unclear why the *At*ALT4-ABC mutant’s substrate preferences still did not resemble those of *At*ALT3 as closely as *Mt*ALT1-ABC resembled *Mt*ALT2. *At*ALT3/4 differ at only 15 positions within their 140aa hot dog-fold catalytic domain, as shown in Fig. 3. Excluding previously explored regions, most differences in catalytic domain sequence between *At*ALT3/4 occur within aa64–67, aa94–96, and aa140–144 (Fig. 3). While *Mt*ALT1/2 share identical sequence at aa64–67 and aa94–96, *At*ALT4 contains the sequences 64-**K**FL**S-67** and 94-**S**I**L**-96, and *At*ALT3 possesses 64**-N**FL**A-67** and 94-**F**I**F-**96 in these regions. Residues 94–96 (D) in the existing *At*ALT4-ABC mutant backbone were replaced with those from *At*ALT3 to create the *At*ALT4-ABCD variant, and both aa64–67 (E) and aa94–96 (D) were replaced with the corresponding sequence from *At*ALT3 to create *At*ALT4-ABCDE (Figs. 3 and 4, File S2). Residues aa140–144 were omitted from further analysis due to a breakdown in sequence conservation and modelling confidence at the ALT C-terminus. As shown in structural models generated by AlphaFold, these residues are typically modelled with low confidence, and sometimes, with disordered secondary structure, resulting in their exclusion from models of the predicted ALT homotetramer (Fig. 1B, Fig. 3, File S1, Fig. S4).

**Fig. 6:**
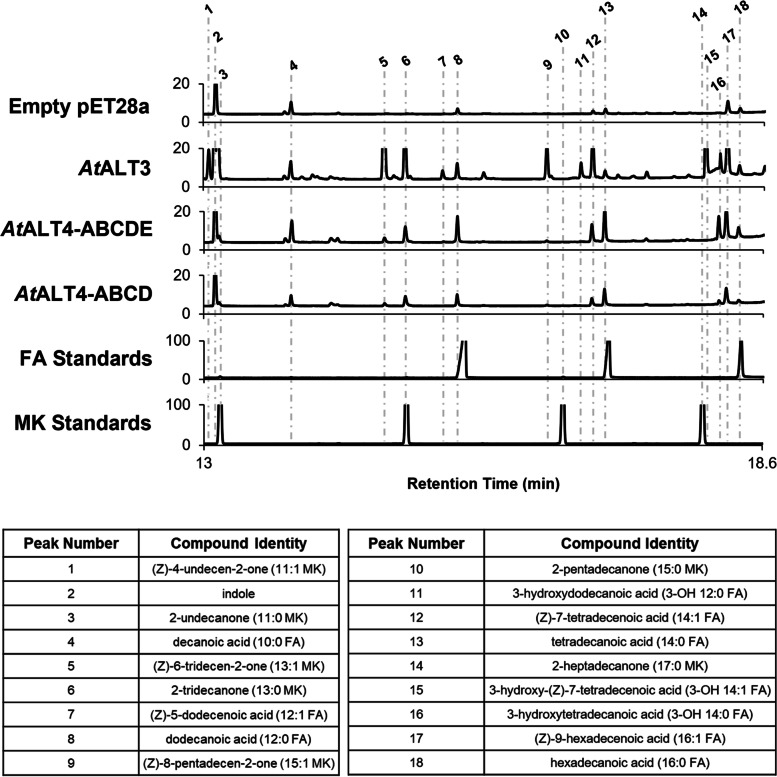
GC-FID chromatograms of secreted lipids from K27(DE3) *E. coli* strains expressing *At*ALT4 variants or *At*ALT3. To create the *At*ALT4-ABCD chimera, sequence encoding residues 31–36, 64–67, 77–93, and 108–111 of the hot-dog fold thioesterase domain of *At*ALT4 was exchanged with sequence encoding the identically numbered residues from *At*ALT3. In addition to the previously listed mutations, residues 94–96 were also replaced with the corresponding sequence from *At*ALT3 in the *At*ALT4-ABCDE chimera. β-keto fatty acids produced by ALTs were decarboxylated to methylketones with heat and acid treatment prior to identification and quantification by GC-MS and GC-FID analysis. Chromatograms of authentic fatty acid and methylketone retention standard mixtures, and of secreted lipids from K27(DE3) *E. coli* harbouring an empty vector, are provided for comparison. Compound identities corresponding to peak numbers are listed below the chromatograms (FA = fully reduced fatty acids, MK = methylketone, 3-OH FA = 3-hydroxy fatty acid)

Although total lipid production by the *At*ALT4-ABCD and *At*ALT4-ABCDE variants in *E. coli* was compromised in comparison to wild-type *At*ALT4, it was apparent that their substrate specificities now approximated *At*ALT3 more closely (Figs. 4 and 6, Fig. S2, Table S2). 14-carbon products were now the most abundant in samples from the bacterial strains expressing *At*ALT4-ABCD and *At*ALT4-ABCDE, comprising 38.8 ± 5.2 mol% and 49.6 ± 14.9 mol% of total products, respectively (Figs. 4 and 6, Fig. S2, Table S2). Notably, these mutants displayed significantly increased affinity for ≥10C β-keto fatty acyl-ACPs, which are the preferred substrates of *At*ALT3 in *E. coli*. Although C13 MK precursors did not represent as substantial a proportion of their product output as in *At*ALT3, these compounds, which are not produced by wild-type *At*ALT4, now comprised 12.8 ± 1.8 mol% and 11.9 ± 2.7 mol% of the products of *At*ALT4-ABCD and *At*ALT4-ABCDE*,* respectively (Figs. 4 and 6, Fig. S2, Table S2). The introduction of aa64–67 (E) from *At*ALT3 into the *At*ALT4-ABCDE mutant also caused its affinity for 3-OH fatty acyl-ACPs to increase significantly. 14-carbon 3-OH FAs accounted for 16.7 ± 7.8 mol% of this variant’s total products in *E. coli* (Figs. 4 and 6, Table S2). Production of 6–8 carbon FAs by strains expressing these variants did not differ from the empty vector control strain.

Interestingly, the *At*ALT4-ABCD and *At*ALT4-ABCDE chimeras also displayed significant alterations in substrate saturation level preference. While most ALTs prefer monosaturated, C14:1 acyl chains over saturated C14 acyl chains in *E. coli*, these variants prefer the latter, as evidenced by GC-FID chromatograms of lipid extracts from bacterial cultures expressing these mutants (Fig. 6, Fig. S2).

### Additional AtALT3/4 chimeras reveal more information regarding the influence of identified regions of the ALT catalytic domain on substrate specificity

Three more *At*ALT4 variants were constructed to assess the combinatorial effects of aa108–111, aa78–96, and aa64–67 of the ALT thioesterase domain on substrate specificity in the absence of the 31-CQH[G/C]RH-36 motif (File S2). Wild-type Met35 was restored within the existing *At*ALT4-ABC, *At*ALT4-ABCD, and *At*ALT4-ABCDE mutant backbones, such that the 31-CQH[G/C]RH-36 motif was no longer present and other mutations remained intact. These chimeras were named *At*ALT4-BC, *At*ALT4-BCD, and *At*ALT4-BCDE (Fig. 4, File S2). The chain-length preferences of *At*ALT4-BC, which contained 108-KGIA-111 and aa78–93 from *E. coli* largely resembled those of the wild-type *At*ALT4 enzyme and the previously described *At*ALT4-B and *At*ALT4-C mutants. However, bacteria expressing *At*ALT4-BC now produced C14:1 3-OH FA and the precursors of C9, C11, and C15 MKs in similar proportions to *At*ALT4-ABC (Fig. 4, Fig. S2, Table S2).

The introduction of 94-**F**I**F**-96 in the *At*ALT4-BCD variant slightly increased its affinity for 12–14 carbon chains while simultaneously decreasing its affinity for 6:0 FA (Figs. 3 and 4). The proportion of 12–14 carbon products generated by *At*ALT4-BCD in *E. coli* increased by approximately 9.0 ± 3.1 mol% compared to the *At*ALT4-BC variant, and 6:0 FA no longer represented a statistically significant proportion of its product output (Fig. 4, Fig. S2, Table S2). The inclusion of 64-**K**FL**A**-67 from *At*ALT3 in the *At*ALT4-BCDE variant reversed the observed changes in chain-length selectivity, and further boosted 8:0 β-keto fatty acid production to 17.0 ± 3.6 mol% of total products, the greatest proportion for any *At*ALT4 variant (Figs. 3 and 4, Fig. S2, Table S2). Unlike *E. coli* expressing *At*ALT4-ABCDE, the strain expressing *At*ALT4-BCDE was incapable of producing 3-hydroxy fatty acids. All three of the above-described chimeras still produced 8:0 FA as their primary product, similar to *At*ALT4, and none were capable of producing C14 β-keto FAs in significant amounts (Fig. 4, Fig. S2, Table S2).

### Computational modelling of ALT variants and in silico docking of the ALT homotetramer with *E. coli* ACPs illustrates how residues shown to influence substrate specificity contribute to acyl binding cavity structure and interactions with ACP partners

**Fig. 7:**
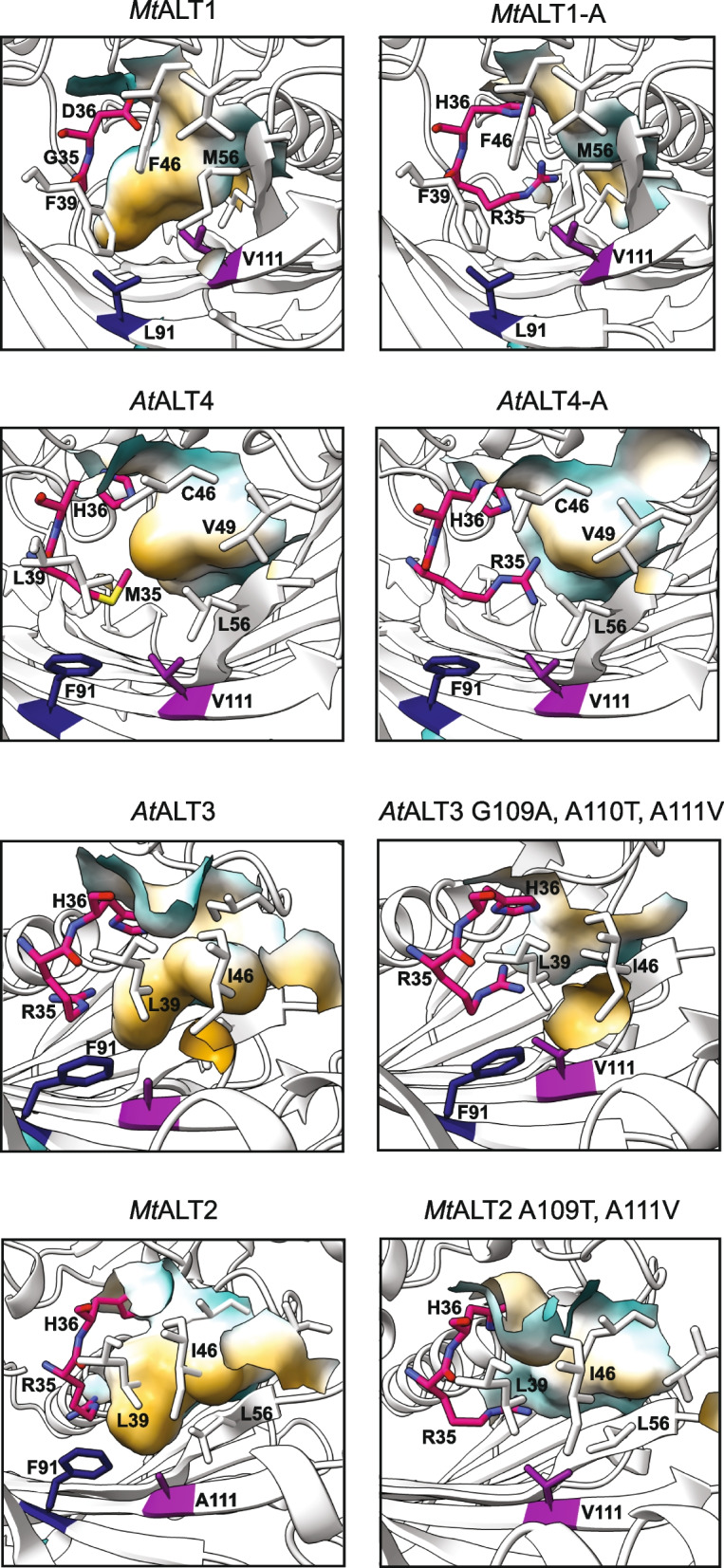
Predicted acyl binding cavity structures of modelled wild-type and mutant ALTs. Computational models of the predicted ALT active unit indicate that introduction of the 31-CQH[G/C]RH-36 motif leads to disruptions in the acyl-binding cavity structure of *At*ALT4 and *Mt*ALT1, while altering the 108-KXXA-111 motif compromises substrate-binding cavity depth in *At*ALT3 and *Mt*ALT2. Ribbon structure of α1-α2 of the hot-dog fold thioesterase domain (aa24–54) is hidden for increased visibility of individual residues. Predicted cavity-forming residues are shown as stick models, and molecular surfaces formed by these residues are coloured according to hydrophobicity (yellow = hydrophobic, white = amphipathic, blue = hydrophilic). Models of wild-type ALT monomers were created by AlphaFold 2.0, and tetramer assemblies were constructed with HSYMDOCK [39–41]. The effects of amino acid mutations were simulated using the FoldX 5.0 suite [54]. Models were visualized in ChimeraX 1.2.5 software [42]

To make more informed predictions regarding which residues are the most important within the structural regions shown to influence ALT substrate specificity, we constructed models of select chimeric ALTs and conducted in silico docking simulations between the ALT homotetramer and *E. coli* ACP partners [39–41, 43, 44, 49–54]. According to computational models of the wild-type ALTs used to create the chimeras described above, residues in areas that influence ALT substrate preferences, specifically residues 35 and 36 on the central α-helix, and residues 91 and 111 nearby on the β-sheet, contribute to a central hydrophobic pocket likely to be the thioesterase’s acyl binding cavity (Figs. 3 and 7). Alignment of these models with the known crystal structure of the *E. coli* YbgC thioesterase supports this, with predicted cavity-forming residues in ALT models aligning structurally with cavity-forming residues in *Ec*YbgC (Fig. 7, Fig. S2 D-E) [45]. Several predicted cavity-forming residues in ALTs are also structurally analogous to known acyl-binding cavity residues in the N-terminal hot-dog fold domain of the *Umbellularia californica* FatB thioesterase (Fig. S2 D-E) [55, 56].

The FoldX 5.0 suite was used to simulate the effects of certain mutations on the modelled ALT catalytic domain structure [54]. Comparison of models of the inactive *At*ALT4-A and *Mt*ALT1-A mutants to their wild-type counterparts showed that major disruptions to the predicted acyl binding cavity structure occur as the result of an M35R substitution in *At*ALT4, or the introduction of 34-GRH-36 into *Mt*ALT1 (Fig. 7). Hypothetical *At*ALT3 and *Mt*ALT2 variants where 108-KGIA-111 of *At*ALT3 were replaced with 108-KATV-111 from *At*ALT4, and 108-KATA-111 of *Mt*ALT2 were replaced with 108-KTTV-111 from *Mt*ALT1, were also modelled (Fig. 7). In these variants, acyl binding cavity depth also appears severely compromised due to displacement of the R35 and H36 side-chains resulting from the introduction of Val111 (Fig. 7).

**Fig. 8:**
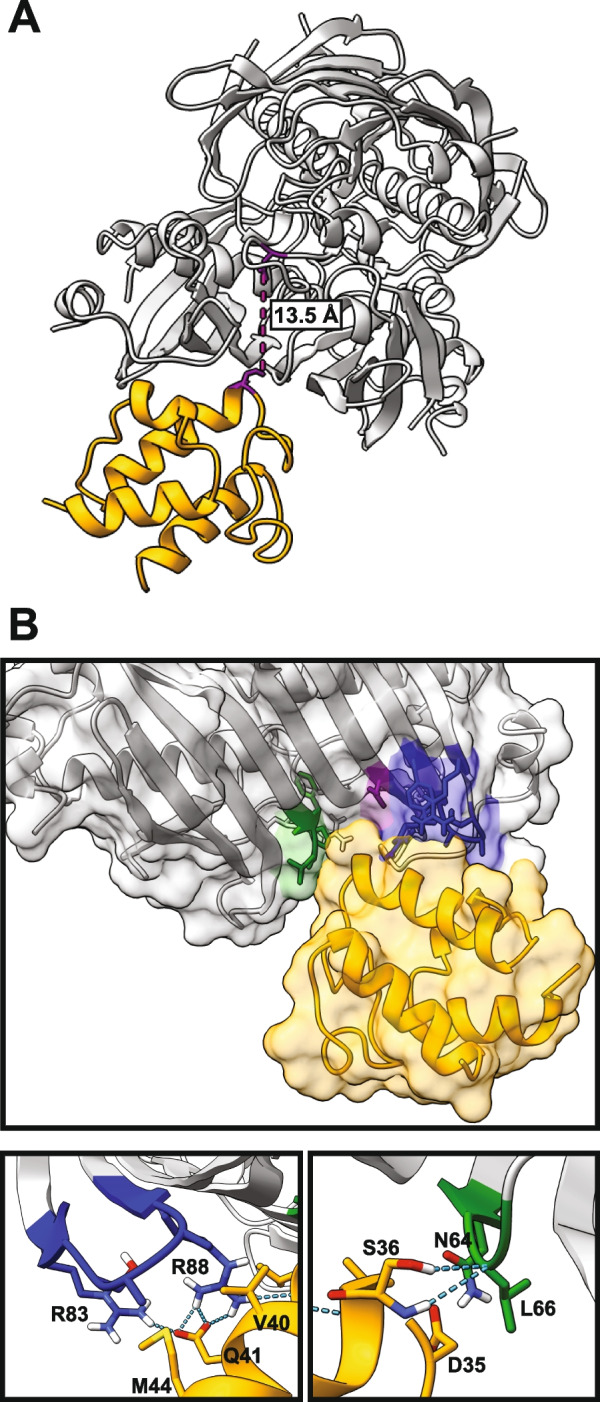
Residues within regions that influence ALT substrate specificity may contribute to interactions with ACPs. **A** Ribbon model of the maximum likelihood docking confirmation of *E. coli* acyl carrier protein (yellow, PDB ID 2FAC) with the *At*ALT4 homotetramer (grey) [51]. The predicted active site residue of *At*ALT4, Asp17, is located at 13.5 Å from of S36 on ACP, which is necessary to accommodate rotation of the tethered acyl chain carrier by ACP about the phosphopantethiene arm [33, 34]. Docking of *At*ALT4 with *E. coli* ACPs was performed using the ClusPro server, and docking conformations were visualized in ChimeraX 1.2.5 software [42, 49, 50]. **B** Molecular surface model of the predicted ACP docking site of *At*ALT4. Residues within aa78–93 (blue), aa64–67 (green), and aa108–111 (purple) of the ALT thioesterase domain that form part of the interaction interface with docked ACP (yellow) are coloured and shown as stick models. Magnified views show inter-chain hydrogen bonds, represented as blue dashed lines, between residues of *At*ALT4 and of docked *E. coli* ACP

The maximum likelihood docking conformation obtained through docking simulations of the modelled *At*ALT4 homotetramer with *E. coli* holo-ACPs (PDB IDs 2FAC, 2FAE, and 3EJB) demonstrates that residues within regions that influence ALT substrate preferences, specifically aa83–88 and T110 from one monomer subunit, and aa64–67 from the neighbouring subunit, are part of a positively charged patch on the thioesterase surface that forms the predicted interaction interface with acyl carrier protein (Fig. 8) [49–53]. Residues R83, R88, and N64 are shown in this model to contribute to inter-chain hydrogen-bonding interactions with residues belonging to ACP (Fig. 8B). The changes in chain-length and oxidation state specificities that are observed when these regions of the ALT catalytic domain are mutated may result, in part, from effects on how they interact with ACPs that deliver substrates.

## Discussion

### Chimeric enzymes inform our understanding of ALT substrate selectivity

The behaviour of chimeric ALT enzymes in *E. coli* provides much insight into what areas of the ALT hot dog-fold domain are involved in this process, and why certain activity patterns are more common among ALTs. Considering the product profiles of wild-type and chimeric ALTs together illustrates that residues 31–36 of the central α-helix primarily influence chain-length preference, rather than oxidation state specificity as initially hypothesized. It stands to reason that this region would affect length preference given that residue 35 of the central ALT α-helix is structurally analogous to T137 of *Uc*FatB, a key cavity-forming residue that can alter chain-length specificity when mutated (Fig. S2) [55, 56]. Chain-length specificity is necessarily a function of acyl-binding cavity depth in all acyl thioesterases that share the core hot-dog fold domain structure. Functional mutants with the 31-CQH[G/C]RH-36 motif consistently displayed strong affinity for 12–14 carbon acyl chains, such that ≤10-carbon substrates were heavily disfavoured (Fig. 4). However, the *Mt*ALT1-ABC2 and *At*ALT4-ABC mutants, which possessed the 31-CQH[G/C]RH-36 motif, showed no greater preference for partially reduced substrates than the *Mt*ALT2-C or *At*ALT4-BC mutants, which did not possess this motif (Fig. 4). Instead, interactions between regions of the ALT hot dog-fold domain β-sheet identified in this study (aa108–111, aa78–96, and aa64–67) dictate whether the enzyme is able to accept substrates of partially reduced oxidation states. For example, the *At*ALT4-BCDE chimera, where all of these regions have been mutated, displayed markedly increased affinity for β-keto FAs, while mutating aa108–111 or aa78–93 of *Mt*ALT1 and *Zm*ALT1 allowed the resulting variants to act on partially reduced substrates that the wild-type enzymes could not accommodate (Fig. 4). It is likely that the sequence features required for an ALT to accommodate the 31-CQH[G/C]RH-36 motif and retain thioesterase function are what grant an ALT the ability to cleave partially reduced acyl-ACP chains. This combined with the 31-CQH[G/C]RH-36 motif’s dramatic effect on chain-length specificity would, in part, account for why ALTs that naturally possess this motif consistently prefer 14:1 β-keto and 14:1 3-OH fatty acyl-ACPs in *E. coli* [24]. The biological significance of this pattern has yet to be determined.

Computational modelling of the ALT thioesterase domain also provides a possible explanation for the natural co-occurrence of the 31-CQH[G/C]RH-36 and 108-KXXA-111 motifs, and the observed inactivity in *E. coli* of ALT variants containing only one these motifs. The side-chain of Val111, which is conserved in most ALTs that do not possess 31-CQH[G/C]RH-36, is likely too large to accommodate 35-RH-36 within the catalytic domain core, leading to structural disturbances that would result in enzyme inactivity (Fig. 7). The small side-chain of Ala/Gly 111 in ALTs containing the 31-CQH[G/C]RH-36 motif would therefore be needed to leave space for 35-RH-36. This interaction is similar to what occurs in the N-terminal domain of FatB-type thioesterases. Residue 111 of the ALT catalytic domain is also structurally analogous to the cavity-forming residue S219 in *Uc*FatB, which occludes the acyl-binding cavity and greatly reduces enzyme activity when mutated to bulky tryptophan (Fig. S2) [55, 56]. Although aa78–93 of the thioesterase domain also play a necessary role in accommodating the 31-CQH[G/C]RH-36 motif, as demonstrated by the *At*ALT4-ABC, *Mt*ALT1-ABC, and *Mt*ALT-ABC2 variants, why this is so was not obvious from structural models. However, *At*ALT3 and *At*ALT4 only differ at R/M35 within aa31–36, suggesting that interactions between aa35 and other residues within aa108–111 and aa78–93 are critical to thioesterase function (Fig. 3).

The behaviour of ALT variants characterized in this work points toward Lys64 being necessary for 3-OH FA production. Out of all characterized ALT-type thioesterases from diverse plant species, only *At*ALT4 and *Zm*ALT4 possess Asn64 in place of a conserved Lys64 residue in the hot dog-fold domain (Fig. 3, File S1). Neither generate 3-OH FAs in *E. coli* [24]. An *At*ALT4 variant could only produce 3-OH FAs upon introduction of Lys64, while the *Mt*ALT1-ABC and *Zm*ALT-B chimeras, which already possessed this residue, were capable of producing substantial amounts of 14-carbon 3-OH FAs (Figs. 3 and 4, Files S1-S2, Fig. S2). Although fully reduced FAs and MK precursors from 6 to 16 carbons in chain-length are observed as products of non-recombinant and mutant ALT-type thioesterases, 3-OH FA production by ALTs appears to be restricted to the 12–14 carbon chain-length range [24]. It is possible that ALTs simply cannot accommodate ≤ C10 3-hydroxyacyl-ACP substrates without introducing unnatural mutations, or that ≤ C10 3-hydroxyacyl-ACPs are not abundant enough in *E. coli* acyl-ACP pools for these products to be detected.

Curiously, none of the chimeric ALT enzymes tested here produced MK precursors as their primary products, although these are the major products of numerous ALTs found in nature, such *Sh*MKS2, its characterized orthologues in the Solanaceae, and *At*ALT3, when they are expressed in *E. coli* [20, 23–25]. While MK precursors made up a significant proportion of the products of certain chimeric ALTs, such as *At*ALT4-ABCDE and *Mt*ALT1-ABC2, total productivity of these variants was usually compromised (Figs. 4 and 6). This indicates that other determinants of ALT oxidation state specificity have yet to be elucidated, and that they likely form stabilizing interactions with aa31–36 of the hot dog-fold domain. In the case of the *At*ALT4-ABCDE mutant, however, substrate availability could also be a major contributing factor to its low productivity. The saturated ≥C10 acyl chains that this variant prefers are much less readily available in *E. coli* acyl-ACP pools than the monounsaturated ≥C10 acyl chains favoured by heterologously expressed ALT-type thioesterases [6]. Further targeted mutagenesis experiments involving *Sh*MKS2 and its homologues may assist in features of the ALT catalytic domain that contribute to specificity towards β-keto acyl-ACP substrates.

### Knowledge gained from the study of ALT-like thioesterases is applicable to other bacterial single hot dog-fold thioesterases that are widely used in metabolic engineering strategies

Bacterial single hot dog-fold acyl-CoA thioesterases belonging to the same structural clade as ALTs are used extensively in metabolic engineering pipelines to produce medium-chain, β-keto, hydroxy, and fully reduced fatty acids in microbial systems [26, 32, 55–57]. However, little work has been done to understand the biochemical underpinnings of substrate specificity within this enzyme clade [35]. Despite plant ALTs being acyl-ACP thioesterases, not acyl-CoA thioesterases like other enzymes that share the same core catalytic domain structure, information regarding the determinants of ALT substrate specificity could still be extended to related bacterial single hot dog-fold thioesterases [35]. Amino acids that demonstrably modified substrate specificity in the chimeric ALTs and were predicted to be acyl binding cavity-forming residues in computational models, align consistently to cavity-forming residues in the crystal structure of *E. coli* YbgC (Fig. 7, Fig. S1). This supports the notion that structural features that dictate ALT substrate preferences and activity may also affect these properties in related bacterial single hot dog-fold thioesterases. Knowledge gained from the study of ALTs can be applied to the construction of optimized mutant versions of related bacterial thioesterases, such as FadM, YciA, and YbgC-type thioesterases, which have already shown promise in metabolic engineering applications [26, 32, 35, 57–59].

### ALT variants have unique potential as metabolic engineering tools in plant and microbial hosts

Given their unique substrate specificities compared to other plant acyl-ACP thioesterases (e.g. FAT-type), ALTs themselves are also attractive from a biotechnological standpoint. Heterologous expression of medium-chain specific FatB thioesterases originating from plants such as *Cuphea* spp. and *Umbellularia californica,* is common in metabolic engineering pipelines to produce medium-chain fatty acids, fatty alcohols, and methylketones in microbes, and in the development of transgenic oilseed crops that accumulate high medium-chain content in seed triacylglycerols [12–18, 26, 36, 59, 60]. ALTs also naturally lend themselves to these purposes. The non-recombinant and chimeric ALTs described in this work overlap in their specificities with seed expressed medium-chain FatB-type thioesterases [61]. Nonetheless, certain unique chain-length specificity patterns are observed among ALTs; for instance, certain ALTs possess high affinity for hexanoyl-ACP, while no FatB-type thioesterases with appreciable activity toward 6:0 chain-lengths have been identified [61]. Developing ALT variants with strong specificity towards hexanoyl-ACP could therefore be especially useful for future biotechnological applications. The generally broader range of fatty acid chain-lengths output by ALT-type thioesterases may also be beneficial for certain applications. For instance, medium-chain triglyceride mixtures containing C6-C12 chain-lengths are administered in obesity management and the treatment of metabolic disorders [62].

While heterologous overexpression of acyl-ACP thioesterases is sufficient to produce fully reduced fatty acids of desired chain-lengths, generating other medium-chain oleochemicals, such as MKs and 3-OH fatty acids, typically necessitates the chemical conversion of these free fatty acids. However, this introduces a redundant cycle of acyl-ACP thioester cleavage and reactivation to acyl-CoA thioesters [26, 32]. With their ability to produce β-keto and 3-OH fatty acids directly from intermediates of fatty acid biosynthesis, overexpression of ALT-like thioesterases would eliminate the need for this repetitive step in the production of valuable medium-chain compounds. Highly productive recombinant ALTs with specificity for medium-chain 3-hydroxyacyl-ACPs would be especially useful in metabolic engineering contexts involving bacterial strains, since often-used bacterial acyl-CoA thioesterases such as TesB, YigA, and FadM, only produce these compounds as minor byproducts [59]. In addition to their value as surfactants and additives to cosmetics, biodiesel and other consumer products, medium-chain 3-OH FAs are building blocks of poly-3-hydroxyalkanoates, biodegradable polymers with desirable plasticizer and elastomer properties [59]. Developing recombinant ALT enzymes with greatly increased 3-OH FA productivity could be achievable in the near future, as certain features of the ALT catalytic domain that can be manipulated to boost affinity for 3-OH fatty acyl-ACPs were uncovered in the present study. The capability of ALTs to act on 3-hydroxy-ACP intermediates of fatty acid biosynthesis could also prove useful in plant systems; however, while 3-hydroxy fatty acids are well-known components of bacterial endotoxins, they are rarely documented as plant metabolites outside of fatty acid biosynthesis, especially in the medium-chain length range [63–65]. It is unknown whether recombinant ALTs with preference for 3-hydroxyacyl-ACPs in *E. coli* would retain these preferences *in planta*.

If recombinant ALT enzymes are to be developed for use as metabolic engineering tools, the potential for heterologously expressed ALTs to behave in unexpected ways must also be considered. Overexpression of select ALTs in the seeds of *Arabidopsis thaliana* and *Camelina sativa*, as well as in *Nicotiana benthamiana* leaves demonstrated that their chain-length and oxidation state preferences are very similar in *E. coli* cells versus plant tissues [66]. While this work suggested that ALTs behave similarly whether they are receiving substrates from ACPs of plant or bacterial origin, the possibility that alternate substrate specificity patterns may arise when ALTs interact with ACPs from other species cannot be discounted. Recent work by Sztain et al. (2021) demonstrated that acyl carrier proteins communicate what chain length they are delivering to potential partner enzymes, such as thioesterases, through highly specific protein-protein interactions [67]. When a partner enzyme detects that an ACP is delivering a suitable substrate, the tethered acyl chain is translocated from the ACP core to the active site of the partner enzyme [67]. While ALTs would be equipped to reliably interpret substrate information communicated by plastidial acyl-ACPs in plant tissues where they are naturally expressed, structural differences in ACPs from other host species could create the possibility for substrate identity to be “misread”, resulting in changes to substrate specificity and enzyme activity [67]. Additional considerations must be made in microbial systems, where processes involving fatty acyl-ACPs and fatty acyl-CoAs are not segregated to distinct cellular compartments as they are in plants [5, 6].

### Decoding individual-residue contributions to ALT substrate specificity will allow for rational design of recombinant ALTs for distinct biotechnological applications

Features of the ALT hot dog-fold domain highlighted in this study likely affect substrate specificity in most ALTs, and these amino acids can be manipulated to broadly control the substrate preferences of ALT enzymes as demonstrated here. For instance, chain-length preferences can be directed towards C6–8 or C12–14 acyl-ACPs, products of partially reduced oxidation states can be reduced or eliminated, or affinity for 3-OH acyl-ACPs can be greatly increased by manipulating the regions of the ALT catalytic domain described in this work (Fig. 4). This is a promising step towards rational design of ALT enzymes with product profiles that are suited to specific industrial applications, however, fine-tuning recombinant ALTs to output a narrow range of products of interest will require a better understanding of per-residue contributions to ALT substrate specificity. Thioesterase-ACP docking simulations involving modelled ALT variants, and substrate-fitting studies with attached acyl chains, will allow for the generation of more detailed hypotheses regarding the roles of individual residues in shaping ALT function. Engineering ALT variants with chain-length specificities that deviate from natural trends (i.e. preference for C8 acyl-ACP, C12-C14 acyl-ACPs, or a broad mixture of both) may also require the introduction of residue substitutions that are uncommon or even absent from ALTs found in nature.

As industrial applications demand enzymes that generate products of interest in large quantities, improving the productivity of ALT-type thioesterases is another critical goal that must be pursued in order to harness their biotechnological potential. While certain regions of the ALT hot-dog fold domain identified here are linked to increased production of certain compounds, such as 8:0 FA, C12–14 FAs, and C14 3-OH FAs, it is more difficult to comment on how they affect total thioesterase productivity due to differences in recombinant protein expression levels, and the sometimes inconsistent effects of mutating these regions on total FA + MK production by ALTs from different plant species (Fig. 4, Fig. S4). Moving forward, absolute, rather than relative quantification of fatty acids and methylketone precursors generated by promising ALT variants with improved productivity and desired specificities will provide a more informative assessment of the contributions of individual residues and motifs toward thioesterase productivity.

Randomized mutagenesis within regions of the hot dog-fold domain identified in this study is likely the most effective means of obtaining useful, highly active ALT variants for further experimentation, and gaining insight into how the chemical properties of individual residues contribute to an ALT’s substrate preferences. However, such an approach depends on a quick and reliable mutant screening method. The development of a high-throughput screening method capable of detecting increases in the production of 3-OH FAs and MK precursors, or differentiating between different 3-OH FA and MK chain-lengths, would be an invaluable asset for the construction of a collection of mutant ALTs that could be of use in various biotechnological contexts.

## Conclusions

In this work, we uncover several structural regions that function as core determinants of substrate specificity in ALT-type thioesterases, an unusual family of medium-chain acyl-ACP thioesterases from plants that act on substrates of multiple chain-lengths and oxidation states. By exchanging sequence fragments encoding these regions between pairs of ALT enzymes with different substrate preferences and profiling the products of the resulting chimeric thioesterases in *E. coli*, we demonstrated that mutating these regions of the ALT catalytic domain results in substantial changes to chain-length and oxidation state specificity. Also, these changes are largely consistent among ALTs originating from diverse plant species. Chain-length and oxidation state preferences were found to be coupled among ALT-like thioesterases found in nature, with most ALTs that primarily act on β-keto and 3-hydroxy acyl-ACP intermediates in *E. coli* also preferring 12–14 carbon chain-lengths. We determined through motif-swapping and computational modelling that this is due to the presence of two short interacting motifs in ALTs that contribute to the catalytic domain core, and their interaction with a third, critical region predicted to also participate in ACP docking. The behaviour of chimeric ALTs also provided evidence that it is possible to tune ALT chain-length specificity and oxidation state preferences separately. By manipulating the regions of sequence identified in this study, it is possible to adjust an ALT’s specificity for 6–8 carbon versus 12–14 carbon acyl chain-lengths, and its selectivity for fully reduced or 3-hydroxyacyl-ACPs. The capability of ALT enzymes to directly produce both fatty acids and other industrially valuable medium-chain oleochemicals is advantageous in many biotechnological applications. The results of the targeted mutagenesis experiments described here ultimately show that engineering recombinant ALT enzymes that are highly active and specific for products of interest is a feasible goal that could be met by further investigating the underlying mechanisms of substrate specificity in these unique plant acyl-ACP thioesterases.

## Methods

### Sequence data collection

The nucleotide coding sequences and amino acid sequences of ALT-type thioesterases were obtained from the NCBI GenBank and the Cannabis Genome Browser Gateway (http://genome.ccbr.utoronto.ca/cgi-bin/hgGateway). GenBank accession numbers of sequences mentioned in this article are as follows: *A. thaliana ALT1* (NM_103226.3), *A. thaliana ALT2* (BT024833.1), *A. thaliana ALT3* (NM_105497.5), *A. thaliana ALT4* (NM_001334359.1), *Z. mays ALT1* (XM_008670822.4), *Z. mays ALT2* (NM_001326549.1), *Z. mays ALT3* (XM_008647798.1), *Z. mays ALT4* (XM_008647802.3), *G. max ALT1* (NM_001377384.1), *V. vinifera ALT1* (XM_002283509.4), *O. sativa* subsp. *japonica ALT1* (XM_473441.1), *B. distachyon ALT1* (XM_003563133.4), *B. distachyon ALT2* (XM_003581459.4), *M. truncatula ALT1* (XM_024783401.2), *M. truncatula ALT2* (XM_003614577.4), *S. melongena MKS2–2* (MK990609.1), *S. hirustum* subsp. *glabratum MKS2* (GU987106.1) and *C. reinhardtii ALT1* (XM_043061639.1). *C. sativa ALT1* has accession number PK15701.1 in the Cannabis Genome Browser.

### Assembly of DNA constructs for the expression of chimeric ALT proteins in K27(DE3) *E. coli*

Double restriction digests of pET-28a plasmid DNA using restriction endonucleases *Bam*HI and *Hind*III were prepared. Digested plasmid DNA was run on a 0.8% agarose gel, and the linearized plasmid was excised and purified using a NucleoSpin Gel and PCR Purification kit (Macherey-Nagel).

DNA sequences encoding the *At*ALT3-A, *At*ALT4-A, *At*ALT4-B, *Zm*ALT1-B, *Zm*ALT3-B, and *Mt*ALT1-B mutants were created by overlap-extension PCR using primers listed in File S1 and previously made constructs described in Kalinger et al. (2018) as templates [24]. These constructs contained the wild-type ALT protein coding sequence (*At*ALT3: NM_105497.5, *At*ALT4: NM_001334359.1, *Zm*ALT1: XM_008670822.4, *Zm*ALT3: , *Mt*ALT1: XM_003614577.4, *Mt*ALT2: XM_024783401.2) in the pET-28a expression vector, truncated to exclude the coding region for the N-terminal region encoding the plastid targeting sequence. Coding sequences of the *At*ALT4-AB, *At*ALT4-AC, *At*ALT4-ABC, *At*ALT4-ABCD, *At*ALT4-ABCDE, *Mt*ALT1-AB, and MtALT1-AC mutants were assembled from DNA fragments amplified from previously made mutant constructs, using PCR primers listed in File S1. DNA sequences encoding all other mutant ALTs described in this study were chemically synthesized. 16-bp extensions were added to the 5′ and 3′ ends of each ALT-encoding sequence, such that the DNA could be ligated into the above digested and purified pET28a plasmid DNA using the In-Fusion High-Speed Cloning Kit (Takara-Clontech). The 16-bp extensions were complementary to the pET-28a vector sequence at the site of linearization and introduced a *Bam*HI restriction site immediately before the start codon of each gene and a *Hind*III restriction site after the stop codon. Expression in the pET-28a vector also introduced a T7-epitope tag at the N-terminus of the ALT protein.

The isolated plasmid DNA was then transformed into the K27(DE3) strain of *E. coli* [48]. The presence of the DE3 lysogen allows for isopropyl-β-D-1-thiogalactopyranoside (IPTG)-inducible expression of T7 RNA polymerase, which subsequently drives expression of the *ALT* transgene on the pET-28a plasmid [48].

### Extraction of fatty acids produced by ALT-expressing E coli

Fully reduced, 3-hydroxy, and β-keto fatty acids produced by ALT-expressing *E. coli* were extracted and profiled as previously described in Yu et al. (2010)*,* with some modifications [23]. K27(DE3) *E. coli* transformed with pET-28a (empty vector) or with pET-28a containing wild-type or chimeric *ALT* coding sequences, were grown in 50 mL of kanamycin-containing LB media at 37 °C to an OD_600_ of ~ 0.4. Protein expression was then induced by the addition of isopropyl-β-D-1-thiogalactopyranoside (IPTG) to a final concentration of 0.5 mM. Induced cells were then grown for 20 h at 18 °C with 200 rpm shaking.

Bacterial cultures were harvested in glass tubes via centrifugation at 4000 x *g* for 5 min. Two mL of culture supernatant was mixed with 2 mL of 20 mM H_2_SO_4,_ and 2 μL of a 20 μg μL^− 1^ solution of heptadecanoic acid (17:0 FA) dissolved in toluene was added to each sample as an internal standard for a total of 40 μg 17:0 FA per sample. Samples were then incubated at 75 °C for 30 min to decarboxylate β-keto fatty acids in culture media to methylketones. After cooling the samples at room temperature, lipids were then extracted into 150 μL hexane by vortexing vigorously for 15 s. One μL of the upper hexane layer was used for gas chromatographic analysis.

### Identification and quantification of fatty acids and methylketones produced by ALT-expressing *E. coli* via GC-FID and GC-MS

Lipids were profiled using an Agilent 7820A gas chromatograph equipped with an HP-5MS column (30 m length, 0.25 mm inner diameter, 0.25 μm film thickness) and a flame ionization detector. Samples were injected with a 7:1 split ratio, with the injector temperature maintained at 250 °C and the detector temperature at 325 °C. The carrier gas was helium at a constant flow rate of 2 mL min^− 1^. The column oven temperature was initially held at 50 °C for 8 min, then increased at a rate of 15 °C per min up to 325 °C, which was held for 4 min. Peaks were integrated using Agilent Technologies OpenLab CDS ChemStation software and normalized with respect to the 17:0 FA internal standard peak area, and the amount of each fatty acid and methylketone species in culture media was calculated in units of nmol per unit OD and mol%. Fatty acid and methylketone species were identified by electron impact (EI) GC-MS (ionization energy: 70 eV) and by comparison to authentic retention standards. Representative GC-FID traces are depicted in Fig. S2. The temperature program, column, and carrier gas flow rate used for GC-MS analysis were the same as described above for GC-FID. Mass spectra of fully reduced fatty acids and saturated methylketones were compared to standard spectra in the National Institute of Standards and Technology’s NIST 17 Mass Spectral Library. Monounsaturated methylketones were identified by comparison to spectra previously reported by Goh et al. [30]. All mass spectra used for compound identification are shown in Fig. S3. The chain lengths of free 3-hydroxy fatty acids were difficult to determine via EI GC-MS due to absence of the molecular ion and low abundance of larger m/z fragments. Therefore, 3-hydroxy fatty acids were identified via GC-MS of silylated samples as described in the section below.

For all *E. coli* strains expressing wild-type or mutant ALTs, or harbouring an empty pET-28a vector, fatty acids secreted into culture media and methylketones derived from secreted β-keto fatty acids were quantified using spent media from three individual bacterial cultures. Fatty acid and methylketone quantities were expressed as the average across cultures (*n* = 3). Data normality was confirmed by Shapiro-Wilk’s test (*p <* 0.05). The statistical significance of differences in fatty acid and methylketone production in *E. coli* strains expressing ALT-like thioesterases as compared to a strain harbouring an empty pET-28a vector were determined via a right-tailed Student’s *t*-test (*p <* 0.05). If sample variances were determined to be equal by Levene’s test (*p* ≥ 0.05), the significance of differences in fatty acid and methylketone accumulation among groups of strains expressing mutant and wild-type ALT enzymes were assessed by one-way ANOVA followed by Tukey’s HSD test (*n* = 3, 95% family-wise confidence level, *p <* 0.01). Otherwise, the statistical significance of differences among strains was determined by a Kruskal-Wallis test followed by Dunn’s test of significance (*n* = 3, 95% family-wide confidence level, *p <* 0.01). All raw data used for quantification of fatty acids and methylketones produced by ALT-expressing *E. coli*, and results of statistical analyses, are shown in Table S2.

### Silylation of fatty acids produced by ALT-expressing E coli to assist identification of 3-hydroxy fatty acids

ALT protein expression was induced in *E. coli* and secreted lipids were extracted as described above. Hexane extracts were evaporated to near dryness under a gentle stream of nitrogen gas. Dried samples were then resuspended in 100 μL N,O-Bis(trimethylsilyl)trifluoroacetamide (BSTFA) with 100 μL pyridine as a catalyst. Samples were incubated at 115 °C for 15 minutes to silylate carboxyl and secondary alcohol groups, after which they were evaporated again to near dryness under nitrogen gas. Samples were resuspended in 200 μL hexane, and 1 μL of each resuspended sample was used for GC-MS analysis. The same temperature program, column, and carrier gas flow rate described above was used for GC-MS analysis. The 2-trimethylsilyl (TMS) derivatives of 3-hydroxy fatty acids were identified by comparison with standard spectra in the NIST 17 Mass Spectral Library, with the exception of the 2-TMS derivative of 7(Z)-3-hydroxytetradec-7-enoic acid (3-OH 14:1 FA), which is not listed. 7(Z)-3-hydroxytetradec-7-enoic acid was identified from the molecular ion present in the mass spectrum of its 2-TMS derivative (m/z = 386), and fragments characteristic of the TMS derivatives of both a 3-hydroxy fatty acid (m/z = 73, m/z = 147, m/z = 233, M^+^ − 31) and a monounsaturated fatty acid (M^+^ − 169, M^+^ − 90). Peak size and elution order in silylated samples was used to confirm 3-hydroxy fatty acid peak location and identity in samples that did not undergo silylation.

### Confirmation of ALT-like protein expression via Tris-Tricine SDS-PAGE and immunoblotting

ALT protein expression was induced in K27(DE3) *E. coli* cells as described previously. Cells from 5 mL of culture were harvested via centrifugation for 10 min at 2000 x *g,* and resuspended in 1 mL of freshly prepared Tris-HCl lysis buffer (25 mM Tris, pH 7.5, 10% v/v glycerol, 2 mM EDTA, 2 mM PMSF, 1% v/v Bio-Rad Protease Inhibitor Cocktail). Cells were placed on ice and lysed by 3 × 15 s sonication cycles, with 30 s wait time between cycles. The total protein concentrations of cell lysates were determined using a Bio-Rad protein assay kit. Total protein concentration was the average of triplicates of each sample. Tris-Tricine SDS-PAGE of crude cell lysates was then performed according to Khuat et al. (2019), with 10 μg of total protein loaded per sample lane [25]. Proteins were then transferred onto nitrocellulose membranes at 270 mA for 25 min. Membranes were incubated in Ponceau S staining solution (0.5% w/v Ponceau S, 5% v/v glacial acetic acid in distilled H_2_O) for 10 min, de-stained in distilled water for 10 min, and imaged under white light prior to blocking and immunodetection. Membranes were then incubated in bovine serum albumin (BSA) blocking solution (3% w/v BSA in Tris buffered saline + 0.01% Tween-20) for 1 h at room temperature with gentle shaking. The blocked membrane was probed with mouse monoclonal anti-T7 tag primary antibody (Millipore Sigma catalogue no. 69522, lot no. 3020009, diluted 1:30000) overnight at 4 °C. The membrane was then probed with goat anti-mouse horseradish peroxidase-conjugated secondary antibody (Millipore Sigma catalogue no. 12–349, lot no. 3174787, diluted 1:250000) for 1 h at room temperature with gentle shaking. Antibody binding was detected with SuperSignal West Pico Plus Chemiluminescent Substrate (Thermo Fisher), and images were captured using Bio-Rad ImageLab 6.1 software.

### Structural modelling of ALT proteins

The modelled three-dimensional structures of *Arabidopsis thaliana* ALT3/4 (UNIPROT IDs Q8W583, F4HX80), *Medicago truncatula Mt*ALT1/2 (UNIPROT IDs G7K1I0, G7K1I9) and *Zea mays* ALT3 (UNIPROT ID A0A1D6HF47), were retrieved from the AlphaFold Protein Structure Database (https://alphafold.ebi.ac.uk/) [39, 40]. The structure of *Zea mays* ALT1 was modelled using the AlphaFold CoLab web notebook (https://colab.research.google.com/github/deepmind/alphafold/blob/main/notebooks/AlphaFold.ipynb) [39, 40]. Regions of each model with poor per-residue confidence scores (plDDT < 70) or that presented with disordered secondary structure were removed from the models using UCSF Chimera 1.15 software [43]. This excluded the predicted N-terminal plastid targeting peptide sequence and C-terminal residues beyond position 136 of the consensus ALT thioesterase domain sequence. As shown in a protein sequence alignment of ALT-like thioesterases characterized in *E. coli*, homology among ALTs deteriorates beyond aa137 (File S1) [37]. This nonconservative region is variable in length, and even absent in certain ALTs with demonstrated activity towards acyl-ACPs of various chain lengths [24].

The global and local quality of the monomeric models output by AlphaFold following omission of low-confidence regions was estimated using the Discrete Optimized Protein Energy (DOPE) quality scoring metric in Chimera 1.15’s MODELLER 10.1 extension [43]. All models had initial z-DOPE scores < − 1.0, which is indicative of a reliable overall fold prediction. Ramachandran and rotamer outliers, steric clashes, and other geometric outliers were detected using MolProbity 4.2 [44]. Regions with drops in local model quality and outlier residues were then selectively minimized in UCSF Chimera 1.15 [43]. Any Ramachandran and rotamer outliers that persisted after selective minimization were corrected manually in Chimera, and loop regions with lower quality scores as compared to the model’s global average were refined via the MODELLER extension’s LoopModel protocol [43]. Global z-DOPE scoring and local DOPE scoring were measured after each iteration of selective minimization and loop refinement. Selective refinement was discontinued when it did not lower the model’s z-DOPE score by − 0.02 or more. Finally, global energy minimization of the model was performed to eliminate serious steric clashes. Following refinement and minimization, all models had z-DOPE scores − 1.28 ≥ − 1.55.

The HSYMDOCK server was then used to construct homo-oligomeric assemblies from the refined monomeric models [41]. The predicted homo-oligomeric unit for all modelled ALTs was a homotetramer with dihedral symmetry, similarly to other Type 9 single hot dog-fold bacterial thioesterases with solved crystal structures, including the *E. coli* acyl-ACP thioesterase YbgC (accession no. 5 T06 on the RCSB PDB) [35, 45]. For each ALT, the structure with the lowest free energy score out of 50 homotetrameric structures generated by HSYMDOCK was used for further analysis. The top homotetrameric models for each ALT consistently had docking scores < − 700, indicative of favourable interactions at binding interfaces [41]. High-energy residues in the ALT tetramer models were then corrected using the FoldX 5.0 suite’s RepairPDB command [54]. Repaired models were used for further inspection and visualization in UCSF ChimeraX 1.2.5, and for simulation of single- or multiple-residue mutations using the FoldX 5.0 suite’s BuildModel command [42, 54].

### In silico docking of *E. coli* acyl-ACPs with AtALT4

Interaction models of ALTs and *E. coli* C6-, C10-, and C14- acyl-ACPs (2FAC, 2FAE, and 3EJB on the RCSB PDB) were generated using the ClusPro docking server [49–53]. Distance restraints to guide docking were set based on prior knowledge of the interaction of acyl-ACPs with thioesterase proteins. The conserved S36 of ACP must be oriented towards a conserved catalytic Asp residue on the thioesterase’s active loop, which is D14 of the truncated hot dog-fold domain sequence of ALT-like thioesterases [20, 33, 34]. To accommodate the phosphopantetheine arm that connects the acyl chain with S36 of ACP, S36 was specified to be within 13.5 Å of D14 on the ALT’s active site loop [33]. ClusPro generated 10,000 models for each input pairing, which were clustered into the top 10 maximum likelihood conformations. The top 10 binding conformations for each ALT-acyl ACP input pair were then inspected in Chimera software to ensure satisfaction of distance restraints. The top-scoring binding conformation that appeared among all input pairs was taken as the representative ALT-acyl ACP interaction model.

## Supplementary Information

**Additional file 1: File S1.** Alignment of the complete protein sequences of ALT-type thioesterases from 11 plant species. The red line indicates the end of the predicted plastid targeting sequence and the start of the hot-dog fold thioesterase domain. Sequences were aligned using ClustalW [43]. Identical residues are highlighted in black, while chemically similar residues are highlighted in grey. *At = Arabidopsis thaliana, Bd = Brachypodium distachyon, Cs = Cannabis sativa, Cr = Chlamydomonas reinhardtii, Gm = Glycine max, Mt = Medicago truncatula, Os = Oryza sativa* subsp. *japonica, Sh = Solanum habrochaites* susbsp. *Glabratum, Sm = Solanum melongena, Vv = Vitis vinifera, Zm = Zea mays*.

**Additional file 2: Fig. S1.** Predicted catalytic and acyl-binding cavity residues in modelled ALTs align with those of *E. coli* YbgC and *Umbellularia californica* FatB [45, 46, 48]. **A.** Monomer models of the hot-dog fold domains of *Mt*ALT1 and the *E. coli* acyl-CoA thioesterase YbgC, with numbered α-helices and β-strands. ALT monomers were modelled by AlphaFold 2.0, and the crystal structure of *E. coli* YbgC was retrieved from the RCSB PDB (PDB ID: 5 T06) [39, 40, 45]. **B.** Superimposition of *Arabidopsis thaliana* ALT3/4*, Medicago truncatula* ALT1/2*,* and *Zea mays* ALT1/3 homotetramer models assembled with HSYMDOCK (grey) with the crystal structure of the *E. coli* acyl-CoA thioesterase YbgC (yellow) [39–41, 45]. **C.** Comparison of predicted catalytic residues in modelled ALTs and the crystal structure of *Ec*YbgC. *At*ALT4 is used as a representative example. Catalytic triad residues belong to two neighbouring subunits. **D.** Comparison of predicted acyl-binding cavity structure in modelled ALTs, and the crystal structures of *Ec*YbgC and *Uc*FatB. *Mt*ALT1 is used as a representative example. Ribbon structure of α1-α2 of the hot-dog fold domain is hidden from models to increase visibility of key residues. The crystal structure of *Uc*FatB was retrieved from the RCSB PDB (PDB ID: 5X04), and the N-terminal hot-dog fold domain (residues 100–247) were isolated in ChimeraX 1.2.5 software. Top: Predicted substrate-binding cavity residues of *Mt*ALT1 and *Ec*YbgC are shown as stick models. Middle: Molecular surfaces formed by predicted acyl binding cavity residues are coloured according to hydrophobicity (yellow = hydrophobic, white = amphipathic, blue = hydrophilic). Bottom: Predicted substrate-binding cavity residues of *Mt*ALT1, and experimentally determined substrate-binding cavity residues of *Uc*FatB are shown as stick models [48]. **E.** Alignment of the *Mt*ALT1, *Ec*YbgC, and N-terminal *Uc*FatB hot-dog fold domain sequences [55]. Acyl-binding cavity residues belonging to each protein are highlighted in red [46]. Asterisks (*) indicate predicted acyl-binding cavity-forming residues of *Mt*ALT1 that align with those of both *Ec*YbgC and *Uc*FatB. Dots (●) indicate cavity-forming residues of *Mt*ALT1 that align with those of *Ec*YbgC, but not *Uc*FatB. Diamonds (◊) indicate cavity-forming residues of *Uc*FatB that align structurally, but not on the sequence alignment, with those of *Mt*ALT1.

**Additional file 3: File S2.** Insert sequences of genetic constructs for wild-type and chimeric ALT expression in K27(DE3) *E. coli*.

**Additional file 4: Table S1.** PCR primers used to assemble select plasmid constructs encoding chimeric ALTs.

**Additional file 5: Fig. S2.** GC-FID chromatograms of secreted lipids from K27(DE3) *E. coli* cultures expressing wild-type and chimeric ALTs. β-keto fatty acids secreted into culture media were chemically decarboxylated to methylketones prior to GC-FID analysis. Fatty acids and methylketones were identified via GC-MS and by comparison to authentic retention standards. Unidentified peaks that consistently appeared in sample replicates are labelled as “UK” (unknown).

**Additional file 6: Fig. S3.** EI mass spectra of compounds detected in media from K27(DE3) *E. coli* cultures expressing ALTs. β-keto fatty acids secreted into culture media were chemically decarboxylated to methylketones prior to GC-MS analysis. Standard spectra used for compound identification are shown to the right of sample spectra. Fully reduced free fatty acids and saturated methylketones were identified by comparison to standard spectra from the NIST 17 Mass Spectral Library. Monounsaturated methylketones were identified by comparison to spectra previously obtained by Goh et al. [30]. 3-hydroxy fatty acids were identified based on the presence of their trimethylsilyl (TMS) derivatives in silylated samples. Mass spectra of the 2TMS derivatives of 3-hydroxy fatty acids were compared to standard spectra in the NIST 17 library, with the exception of 7(Z)-3-hydroxyhexadec-7-enoic acid, which is unlisted. The 2TMS derivative of 7(Z)-3-hydroxyhexadec-7-enoic acid was identified based the presence of the molecular ion (m/z = 386) and prominent ion fragments characteristic of both the 2TMS derivative of a 3-hydroxy fatty acid (m/z = 73, m/z = 147, m/z = 233, M^+^ − 31) and the TMS derivative of a monounsaturated fatty acid (M^+^ − 169, M^+^ − 90). Indole, a quorum sensing molecule, was present in media from all induced cultures.

**Additional file 7: Table S2.** Quantification and statistical analysis of fatty acid and methylketone production by K27(DE3) *E. coli* expressing wild-type or chimeric ALTs, from raw GC-FID data of treated culture media samples.

**Additional file 8: Fig. S4.** Full-length membranes used for immunodetection of ALT proteins following transfer from Tris-Tricine SDS-PAGE gels and staining with Ponceau S, and following immunodetection with anti-T7 mouse monoclonal primary antibody and an anti-mouse horseradish peroxidase-conjugated secondary antibody. Ponceau-stained membranes were imaged under white light with an exposure time of 1/30s. Probed membranes were imaged at 59 s exposure using a BioRad ChemiDoc XRS+ system with ImageLab v6.0.1 software. Black rectangles delineate where Ponceau-stained membranes were cut prior to being probed with antibody, and the boundaries of probed membranes. Red rectangles indicate regions where membranes were cropped to construct Fig. 5. Graphs relating ALT protein accumulation to total FA + MK productivity in *E. coli* are shown to the right of each membrane. Total FA + MK productivity of ALTs, in units of nmol / mL OD_600_ is represented by grey bars, with bar height corresponding to values on the left-hand vertical axis. Dots (●) indicate relative expression levels of the heterologously expressed ALT proteins, with values on the right-hand vertical axis. Thioesterase productivity values reported are the average of triplicate samples, with error representing ± SE (data shown in Table S2). Relative protein expression levels were calculated by normalizing ALT band volume (intensity) on antibody-probed membranes to total lane volume on Ponceau S-stained membranes in ImageLab v6.0.1 software. Unlabelled lanes represent *E. coli* strains expressing ALT constructs that were not analyzed further in this work.

**Additional file 9: Fig. S5.** Predicted per-residue confidence levels of the catalytic domain structures of ALTs from *Arabidopsis thaliana*, *Medicago truncatula,* and *Zea mays,* modelled by AlphaFold 2.0 [39, 40].

## Data Availability

The dataset supporting the conclusions of this article is included within the article and its additional files. Protein models are available upon request.

## Abbreviations

3-OH: 3-hydroxy
aa: amino acid
ACP: Acyl carrier protein
ALT: Acyl lipid thioesterase
BSA: Bovine serum albumin
FA: Fatty acid
Fat: Fatty acyl thioesterase
MCFA: Medium-chain fatty acid
MK: Methylketone
MKS: Methylketone synthase
TMS: Trimethylsilyl
