# Supplementary figures and images for: Determinants of substrate specificity in a catalytically diverse family of acyl-ACP thioesterases from plants
Source: BMC Plant Biol. 2023 Jan 2;23:1. doi: 10.1186/s12870-022-04003-y (PMC9806908; doi:10.1186/s12870-022-04003-y)

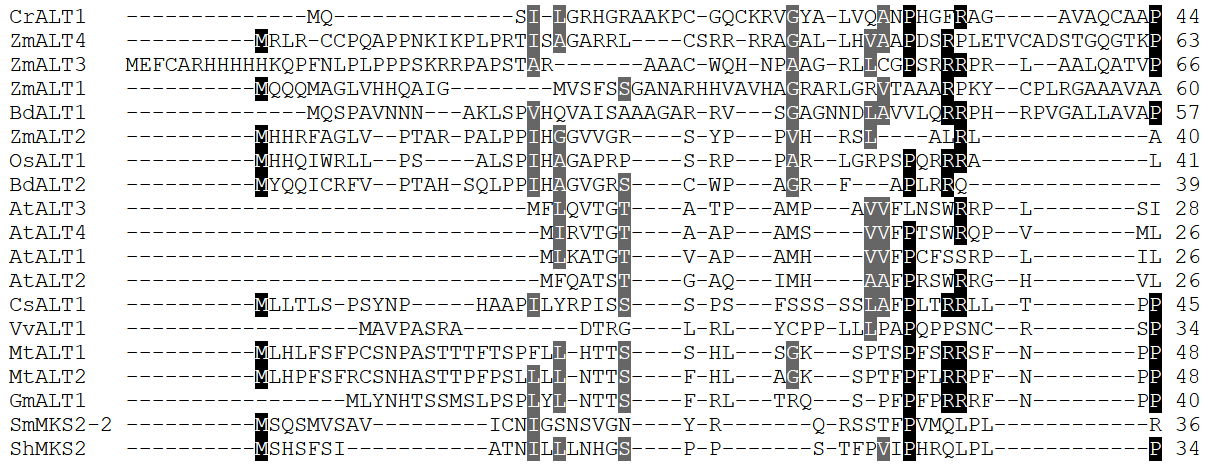


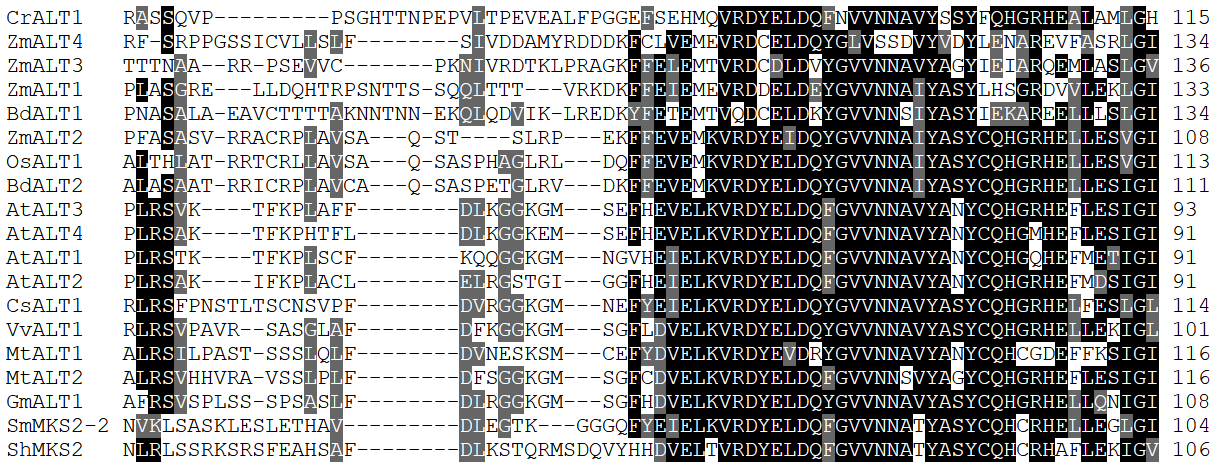


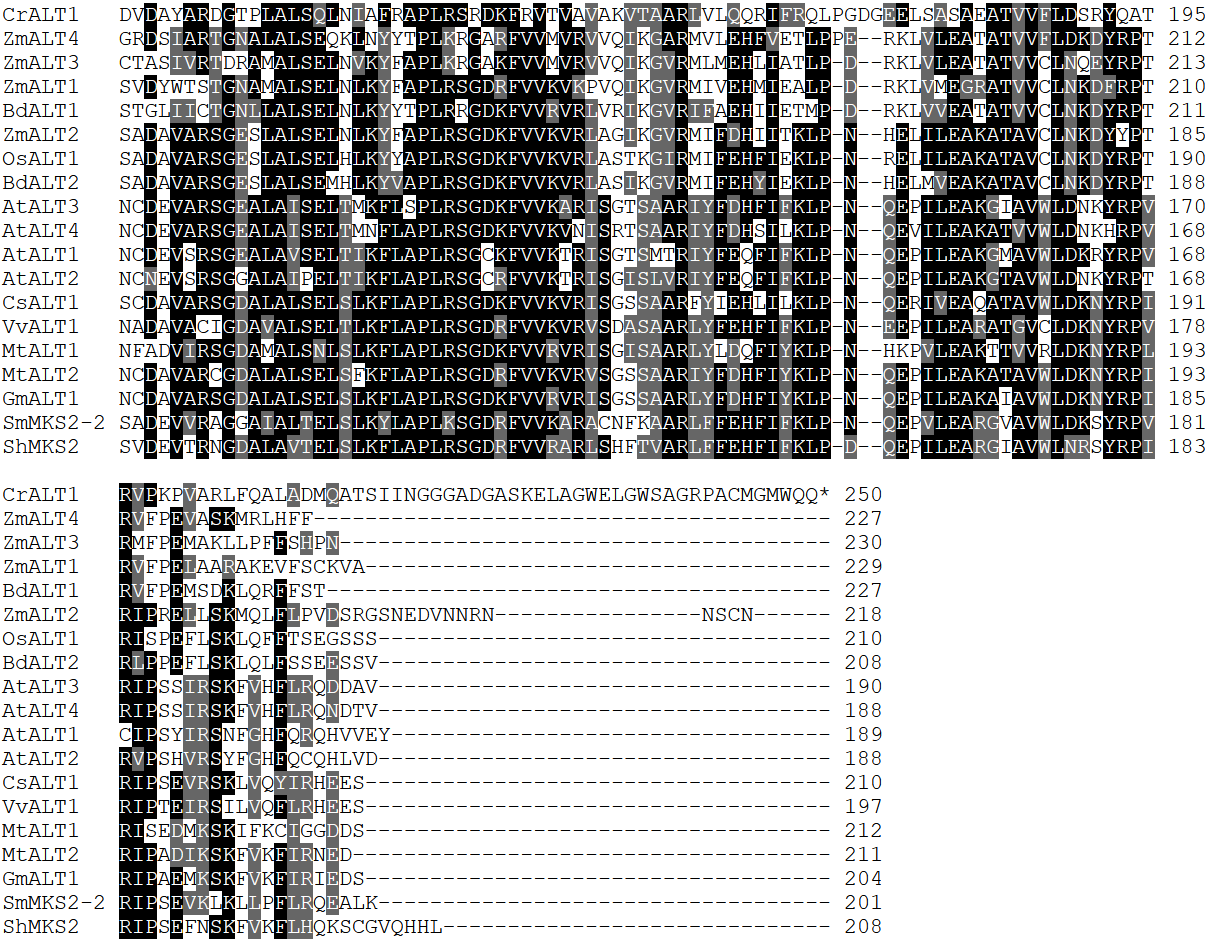

Supplement: Supplementary file 1 — Additional file 1: File S1. Alignment of the complete protein sequences of ALT-type thioesterases from 11 plant species. The red line indicates the end of the predicted plastid targeting sequence and the start of the hot-dog fold thioesterase domain. Sequences were aligned using ClustalW [43]. Identical residues are highlighted in black, while chemically similar residues are highlighted in grey. At = Arabidopsis thaliana, Bd = Brachypodium distachyon, Cs = Cannabis sativa, Cr = Chlamydomonas reinhardtii, Gm = Glycine max, Mt = Medicago truncatula, Os = Oryza sativa subsp. japonica, Sh = Solanum habrochaites susbsp. Glabratum, Sm = Solanum melongena, Vv = Vitis vinifera, Zm = Zea mays. [file 12870_2022_4003_MOESM1_ESM.docx]

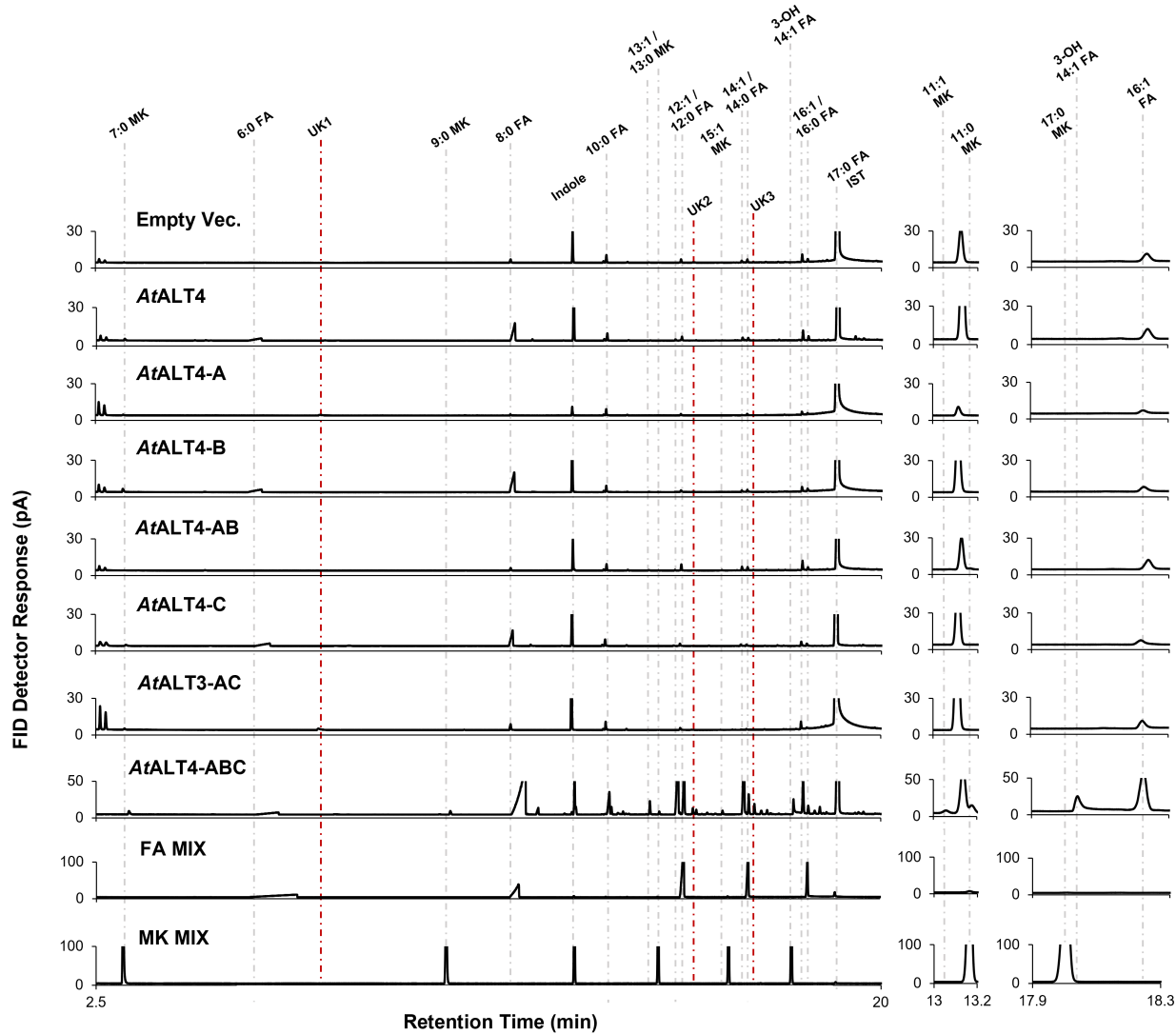

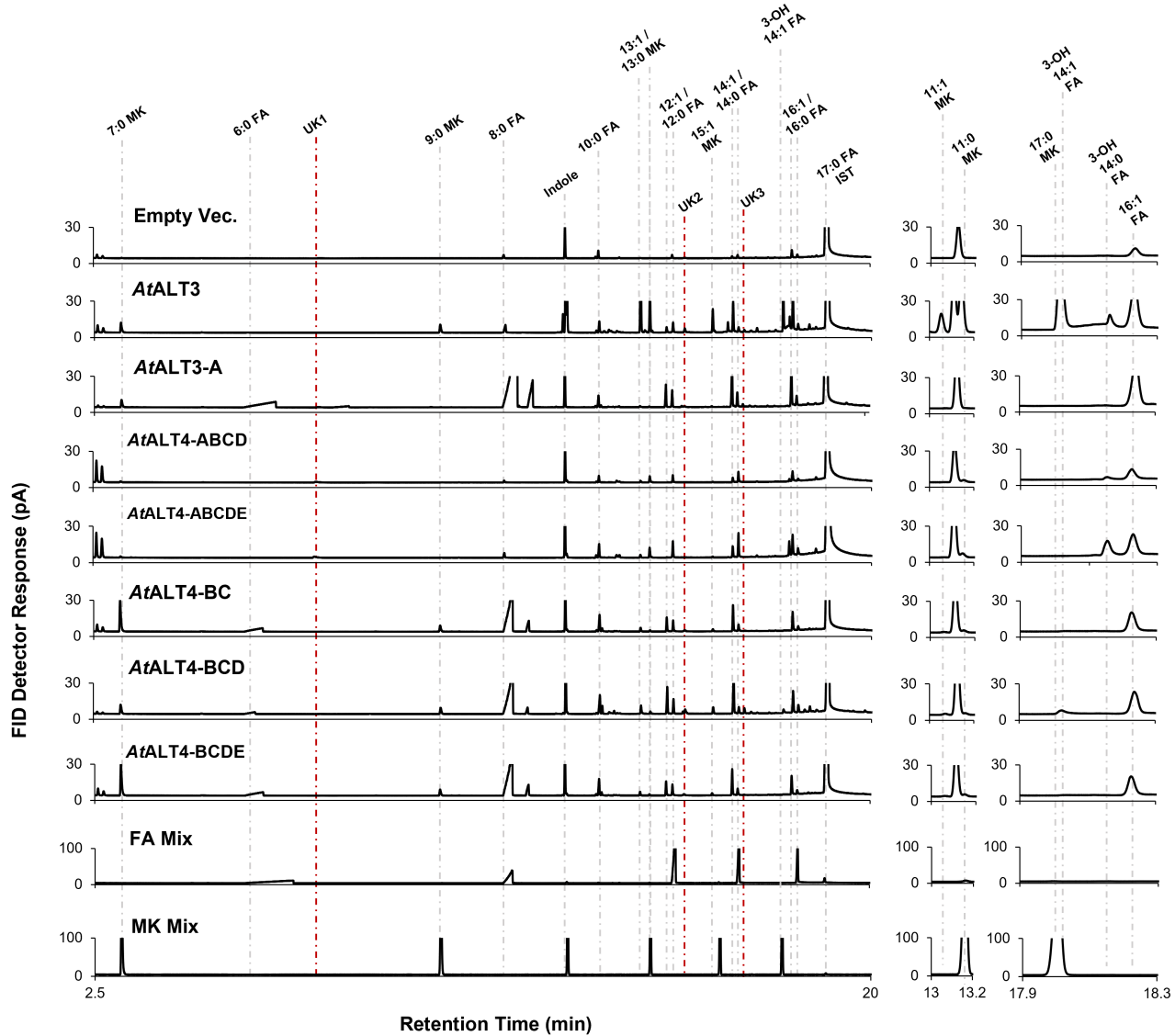

FID Detector Response (pA)

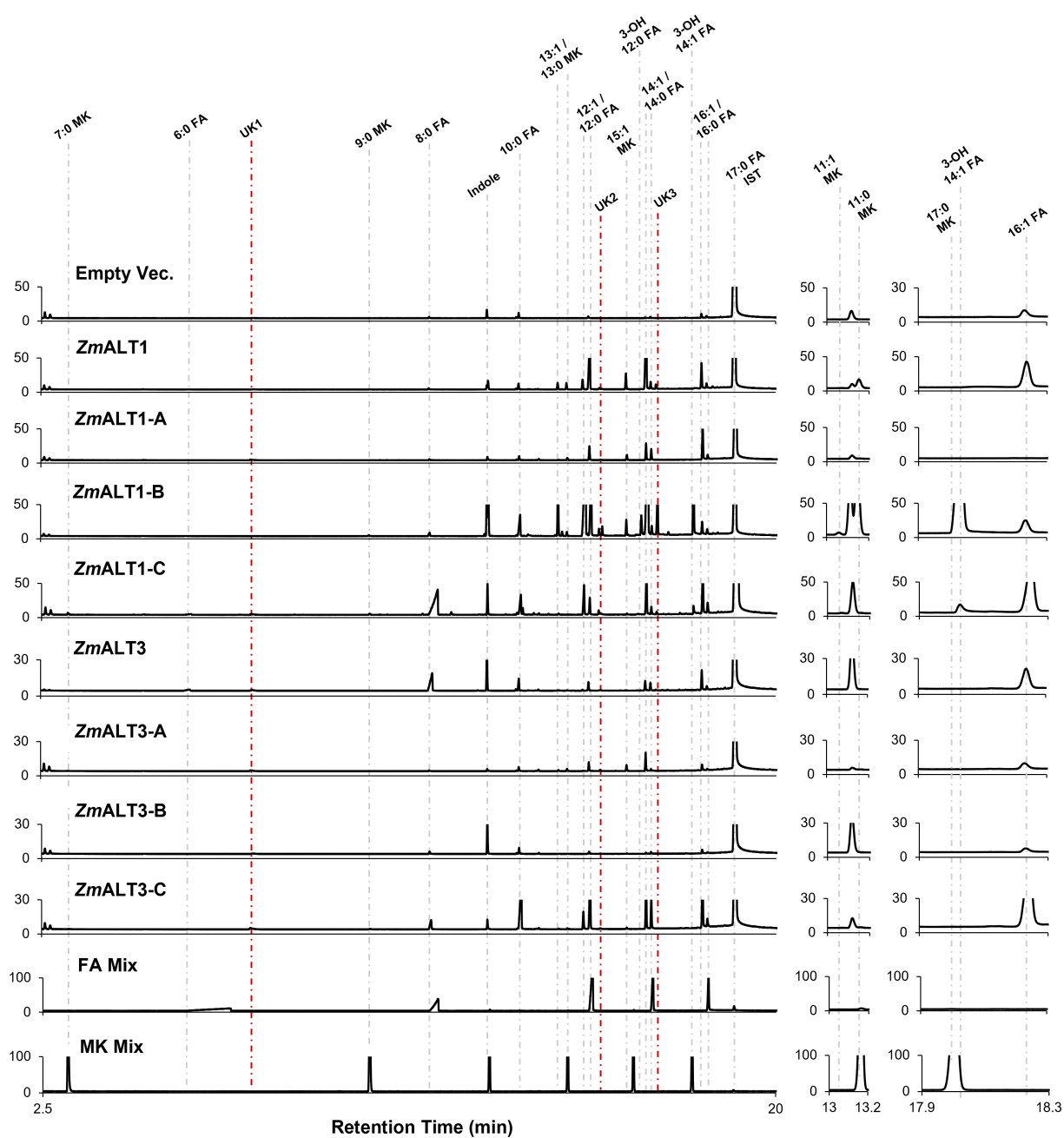

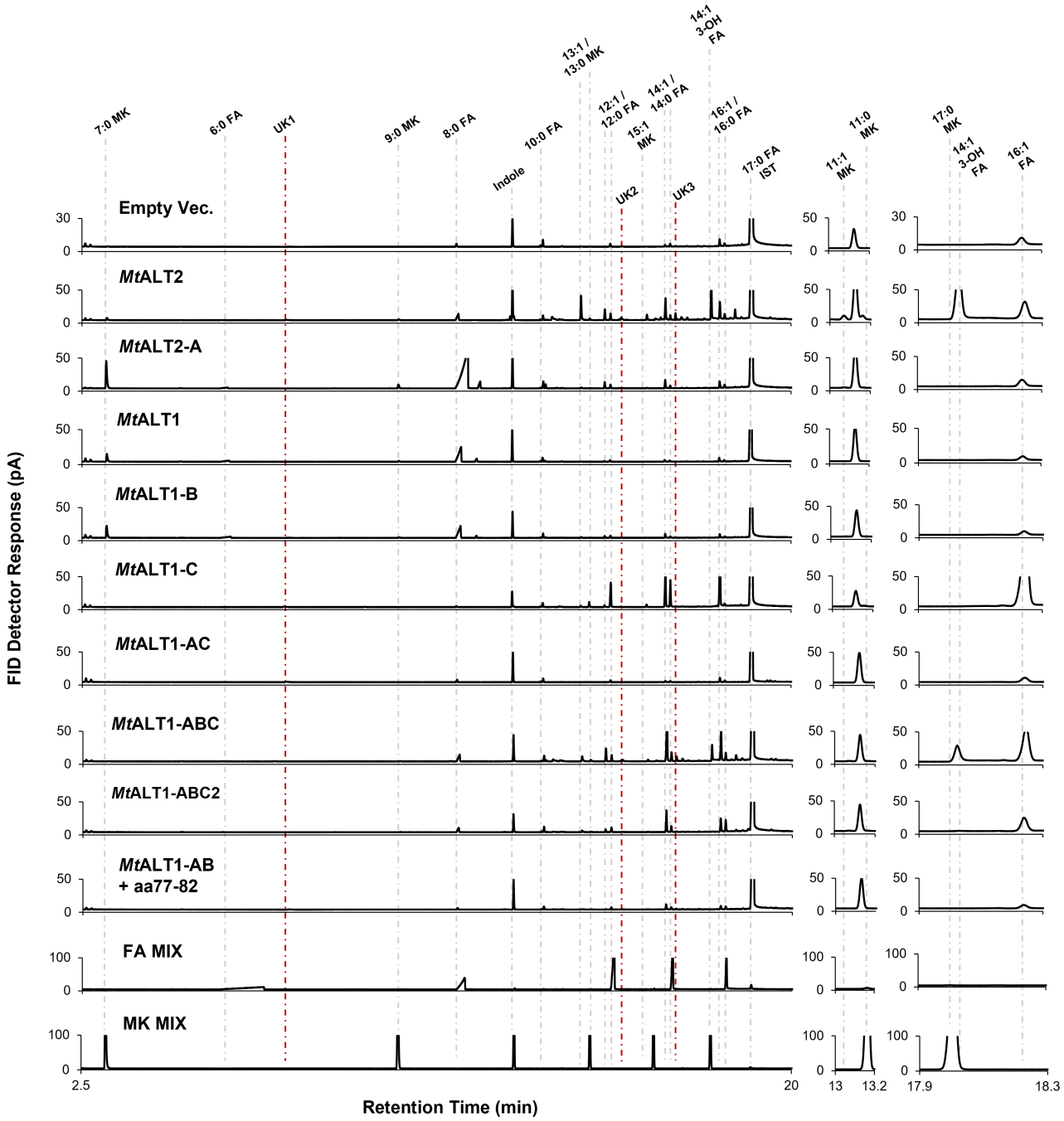

Supplement: Supplementary file 5 — Additional file 5: Fig. S2. GC-FID chromatograms of secreted lipids from K27(DE3) E. coli cultures expressing wild-type and chimeric ALTs. β-keto fatty acids secreted into culture media were chemically decarboxylated to methylketones prior to GC-FID analysis. Fatty acids and methylketones were identified via GC-MS and by comparison to authentic retention standards. Unidentified peaks that consistently appeared in sample replicates are labelled as “UK” (unknown). [file 12870_2022_4003_MOESM5_ESM.pdf]

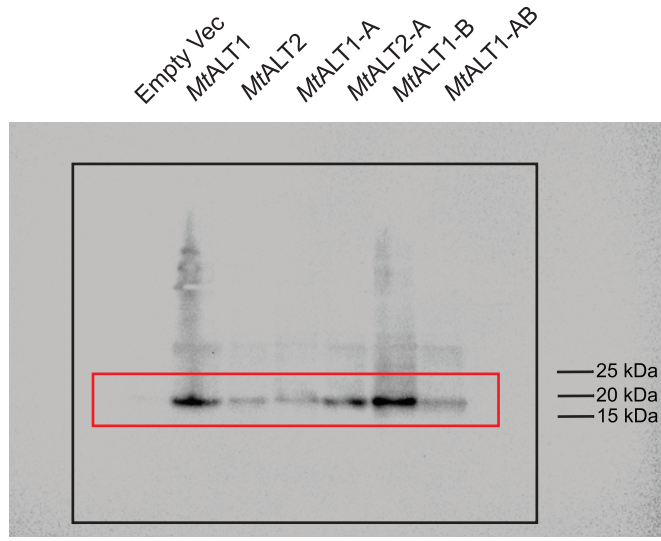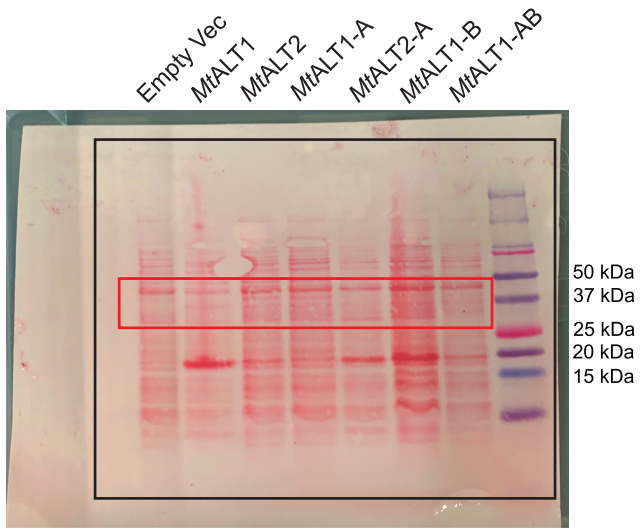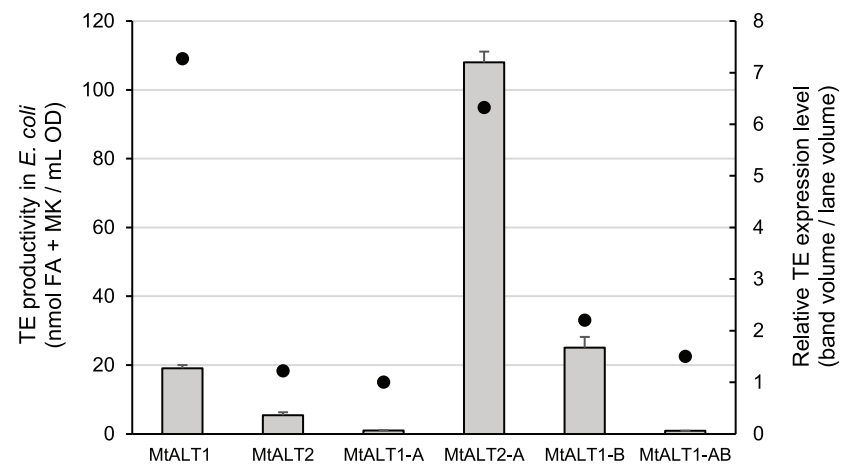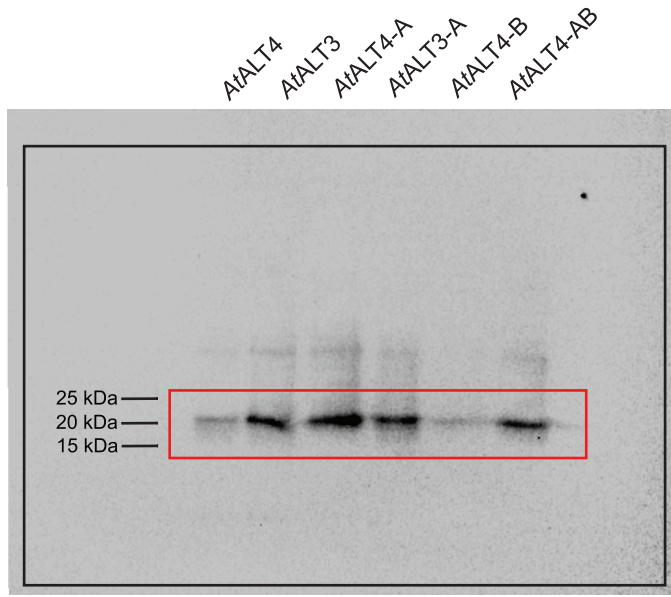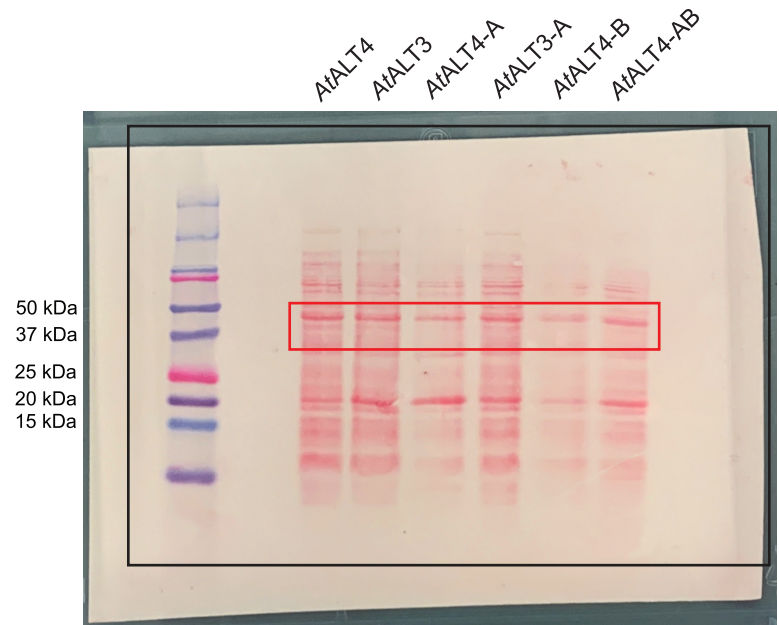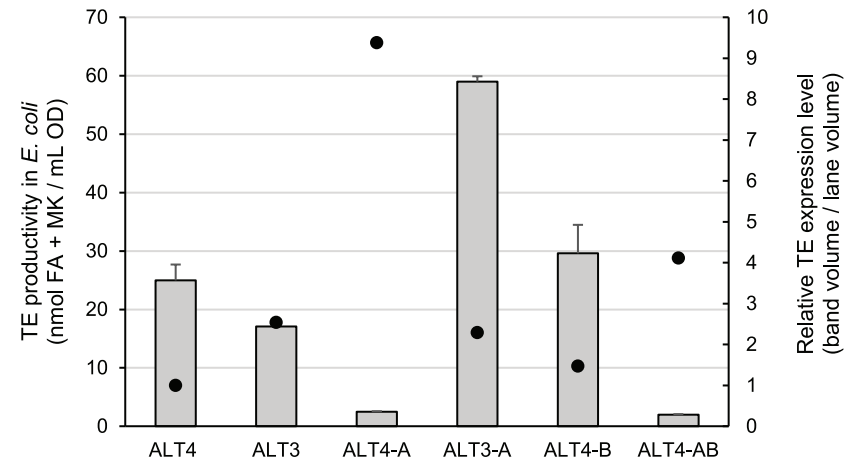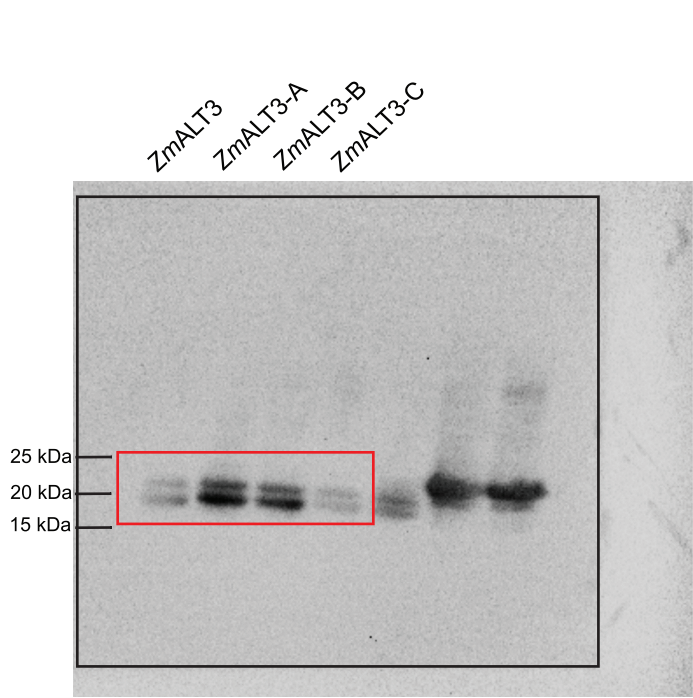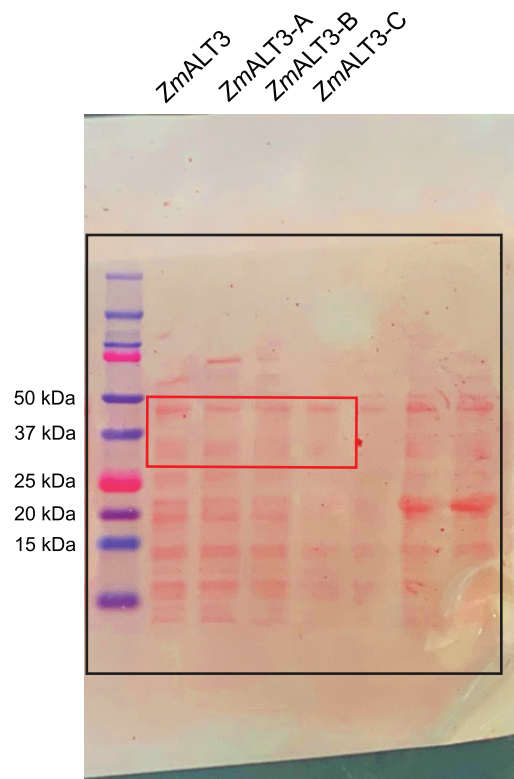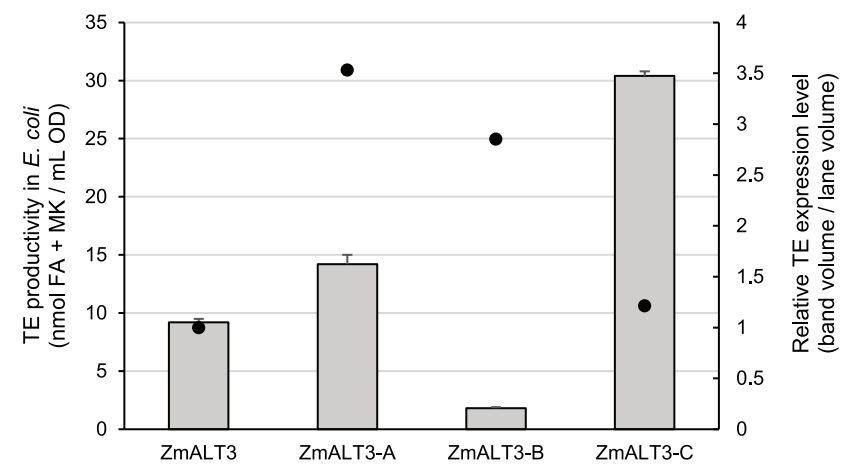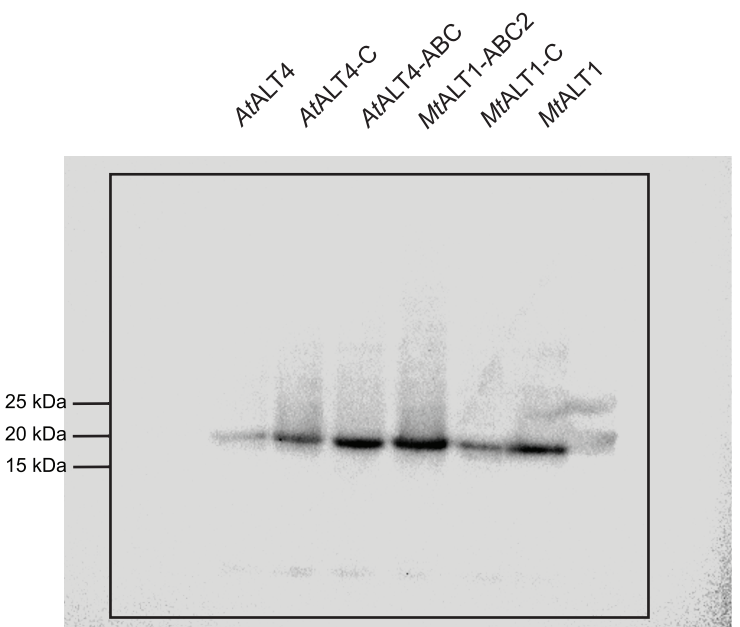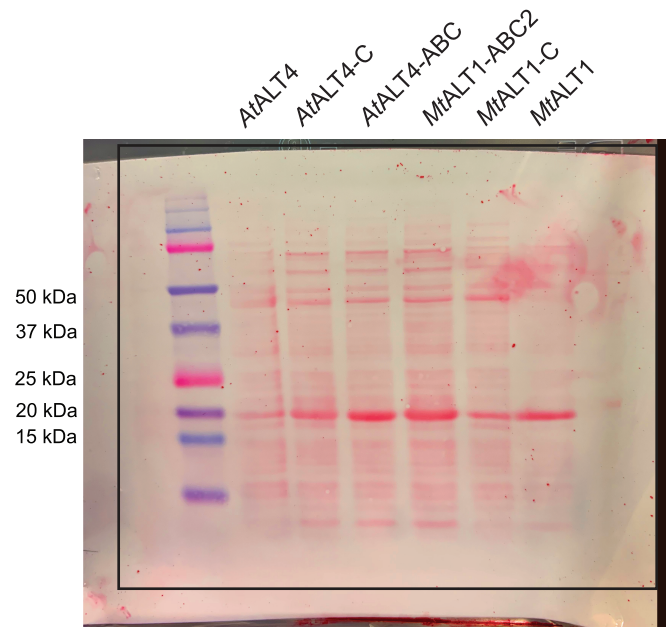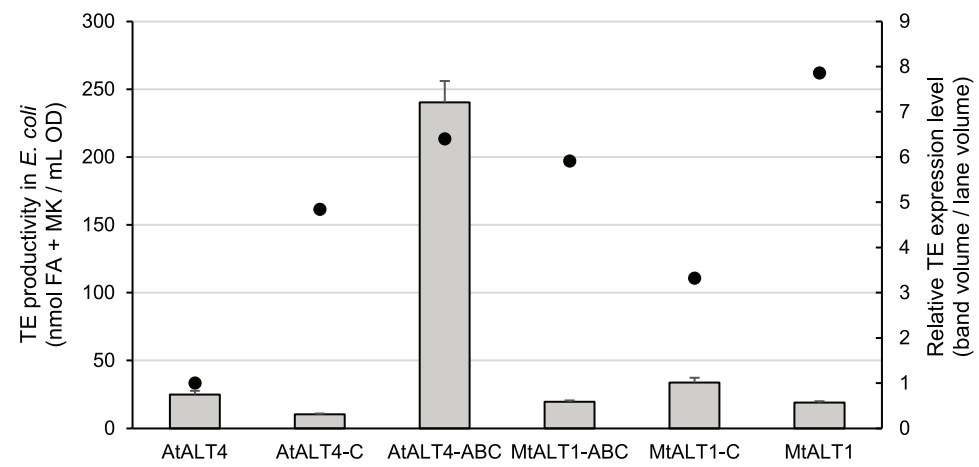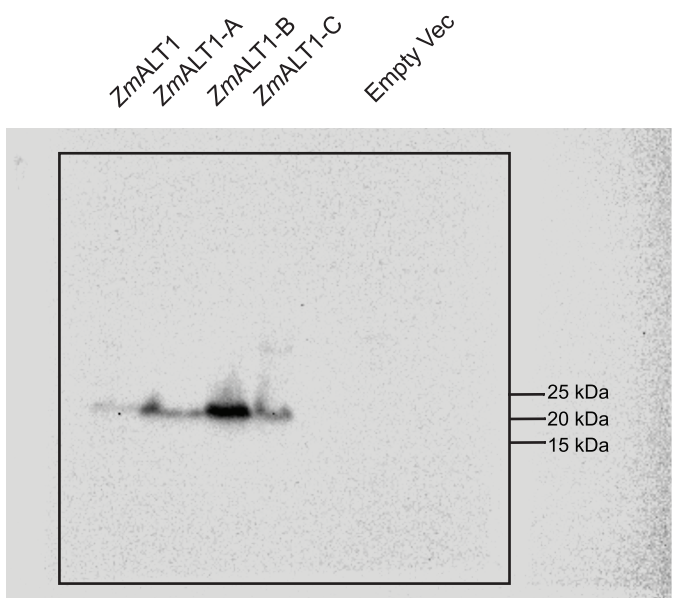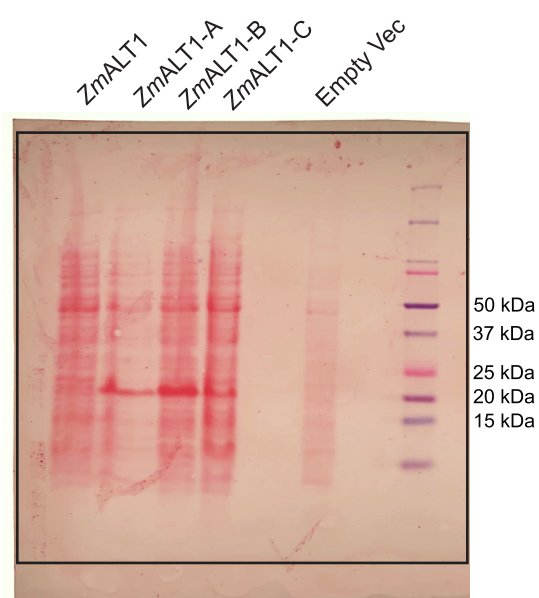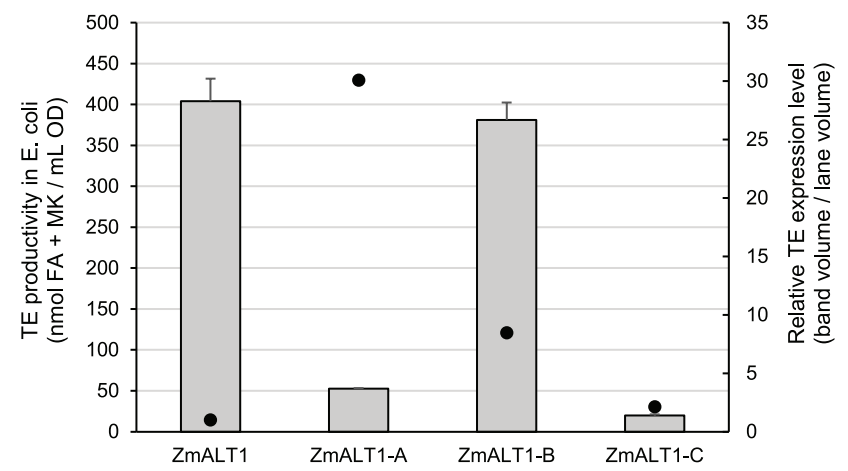

Supplement: Supplementary file 8 — Additional file 8: Fig. S4. Full-length membranes used for immunodetection of ALT proteins following transfer from Tris-Tricine SDS-PAGE gels and staining with Ponceau S, and following immunodetection with anti-T7 mouse monoclonal primary antibody and an anti-mouse horseradish peroxidase-conjugated secondary antibody. Ponceau-stained membranes were imaged under white light with an exposure time of 1/30s. Probed membranes were imaged at 59 s exposure using a BioRad ChemiDoc XRS+ system with ImageLab v6.0.1 software. Black rectangles delineate where Ponceau-stained membranes were cut prior to being probed with antibody, and the boundaries of probed membranes. Red rectangles indicate regions where membranes were cropped to construct Fig. 5. Graphs relating ALT protein accumulation to total FA + MK productivity in E. coli are shown to the right of each membrane. Total FA + MK productivity of ALTs, in units of nmol / mL OD600 is represented by grey bars, with bar height corresponding to values on the left-hand vertical axis. Dots (●) indicate relative expression levels of the heterologously expressed ALT proteins, with values on the right-hand vertical axis. Thioesterase productivity values reported are the average of triplicate samples, with error representing ± SE (data shown in Table S2). Relative protein expression levels were calculated by normalizing ALT band volume (intensity) on antibody-probed membranes to total lane volume on Ponceau S-stained membranes in ImageLab v6.0.1 software. Unlabelled lanes represent E. coli strains expressing ALT constructs that were not analyzed further in this work. [file 12870_2022_4003_MOESM8_ESM.pdf]

*AtALT3*

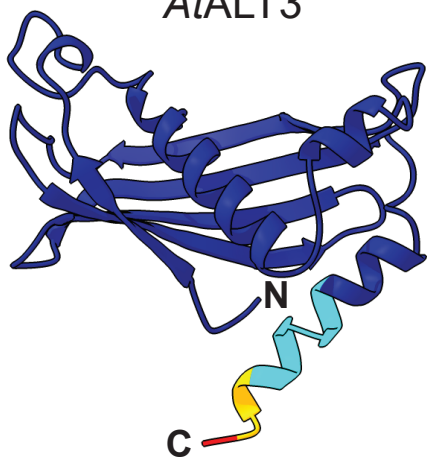

*AtALT4*

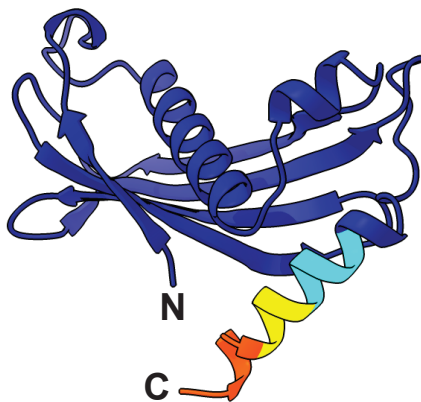

*MtALT1*

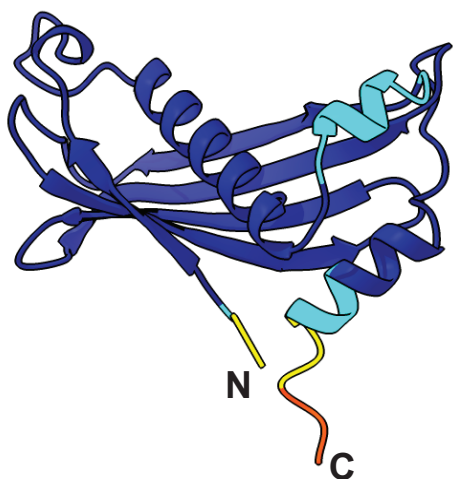

*MtALT2*

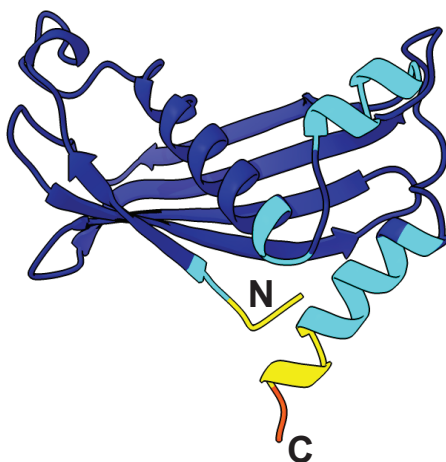

Per-residue modelling  
confidence (pLDDT)

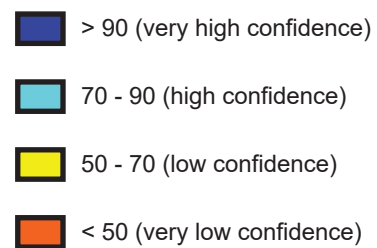

*ZmALT1*

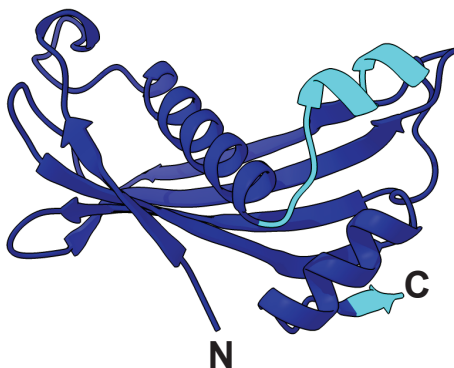

*ZmALT3*

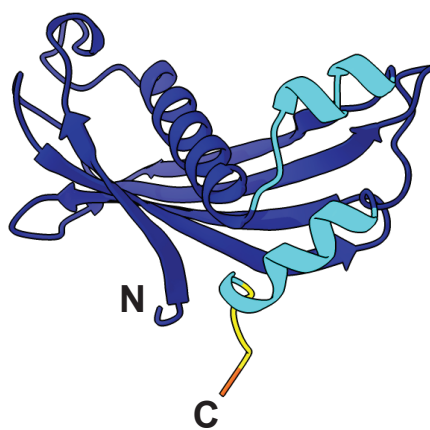

Supplement: Supplementary file 9 — Additional file 9: Fig. S5. Predicted per-residue confidence levels of the catalytic domain structures of ALTs from Arabidopsis thaliana, Medicago truncatula, and Zea mays, modelled by AlphaFold 2.0 [39, 40]. [file 12870_2022_4003_MOESM9_ESM.pdf]
