# Supplementary material for: Determinants of substrate specificity in a catalytically diverse family of acyl-ACP thioesterases from plants
Source: BMC Plant Biol. 2023 Jan 2;23:1. doi: 10.1186/s12870-022-04003-y (PMC9806908; doi:10.1186/s12870-022-04003-y)
Supplement: Supplementary file 2 — Additional file 2: Fig. S1. Predicted catalytic and acyl-binding cavity residues in modelled ALTs align with those of E. coli YbgC and Umbellularia californica FatB [45, 46, 48]. A. Monomer models of the hot-dog fold domains of MtALT1 and the E. coli acyl-CoA thioesterase YbgC, with numbered α-helices and β-strands. ALT monomers were modelled by AlphaFold 2.0, and the crystal structure of E. coli YbgC was retrieved from the RCSB PDB (PDB ID: 5 T06) [39, 40, 45]. B. Superimposition of Arabidopsis thaliana ALT3/4, Medicago truncatula ALT1/2, and Zea mays ALT1/3 homotetramer models assembled with HSYMDOCK (grey) with the crystal structure of the E. coli acyl-CoA thioesterase YbgC (yellow) [39–41, 45]. C. Comparison of predicted catalytic residues in modelled ALTs and the crystal structure of EcYbgC. AtALT4 is used as a representative example. Catalytic triad residues belong to two neighbouring subunits. D. Comparison of predicted acyl-binding cavity structure in modelled ALTs, and the crystal structures of EcYbgC and UcFatB. MtALT1 is used as a representative example. Ribbon structure of α1-α2 of the hot-dog fold domain is hidden from models to increase visibility of key residues. The crystal structure of UcFatB was retrieved from the RCSB PDB (PDB ID: 5X04), and the N-terminal hot-dog fold domain (residues 100–247) were isolated in ChimeraX 1.2.5 software. Top: Predicted substrate-binding cavity residues of MtALT1 and EcYbgC are shown as stick models. Middle: Molecular surfaces formed by predicted acyl binding cavity residues are coloured according to hydrophobicity (yellow = hydrophobic, white = amphipathic, blue = hydrophilic). Bottom: Predicted substrate-binding cavity residues of MtALT1, and experimentally determined substrate-binding cavity residues of UcFatB are shown as stick models [48]. E. Alignment of the MtALT1, EcYbgC, and N-terminal UcFatB hot-dog fold domain sequences [55]. Acyl-binding cavity residues belonging to each protein are highlighted in red [46 [file 12870_2022_4003_MOESM2_ESM.pdf]

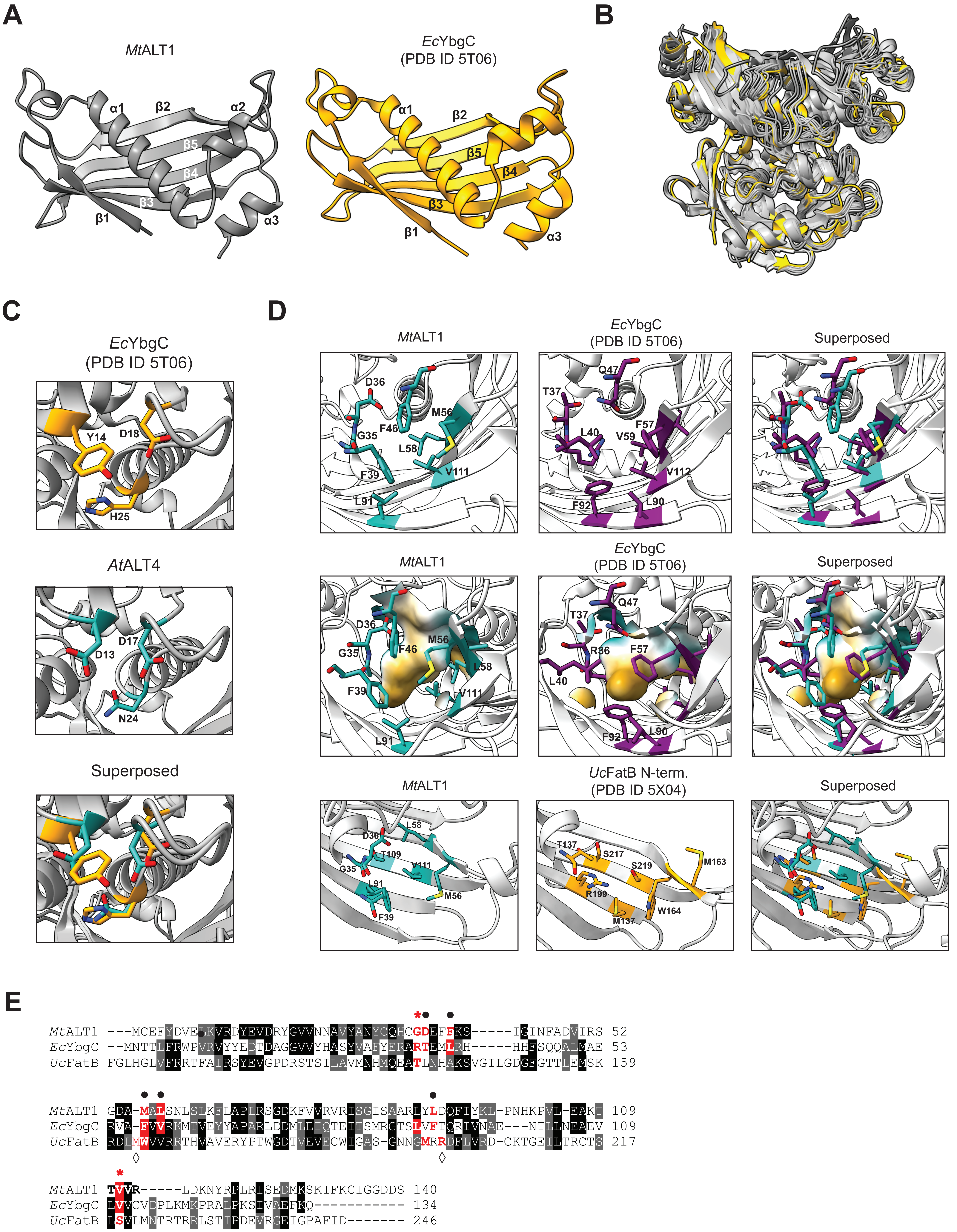
A
MtALT1
α1
β2
α2
β5
β4
β3
β1
α3
EcYbgC
(PDB ID 5T06)
B
C
EcYbgC
(PDB ID 5T06)
Y14
D18
H25
AtALT4
D17
D13
N24
Superposed
D
MtALT1
D36
M56
F46
G35
L58
F39
V111
L91
EcYbgC
(PDB ID 5T06)
Q47
T37
L40
F57
V59
V112
F92
L90
Superposed
MtALT1
D36
M56
G35
F46
L58
F39
V111
L91
EcYbgC
(PDB ID 5T06)
Q47
T37
R36
F57
L40
F92
L90
Superposed
MtALT1
L58
D36
T109
G35
V111
L91
M56
F39
UcFatB N-term.
(PDB ID 5X04)
T137
S217
S219
M163
R199
W164
M137
Superposed
E
MtALT1 ---MCEFYDVELKVRDYEVDRYGVVNNAVYANYCQHCGDEFFKS-----IGINFADVIRS 52
EcYbgC --MNTTLFRWPVRVYYEDTDAGGVVYHASYVAFYERARTEMLRH-----HHFSQQALMAE 53
UcFatB FGLHGLVFRRTFAIRSYEVGPDRSTSILAVMNHMQEATLNHAKSVGILGDGFGTTLEMSK 159
MtALT1 GDA-MALSNLSLKFLAPLRSGDKFVVRVRISGISAARLYLDQFIYKL-PNHKPVL-EAKT 109
EcYbgC RVA-FVVRKMTVEYYAPARLDDMLEIQTEITSMRGTSLVFTQRIVNAE---NTLLNEAEV 109
UcFatB RDLMWVVRRTHVAVERYPTWGDTVEVECWIGAS-GNNGMRRDFLVRD-CKTGEILTRCTS 217
MtALT1 TVVR-----LDKNYRPLRISEDMKSKIFKCIGGDDS 140
EcYbgC LVVCVDPLKMKPRALPKSIVAEFKQ----------- 134
UcFatB LSVLMNTRTRRLSTIPDEVRGEIGPAFID------- 246
