# Supplementary material for: Determinants of substrate specificity in a catalytically diverse family of acyl-ACP thioesterases from plants
Source: BMC Plant Biol. 2023 Jan 2;23:1. doi: 10.1186/s12870-022-04003-y (PMC9806908; doi:10.1186/s12870-022-04003-y)
Supplement: Supplementary file 3 — Additional file 3: File S2. Insert sequences of genetic constructs for wild-type and chimeric ALT expression in K27(DE3) E. coli. [file 12870_2022_4003_MOESM3_ESM.docx]

**Additional file 3: File S2.** Insert sequences of genetic constructs for wild-type and chimeric ALT expression in K27(DE3) *E. coli.*

*At*ALT3

AATGGGTCGCGGATCCATGAGTGAGTTCCATGAGGTTGAACTCAAAGTTCGTGATTATGAATTGGATCAGTTTGGTGTTGTGAACAATGCTGTTTACGCAAACTACTGTCAACACGGTCGACATGAGTTTCTAGAGAGTATCGGTATCAACTGCGACGAAGTAGCACGTTCTGGGGAAGCCTTAGCAATTTCAGAGTTGACAATGAAGTTCCTTTCACCTTTACGTAGCGGAGACAAATTCGTGGTGAAAGCGAGGATATCGGGGACATCTGCTGCGCGTATTTACTTCGATCATTTCATCTTTAAACTTCCAAATCAAGAGCCTATATTGGAGGCAAAAGGAATAGCTGTGTGGCTCGACAACAAGTACCGTCCTGTTCGCATCCCATCTTCTATACGTTCTAAATTTGTTCACTTCCTACGCCAAGACGACGCCGTTTGAAAGCTTGCGGCCGCAC

*At*ALT4

AATGGGTCGCGGATCCATGAGTGAGTTCCATGAGGTTGAGCTTAAAGTTCGTGATTATGAATTGGATCAGTTTGGTGTTGTGAACAATGCTGTTTACGCAAACTACTGCCAACACGGCATGCACGAATTTCTAGAGAGTATTGGTATCAACTGTGATGAAGTTGCCCGTTCTGGTGAAGCCTTAGCAATATCAGAGTTGACAATGAATTTCCTTGCACCTTTACGTAGCGGAGACAAGTTTGTAGTGAAAGTGAACATATCTAGAACATCTGCTGCGCGTATTTACTTCGATCATTCCATCTTGAAACTTCCAAATCAAGAGGTTATATTGGAGGCGAAAGCAACAGTTGTATGGCTTGACAACAAGCACCGTCCTGTTCGTATCCCATCTTCGATACGCTCTAAATTTGTTCACTTCCTACGCCAAAACGACACAGTTTGAAAGCTTGCGGCCGCAC

*At*ALT3-A

AATGGGTCGCGGATCCATGAGTGAGTTCCATGAGGTTGAACTCAAAGTTCGTGATTATGAATTGGATCAGTTTGGTGTTGTGAACAATGCTGTTTACGCAAACTACTGTCAACACGGT**ATG**CATGAGTTTCTAGAGAGTATCGGTATCAACTGCGACGAAGTAGCACGTTCTGGGGAAGCCTTAGCAATTTCAGAGTTGACAATGAAGTTCCTTTCACCTTTACGTAGCGGAGACAAATTCGTGGTGAAAGCGAGGATATCGGGGACATCTGCTGCGCGTATTTACTTCGATCATTTCATCTTTAAACTTCCAAATCAAGAGCCTATATTGGAGGCAAAAGGAATAGCTGTGTGGCTCGACAACAAGTACCGTCCTGTTCGCATCCCATCTTCTATACGTTCTAAATTTGTTCACTTCCTACGCCAAGACGACGCCGTTTGAAAGCTTGCGGCCGCAC

*At*ALT4-A

AATGGGTCGCGGATCCATGAGTGAGTTCCATGAGGTTGAGCTTAAAGTTCGTGATTATGAATTGGATCAGTTTGGTGTTGTGAACAATGCTGTTTACGCAAACTACTGCCAACACGGC**CGA**CACGAATTTCTAGAGAGTATTGGTATCAACTGTGATGAAGTTGCCCGTTCTGGTGAAGCCTTAGCAATATCAGAGTTGACAATGAATTTCCTTGCACCTTTACGTAGCGGAGACAAGTTTGTAGTGAAAGTGAACATATCTAGAACATCTGCTGCGCGTATTTACTTCGATCATTCCATCTTGAAACTTCCAAATCAAGAGGTTATATTGGAGGCGAAAGCAACAGTTGTATGGCTTGACAACAAGCACCGTCCTGTTCGTATCCCATCTTCGATACGCTCTAAATTTGTTCACTTCCTACGCCAAAACGACACAGTTTGAAAGCTTGCGGCCGCAC

*At*ALT4-B

AATGGGTCGCGGATCCATGAGTGAGTTCCATGAGGTTGAGCTTAAAGTTCGTGATTATGAATTGGATCAGTTTGGTGTTGTGAACAATGCTGTTTACGCAAACTACTGCCAACACGGCATGCACGAATTTCTAGAGAGTATTGGTATCAACTGTGATGAAGTTGCCCGTTCTGGTGAAGCCTTAGCAATATCAGAGTTGACAATGAATTTCCTTGCACCTTTACGTAGCGGAGACAAGTTTGTAGTGAAAGTGAACATATCTAGAACATCTGCTGCGCGTATTTACTTCGATCATTCCATCTTGAAACTTCCAAATCAAGAGGTTATATTGGAGGCGAAA**GGAATAGCT**GTATGGCTTGACAACAAGCACCGTCCTGTTCGTATCCCATCTTCGATACGCTCTAAATTTGTTCACTTCCTACGCCAAAACGACACAGTTTGAAAGCTTGCGGCCGCAC

*At*ALT4-AB

AATGGGTCGCGGATCCATGAGTGAGTTCCATGAGGTTGAGCTTAAAGTTCGTGATTATGAATTGGATCAGTTTGGTGTTGTGAACAATGCTGTTTACGCAAACTACTGCCAACACGGC**CGA**CACGAATTTCTAGAGAGTATTGGTATCAACTGTGATGAAGTTGCCCGTTCTGGTGAAGCCTTAGCAATATCAGAGTTGACAATGAATTTCCTTGCACCTTTACGTAGCGGAGACAAGTTTGTAGTGAAAGTGAACATATCTAGAACATCTGCTGCGCGTATTTACTTCGATCATTCCATCTTGAAACTTCCAAATCAAGAGGTTATATTGGAGGCGAAA**GGAATAGCT**GTATGGCTTGACAACAAGCACCGTCCTGTTCGTATCCCATCTTCGATACGCTCTAAATTTGTTCACTTCCTACGCCAAAACGACACAGTTTGAAAGCTTGCGGCCGCAC

*At*ALT4-C

AATGGGTCGCGGATCCATGAGTGAGTTCCATGAGGTTGAGCTTAAAGTTCGTGATTATGAATTGGATCAGTTTGGTGTTGTGAACAATGCTGTTTACGCAAACTACTGCCAACACGGCATGCACGAATTTCTAGAGAGTATTGGTATCAACTGTGATGAAGTTGCCCGTTCTGGTGAAGCCTTAGCAATATCAGAGTTGACAATGAATTTCCTTGCACCTTTACGTAGCGGAGACAAGTTTGTAGTGAAA**GCGAGGATATCGGGG**ACATCTGCTGCGCGTATTTACTTCGATCATTCCATCTTGAAACTTCCAAATCAAGAGGTTATATTGGAGGCGAAAGCAACAGTTGTATGGCTTGACAACAAGCACCGTCCTGTTCGTATCCCATCTTCGATACGCTCTAAATTTGTTCACTTCCTACGCCAAAACGACACAGTTTGAAAGCTTGCGGCCGCAC

*At*ALT4-AC

AATGGGTCGCGGATCCATGAGTGAGTTCCATGAGGTTGAGCTTAAAGTTCGTGATTATGAATTGGATCAGTTTGGTGTTGTGAACAATGCTGTTTACGCAAACTACTGCCAACACGGC**CGA**CACGAATTTCTAGAGAGTATTGGTATCAACTGTGATGAAGTTGCCCGTTCTGGTGAAGCCTTAGCAATATCAGAGTTGACAATGAATTTCCTTGCACCTTTACGTAGCGGAGACAAGTTTGTAGTGAAA**GCGAGGATATCGGGG**ACATCTGCTGCGCGTATTTACTTCGATCATTCCATCTTGAAACTTCCAAATCAAGAGGTTATATTGGAGGCGAAAGCAACAGTTGTATGGCTTGACAACAAGCACCGTCCTGTTCGTATCCCATCTTCGATACGCTCTAAATTTGTTCACTTCCTACGCCAAAACGACACAGTTTGAAAGCTTGCGGCCGCAC

*At*ALT4-ABC

AATGGGTCGCGGATCCATGAGTGAGTTCCATGAGGTTGAGCTTAAAGTTCGTGATTATGAATTGGATCAGTTTGGTGTTGTGAACAATGCTGTTTACGCAAACTACTGCCAACACGGC**CGA**CACGAATTTCTAGAGAGTATTGGTATCAACTGTGATGAAGTTGCCCGTTCTGGTGAAGCCTTAGCAATATCAGAGTTGACAATGAATTTCCTTGCACCTTTACGTAGCGGAGACAAGTTTGTAGTGAAA**GCGAGGATATCGGGG**ACATCTGCTGCGCGTATTTACTTCGATCATTCCATCTTGAAACTTCCAAATCAAGAGGTTATATTGGAGGCGAAA**GGAATAGCT**GTATGGCTTGACAACAAGCACCGTCCTGTTCGTATCCCATCTTCGATACGCTCTAAATTTGTTCACTTCCTACGCCAAAACGACACAGTTTGAAAGCTTGCGGCCGCAC

*At*ALT4-ABCD

AATGGGTCGCGGATCCATGAGTGAGTTCCATGAGGTTGAGCTTAAAGTTCGTGATTATGAATTGGATCAGTTTGGTGTTGTGAACAATGCTGTTTACGCAAACTACTGCCAACACGGC**CGA**CACGAATTTCTAGAGAGTATTGGTATCAACTGTGATGAAGTTGCCCGTTCTGGTGAAGCCTTAGCAATATCAGAGTTGACAATGAATTTCCTTGCACCTTTACGTAGCGGAGACAAGTTTGTAGTGAAA**GCGAGGATATCGGGG**ACATCTGCTGCGCGTATTTACTTCGATCAT**TTCATCTTT**AAACTTCCAAATCAAGAGGTTATATTGGAGGCGAAA**GGAATAGCT**GTATGGCTTGACAACAAGCACCGTCCTGTTCGTATCCCATCTTCGATACGCTCTAAATTTGTTCACTTCCTACGCCAAAACGACACAGTTTGAAAGCTTGCGGCCGCAC

*At*ALT4-ABCDE

AATGGGTCGCGGATCCATGAGTGAGTTCCATGAGGTTGAGCTTAAAGTTCGTGATTATGAATTGGATCAGTTTGGTGTTGTGAACAATGCTGTTTACGCAAACTACTGCCAACACGGC**CGA**CACGAATTTCTAGAGAGTATTGGTATCAACTGTGATGAAGTTGCCCGTTCTGGTGAAGCCTTAGCAATATCAGAGTTGACAATG**AAGTTCCTTTCA**CCTTTACGTAGCGGAGACAAGTTTGTAGTGAAA**GCGAGGATATCGGGG**ACATCTGCTGCGCGTATTTACTTCGATCAT**TTCATCTTT**AAACTTCCAAATCAAGAGGTTATATTGGAGGCGAAA**GGAATAGCT**GTATGGCTTGACAACAAGCACCGTCCTGTTCGTATCCCATCTTCGATACGCTCTAAATTTGTTCACTTCCTACGCCAAAACGACACAGTTTGAAAGCTTGCGGCCGCAC

*At*ALT4-BC

AATGGGTCGCGGATCCATGAGTGAGTTCCATGAGGTTGAGCTTAAAGTTCGTGATTATGAATTGGATCAGTTTGGTGTTGTGAACAATGCTGTTTACGCAAACTACTGCCAACACGGCATGCACGAATTTCTAGAGAGTATTGGTATCAACTGTGATGAAGTTGCCCGTTCTGGTGAAGCCTTAGCAATATCAGAGTTGACAATGAATTTCCTTGCACCTTTACGTAGCGGAGACAAGTTTGTAGTGAAA**GCGAGGATATCGGGG**ACATCTGCTGCGCGTATTTACTTCGATCATTCCATCTTGAAACTTCCAAATCAAGAGGTTATATTGGAGGCGAAA**GGAATAGCT**GTATGGCTTGACAACAAGCACCGTCCTGTTCGTATCCCATCTTCGATACGCTCTAAATTTGTTCACTTCCTACGCCAAAACGACACAGTTTGAAAGCTTGCGGCCGCAC

*At*ALT4-BCD

AATGGGTCGCGGATCCATGAGTGAGTTCCATGAGGTTGAGCTTAAAGTTCGTGATTATGAATTGGATCAGTTTGGTGTTGTGAACAATGCTGTTTACGCAAACTACTGCCAACACGGCATGCACGAATTTCTAGAGAGTATTGGTATCAACTGTGATGAAGTTGCCCGTTCTGGTGAAGCCTTAGCAATATCAGAGTTGACAATGAATTTCCTTGCACCTTTACGTAGCGGAGACAAGTTTGTAGTGAAA**GCGAGGATATCGGGG**ACATCTGCTGCGCGTATTTACTTCGATCAT**TTCATCTTT**AAACTTCCAAATCAAGAGGTTATATTGGAGGCGAAA**GGAATAGCT**GTATGGCTTGACAACAAGCACCGTCCTGTTCGTATCCCATCTTCGATACGCTCTAAATTTGTTCACTTCCTACGCCAAAACGACACAGTTTGAAAGCTTGCGGCCGCAC

*At*ALT4-BCDE

AATGGGTCGCGGATCCATGAGTGAGTTCCATGAGGTTGAGCTTAAAGTTCGTGATTATGAATTGGATCAGTTTGGTGTTGTGAACAATGCTGTTTACGCAAACTACTGCCAACACGGCATGCACGAATTTCTAGAGAGTATTGGTATCAACTGTGATGAAGTTGCCCGTTCTGGTGAAGCCTTAGCAATATCAGAGTTGACAATG**AAGTTCCTTTCA**CCTTTACGTAGCGGAGACAAGTTTGTAGTGAAA**GCGAGGATATCGGGG**ACATCTGCTGCGCGTATTTACTTCGATCAT**TTCATCTTT**AAACTTCCAAATCAAGAGGTTATATTGGAGGCGAAA**GGAATAGCT**GTATGGCTTGACAACAAGCACCGTCCTGTTCGTATCCCATCTTCGATACGCTCTAAATTTGTTCACTTCCTACGCCAAAACGACACAGTTTGAAAGCTTGCGGCCGCAC

*Mt*ALT2

AATGGGTCGCGGATCCATGAGTGGGTTCTGTGATGTTGAACTAAAAGTTCGCGATTATGAATTGGATCAGTTTGGTGTGGTCAACAATTCTGTTTATGCAGGTTATTGCCAACATGGTCGTCATGAATTTTTGGAAAGCATAGGCATTAATTGTGATGCCGTGGCTCGCTGTGGTGATGCATTGGCATTGTCTGAACTATCCTTCAAATTCCTTGCACCTCTAAGAAGTGGAGACAGATTTGTCGTAAAAGTTAGAGTTTCTGGCTCTTCAGCTGCACGCATATACTTCGATCACTTCATCTATAAGCTACCAAACCAAGAGCCTATTTTGGAAGCCAAGGCCACAGCAGTGTGGCTTGACAAAAACTATCGTCCTATTCGAATTCCGGCAGATATTAAGTCTAAATTTGTTAAATTTATTCGCAATGAAGACTCGTGAAAGCTTGCGGCCGCAC

*Mt*ALT1

AATGGGTCGCGGATCCATGTGTGAGTTCTATGACGTGGAACTTAAAGTTCGTGATTATGAGGTTGATCGGTATGGTGTGGTCAACAATGCAGTTTATGCTAATTATTGCCAACATTGTGGTGATGAATTTTTTAAAAGCATTGGTATTAATTTTGCTGATGTGATTCGGAGTGGTGATGCAATGGCACTGTCAAATTTATCCCTCAAATTCCTTGCACCATTAAGAAGTGGAGACAAATTTGTTGTAAGGGTTAGAATTTCCGGCATATCGGCAGCTCGTTTATACCTTGATCAGTTCATCTATAAGCTACCAAATCACAAGCCTGTTTTGGAAGCCAAAACCACCGTAGTGAGGCTTGATAAAAACTATCGTCCTCTTCGAATTTCAGAAGATATGAAGTCTAAAATTTTTAAATGTATTGGTGGAGACGACTCTTAAAAGCTTGCGGCCGCAC

*Mt*ALT2-A

AATGGGTCGCGGATCCATGAGTGGGTTCTGTGATGTTGAACTAAAAGTTCGCGATTATGAATTGGATCAGTTTGGTGTGGTCAACAATTCTGTTTATGCAGGTTATTGCCAACAT**TGTGGTGAT**GAATTTTTGGAAAGCATAGGCATTAATTGTGATGCCGTGGCTCGCTGTGGTGATGCATTGGCATTGTCTGAACTATCCTTCAAATTCCTTGCACCTCTAAGAAGTGGAGACAGATTTGTCGTAAAAGTTAGAGTTTCTGGCTCTTCAGCTGCACGCATATACTTCGATCACTTCATCTATAAGCTACCAAACCAAGAGCCTATTTTGGAAGCCAAGGCCACAGCAGTGTGGCTTGACAAAAACTATCGTCCTATTCGAATTCCGGCAGATATTAAGTCTAAATTTGTTAAATTTATTCGCAATGAAGACTCGTGAAAGCTTGCGGCCGCAC

*Mt*ALT1-A

AATGGGTCGCGGATCCATGTGTGAGTTCTATGACGTGGAACTTAAAGTTCGTGATTATGAGGTTGATCGGTATGGTGTGGTCAACAATGCAGTTTATGCTAATTATTGCCAACAT**GGTCGTCAT**GAATTTTTTAAAAGCATTGGTATTAATTTTGCTGATGTGATTCGGAGTGGTGATGCAATGGCACTGTCAAATTTATCCCTCAAATTCCTTGCACCATTAAGAAGTGGAGACAAATTTGTTGTAAGGGTTAGAATTTCCGGCATATCGGCAGCTCGTTTATACCTTGATCAGTTCATCTATAAGCTACCAAATCACAAGCCTGTTTTGGAAGCCAAAACCACCGTAGTGAGGCTTGATAAAAACTATCGTCCTCTTCGAATTTCAGAAGATATGAAGTCTAAAATTTTTAAATGTATTGGTGGAGACGACTCTTAAAAGCTTGCGGCCGCAC

*Mt*ALT1-B

AATGGGTCGCGGATCCATGTGTGAGTTCTATGACGTGGAACTTAAAGTTCGTGATTATGAGGTTGATCGGTATGGTGTGGTCAACAATGCAGTTTATGCTAATTATTGCCAACATTGTGGTGATGAATTTTTTAAAAGCATTGGTATTAATTTTGCTGATGTGATTCGGAGTGGTGATGCAATGGCACTGTCAAATTTATCCCTCAAATTCCTTGCACCATTAAGAAGTGGAGACAAATTTGTTGTAAGGGTTAGAATTTCCGGCATATCGGCAGCTCGTTTATACCTTGATCAGTTCATCTATAAGCTACCAAATCACAAGCCTGTTTTGGAAGCC**AAGGCCACAGCA**GTGAGGCTTGATAAAAACTATCGTCCTCTTCGAATTTCAGAAGATATGAAGTCTAAAATTTTTAAATGTATTGGTGGAGACGACTCTTAAAAGCTTGCGGCCGCAC

*Mt*ALT1-AB

AATGGGTCGCGGATCCATGTGTGAGTTCTATGACGTGGAACTTAAAGTTCGTGATTATGAGGTTGATCGGTATGGTGTGGTCAACAATGCAGTTTATGCTAATTATTGCCAACAT**GGTCGTCAT**GAATTTTTTAAAAGCATTGGTATTAATTTTGCTGATGTGATTCGGAGTGGTGATGCAATGGCACTGTCAAATTTATCCCTCAAATTCCTTGCACCATTAAGAAGTGGAGACAAATTTGTTGTAAGGGTTAGAATTTCCGGCATATCGGCAGCTCGTTTATACCTTGATCAGTTCATCTATAAGCTACCAAATCACAAGCCTGTTTTGGAAGCC**AAGGCCACAGCA**GTGAGGCTTGATAAAAACTATCGTCCTCTTCGAATTTCAGAAGATATGAAGTCTAAAATTTTTAAATGTATTGGTGGAGACGACTCTTAAAAGCTTGCGGCCGCAC

*Mt*ALT1-C

AATGGGTCGCGGATCCATGTGTGAGTTCTATGACGTGGAACTTAAAGTTCGTGATTATGAGGTTGATCGGTATGGTGTGGTCAACAATGCAGTTTATGCTAATTATTGCCAACATTGTGGTGATGAATTTTTTAAAAGCATTGGTATTAATTTTGCTGATGTGATTCGGAGTGGTGATGCAATGGCACTGTCAAATTTATCCCTCAAATTCCTTGCACCATTAAGAAGTGGAGACAAATTTGTTGTAAGGGTTAGA**GTTTCTGGCTCTTCAGCTGCACGCATATACTTCGATCAC**TTCATCTATAAGCTACCAAATCACAAGCCTGTTTTGGAAGCCAAAACCACCGTAGTGAGGCTTGATAAAAACTATCGTCCTCTTCGAATTTCAGAAGATATGAAGTCTAAAATTTTTAAATGTATTGGTGGAGACGACTCTTAAAAGCTTGCGGCCGCAC

*Mt*ALT1-ABC

AATGGGTCGCGGATCCATGTGTGAGTTCTATGACGTGGAACTTAAAGTTCGTGATTATGAGGTTGATCGGTATGGTGTGGTCAACAATGCAGTTTATGCTAATTATTGCCAACAT**GGTCGTCAT**GAATTTTTTAAAAGCATTGGTATTAATTTTGCTGATGTGATTCGGAGTGGTGATGCAATGGCACTGTCAAATTTATCCCTCAAATTCCTTGCACCATTAAGAAGTGGAGACAAATTTGTTGTAAGGGTTAGA**GTTTCTGGCTCTTCAGCTGCACGCATATACTTCGATCAC**TTCATCTATAAGCTACCAAATCACAAGCCTGTTTTGGAAGCC**AAGGCCACAGCA**GTGAGGCTTGATAAAAACTATCGTCCTCTTCGAATTTCAGAAGATATGAAGTCTAAAATTTTTAAATGTATTGGTGGAGACGACTCTTAAAAGCTTGCGGCCGCAC

*Mt*ALT1-ABC2

AATGGGTCGCGGATCCATGTGTGAGTTCTATGACGTGGAACTTAAAGTTCGTGATTATGAGGTTGATCGGTATGGTGTGGTCAACAATGCAGTTTATGCTAATTATTGCCAACAT**GGTCGTCAT**GAATTTTTTAAAAGCATTGGTATTAATTTTGCTGATGTGATTCGGAGTGGTGATGCAATGGCACTGTCAAATTTATCCCTCAAATTCCTTGCACCATTAAGAAGTGGAGACAAATTTGTTGTAAGGGTTAGA**GTTTCTGGCTCTTCAGCTGCACGCATA**TACCTTGATCAGTTCATCTATAAGCTACCAAATCACAAGCCTGTTTTGGAAGCC**AAGGCCACAGCA**GTGAGGCTTGATAAAAACTATCGTCCTCTTCGAATTTCAGAAGATATGAAGTCTAAAATTTTTAAATGTATTGGTGGAGACGACTCTTAAAAGCTTGCGGCCGCAC

*Mt*ALT1-ABC + aa78-82

AATGGGTCGCGGATCCATGTGTGAGTTCTATGACGTGGAACTTAAAGTTCGTGATTATGAGGTTGATCGGTATGGTGTGGTCAACAATGCAGTTTATGCTAATTATTGCCAACAT**GGTCGTCAT**GAATTTTTTAAAAGCATTGGTATTAATTTTGCTGATGTGATTCGGAGTGGTGATGCAATGGCACTGTCAAATTTATCCCTCAAATTCCTTGCACCATTAAGAAGTGGAGACAAATTTGTTGTAAGGGTTAGA**GTTTCTGGCTCTTCA**GCAGCTCGTTTATACCTTGATCAGTTCATCTATAAGCTACCAAATCACAAGCCTGTTTTGGAAGCC**AAGGCCACAGCA**GTGAGGCTTGATAAAAACTATCGTCCTCTTCGAATTTCAGAAGATATGAAGTCTAAAATTTTTAAATGTATTGGTGGAGACGACTCTTAAAAGCTTGCGGCCGCAC

*Zm*ALT1

AATGGGTCGCGGATCCAAGGACAAGTTTTTCGAGATCGAGATGGAGGTGCGCGACGACGAGCTTGACGAGTACGGCGTCGTCAACAACGCCATCTACGCCAGCTACCTCCATAGCGGTCGTGACGTGGTGCTTGAGAAGCTGGGCATCAGCGTGGACTACTGGACATCCACGGGCAACGCCATGGCTCTTTCAGAGCTCAACCTCAAGTATTTCGCGCCTTTGAGGAGCGGCGACAGGTTCGTCGTGAAGGTGAAGCCTGTCCAAATCAAAGGCGTGCGGATGATTGTGGAGCACATGATCGAGGCCCTGCCGGATCGTAAGCTCGTCATGGAAGGCAGAGCGACCGTCGTTTGCCTCAACAAGGACTTCCGTCCAACTCGGGTATTCCCGGAGTTAGCAGCAAGAGCCAAGGAAGTTTTCTCCTGCAAGGTTGCATAAAAGCTTGCGGCCGCAC

*Zm*ALT3

AATGGGTCGCGGATCCGCCGGCAAGTTTTTCGAGTTGGAGATGACGGTCCGTGACTGCGACCTCGACGTGTACGGGGTCGTCAACAATGCTGTGTATGCTGGGTACATCGAAATAGCTCGTCAAGAGATGCTTGCAAGCCTCGGCGTCTGCACGGCCTCGATCGTGCGCACGGACCGTGCCATGGCGCTCTCCGAGCTGAACGTCAAGTACTTCGCGCCACTCAAGCGCGGCGCCAAGTTCGTCGTCATGGTGAGGGTTGTGCAAATCAAGGGTGTGCGAATGCTCATGGAGCACTTGATCGCGACGCTGCCGGACCGCAAGCTCGTGCTAGAAGCGACGGCCACTGTCGTCTGCCTCAACCAAGAGTACCGCCCAACTCGCATGTTCCCGGAGATGGCCAAGCTGCTGCCCTTCTTCTCTCACCCTAATTAGAAGCTTGCGGCCGCAC

*Zm*ALT1-A

AATGGGTCGCGGATCCAAGGACAAGTTTTTCGAGATCGAGATGGAGGTGCGCGACGACGAGCTTGACGAGTACGGCGTCGTCAACAACGCCATCTACGCCAGCTAC**ATCGAAATAGCTCGTCAA**GTGGTGCTTGAGAAGCTGGGCATCAGCGTGGACTACTGGACATCCACGGGCAACGCCATGGCTCTTTCAGAGCTCAACCTCAAGTATTTCGCGCCTTTGAGGAGCGGCGACAGGTTCGTCGTGAAGGTGAAGCCTGTCCAAATCAAAGGCGTGCGGATGATTGTGGAGCACATGATCGAGGCCCTGCCGGATCGTAAGCTCGTCATGGAAGGCAGAGCGACCGTCGTTTGCCTCAACAAGGACTTCCGTCCAACTCGGGTATTCCCGGAGTTAGCAGCAAGAGCCAAGGAAGTTTTCTCCTGCAAGGTTGCATAAAAGCTTGCGGCCGCAC

ZmALT3-A

AATGGGTCGCGGATCCGCCGGCAAGTTTTTCGAGTTGGAGATGACGGTCCGTGACTGCGACCTCGACGTGTACGGGGTCGTCAACAATGCTGTGTATGCTGGGTAC**CTCCATAGCGGTCGTGAC**GAGATGCTTGCAAGCCTCGGCGTCTGCACGGCCTCGATCGTGCGCACGGACCGTGCCATGGCGCTCTCCGAGCTGAACGTCAAGTACTTCGCGCCACTCAAGCGCGGCGCCAAGTTCGTCGTCATGGTGAGGGTTGTGCAAATCAAGGGTGTGCGAATGCTCATGGAGCACTTGATCGCGACGCTGCCGGACCGCAAGCTCGTGCTAGAAGCGACGGCCACTGTCGTCTGCCTCAACCAAGAGTACCGCCCAACTCGCATGTTCCCGGAGATGGCCAAGCTGCTGCCCTTCTTCTCTCACCCTAATTAGAAGCTTGCGGCCGCAC

*Zm*ALT1-B

AATGGGTCGCGGATCCAAGGACAAGTTTTTCGAGATCGAGATGGAGGTGCGCGACGACGAGCTTGACGAGTACGGCGTCGTCAACAACGCCATCTACGCCAGCTACCTCCATAGCGGTCGTGACGTGGTGCTTGAGAAGCTGGGCATCAGCGTGGACTACTGGACATCCACGGGCAACGCCATGGCTCTTTCAGAGCTCAACCTCAAGTATTTCGCGCCTTTGAGGAGCGGCGACAGGTTCGTCGTGAAGGTGAAGCCTGTCCAAATCAAAGGCGTGCGGATGATTGTGGAGCACATGATCGAGGCCCTGCCGGATCGTAAGCTCGTCATGGAAGGC**ACG**GCGACCGTCGTTTGCCTCAACAAGGACTTCCGTCCAACTCGGGTATTCCCGGAGTTAGCAGCAAGAGCCAAGGAAGTTTTCTCCTGCAAGGTTGCATAAAAGCTTGCGGCCGCAC

*Zm*ALT3-B

AATGGGTCGCGGATCCGCCGGCAAGTTTTTCGAGTTGGAGATGACGGTCCGTGACTGCGACCTCGACGTGTACGGGGTCGTCAACAATGCTGTGTATGCTGGGTACATCGAAATAGCTCGTCAAGAGATGCTTGCAAGCCTCGGCGTCTGCACGGCCTCGATCGTGCGCACGGACCGTGCCATGGCGCTCTCCGAGCTGAACGTCAAGTACTTCGCGCCACTCAAGCGCGGCGCCAAGTTCGTCGTCATGGTGAGGGTTGTGCAAATCAAGGGTGTGCGAATGCTCATGGAGCACTTGATCGCGACGCTGCCGGACCGCAAGCTCGTGCTAGAAGCG**AGA**GCCACTGTCGTCTGCCTCAACCAAGAGTACCGCCCAACTCGCATGTTCCCGGAGATGGCCAAGCTGCTGCCCTTCTTCTCTCACCCTAATTAGAAGCTTGCGGCCGCAC

ZmALT1-C

AATGGGTCGCGGATCCAAGGACAAGTTTTTCGAGATCGAGATGGAGGTGCGCGACGACGAGCTTGACGAGTACGGCGTCGTCAACAACGCCATCTACGCCAGCTACCTCCATAGCGGTCGTGACGTGGTGCTTGAGAAGCTGGGCATCAGCGTGGACTACTGGACATCCACGGGCAACGCCATGGCTCTTTCAGAGCTCAACCTCAAGTATTTCGCGCCTTTGAGGAGCGGCGACAGGTTCGTCGTG**ATGGTGAGGGTTGTGCAAATCAAGGGTGTGCGAATGCTCATG**GAGCACATGATCGAGGCCCTGCCGGATCGTAAGCTCGTCATGGAAGGCAGAGCGACCGTCGTTTGCCTCAACAAGGACTTCCGTCCAACTCGGGTATTCCCGGAGTTAGCAGCAAGAGCCAAGGAAGTTTTCTCCTGCAAGGTTGCATAAAAGCTTGCGGCCGCAC

ZmALT3-C

AATGGGTCGCGGATCCGCCGGCAAGTTTTTCGAGTTGGAGATGACGGTCCGTGACTGCGACCTCGACGTGTACGGGGTCGTCAACAATGCTGTGTATGCTGGGTACATCGAAATAGCTCGTCAAGAGATGCTTGCAAGCCTCGGCGTCTGCACGGCCTCGATCGTGCGCACGGACCGTGCCATGGCGCTCTCCGAGCTGAACGTCAAGTACTTCGCGCCACTCAAGCGCGGCGCCAAGTTCGTCGTC**AAGGTGAAGCCTGTCCAAATCAAAGGCGTGCGGATGATTGTG**GAGCACTTGATCGCGACGCTGCCGGACCGCAAGCTCGTGCTAGAAGCGACGGCCACTGTCGTCTGCCTCAACCAAGAGTACCGCCCAACTCGCATGTTCCCGGAGATGGCCAAGCTGCTGCCCTTCTTCTCTCACCCTAATTAGAAGCTTGCGGCCGCAC
