# Supplementary material for: Determinants of substrate specificity in a catalytically diverse family of acyl-ACP thioesterases from plants
Source: BMC Plant Biol. 2023 Jan 2;23:1. doi: 10.1186/s12870-022-04003-y (PMC9806908; doi:10.1186/s12870-022-04003-y)
Supplement: Supplementary file 6 — Additional file 6: Fig. S3. EI mass spectra of compounds detected in media from K27(DE3) E. coli cultures expressing ALTs. β-keto fatty acids secreted into culture media were chemically decarboxylated to methylketones prior to GC-MS analysis. Standard spectra used for compound identification are shown to the right of sample spectra. Fully reduced free fatty acids and saturated methylketones were identified by comparison to standard spectra from the NIST 17 Mass Spectral Library. Monounsaturated methylketones were identified by comparison to spectra previously obtained by Goh et al. [30]. 3-hydroxy fatty acids were identified based on the presence of their trimethylsilyl (TMS) derivatives in silylated samples. Mass spectra of the 2TMS derivatives of 3-hydroxy fatty acids were compared to standard spectra in the NIST 17 library, with the exception of 7(Z)-3-hydroxyhexadec-7-enoic acid, which is unlisted. The 2TMS derivative of 7(Z)-3-hydroxyhexadec-7-enoic acid was identified based the presence of the molecular ion (m/z = 386) and prominent ion fragments characteristic of both the 2TMS derivative of a 3-hydroxy fatty acid (m/z = 73, m/z = 147, m/z = 233, M+ − 31) and the TMS derivative of a monounsaturated fatty acid (M+ − 169, M+ − 90). Indole, a quorum sensing molecule, was present in media from all induced cultures. [file 12870_2022_4003_MOESM6_ESM.docx]

**Additional file 6: Fig. S3.** EI mass spectra of compounds detected in media from K27(DE3) *E. coli* cultures expressing ALTs. β-keto fatty acids secreted into culture media were chemically decarboxylated to methylketones prior to GC-MS analysis. Standard spectra used for compound identification are shown to the right of sample spectra. Fully reduced free fatty acids and saturated methylketones were identified by comparison to standard spectra from the NIST 17 Mass Spectral Library. Monounsaturated methylketones were identified by comparison to spectra previously obtained by Goh *et al.* [30]. 3-hydroxy fatty acids were identified based on the presence of their trimethylsilyl (TMS) derivatives in silylated samples. Mass spectra of the 2TMS derivatives of 3-hydroxy fatty acids were compared to standard spectra in the NIST 17 library, with the exception of 7(Z)-3-hydroxyhexadec-7-enoic acid, which is unlisted. The 2TMS derivative of 7(Z)-3-hydroxyhexadec-7-enoic acid was identified based the presence of the molecular ion (m/z = 386) and prominent ion fragments characteristic of both the 2TMS derivative of a 3-hydroxy fatty acid (m/z = 73, m/z = 147, m/z = 233, M^+^ – 31) and the TMS derivative of a monounsaturated fatty acid (M^+^ – 169, M^+^ – 90). Indole, a quorum sensing molecule, was present in media from all induced cultures.


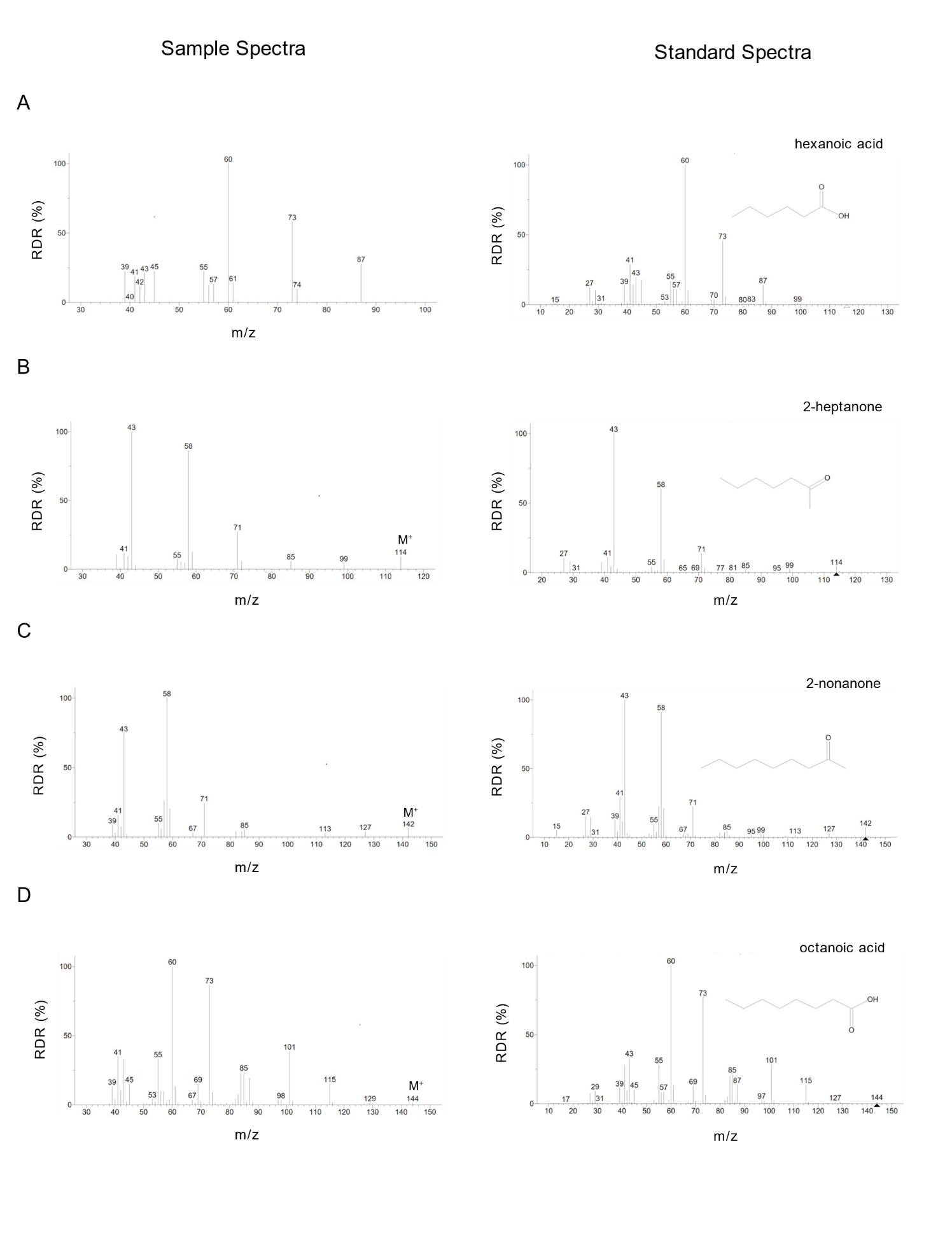


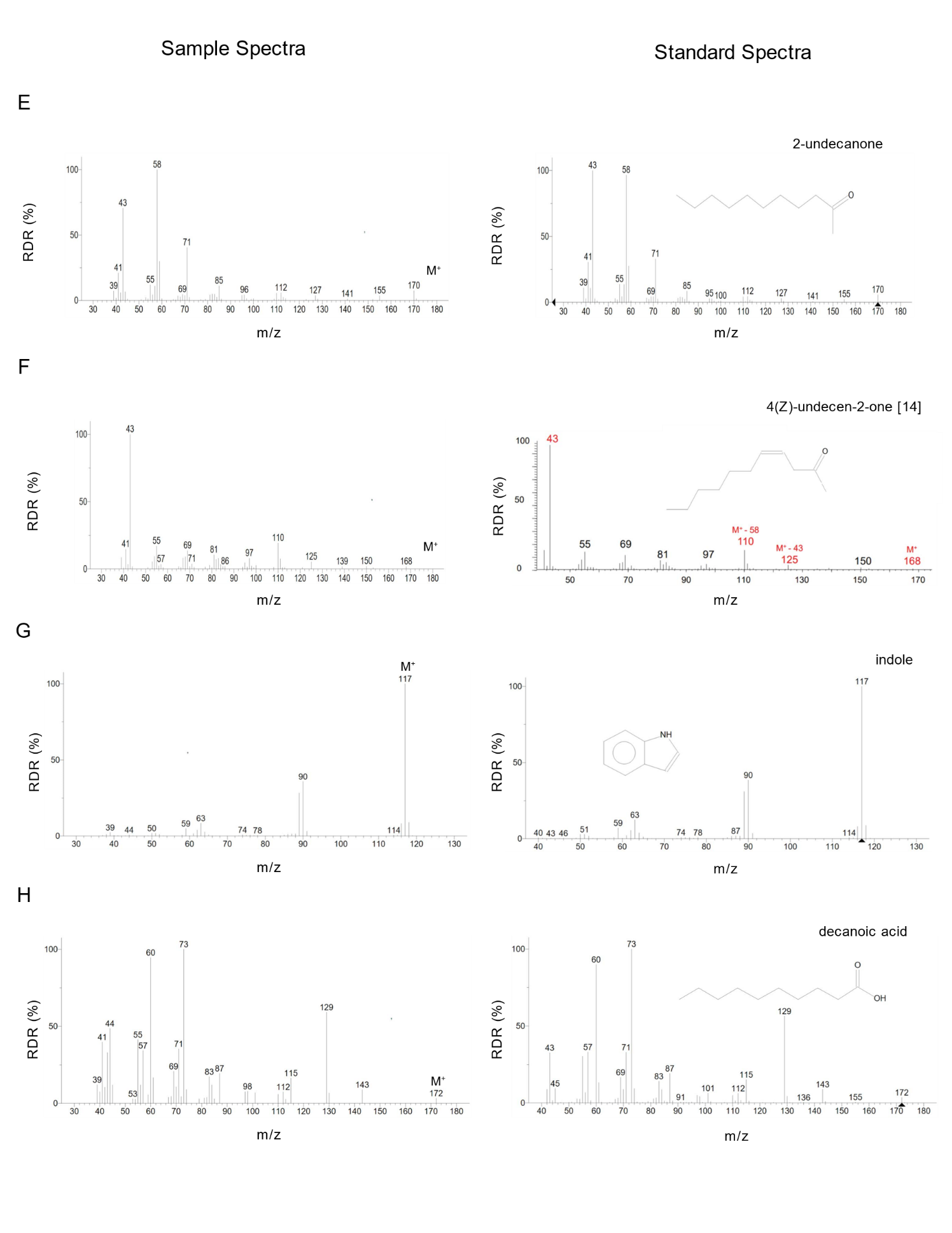

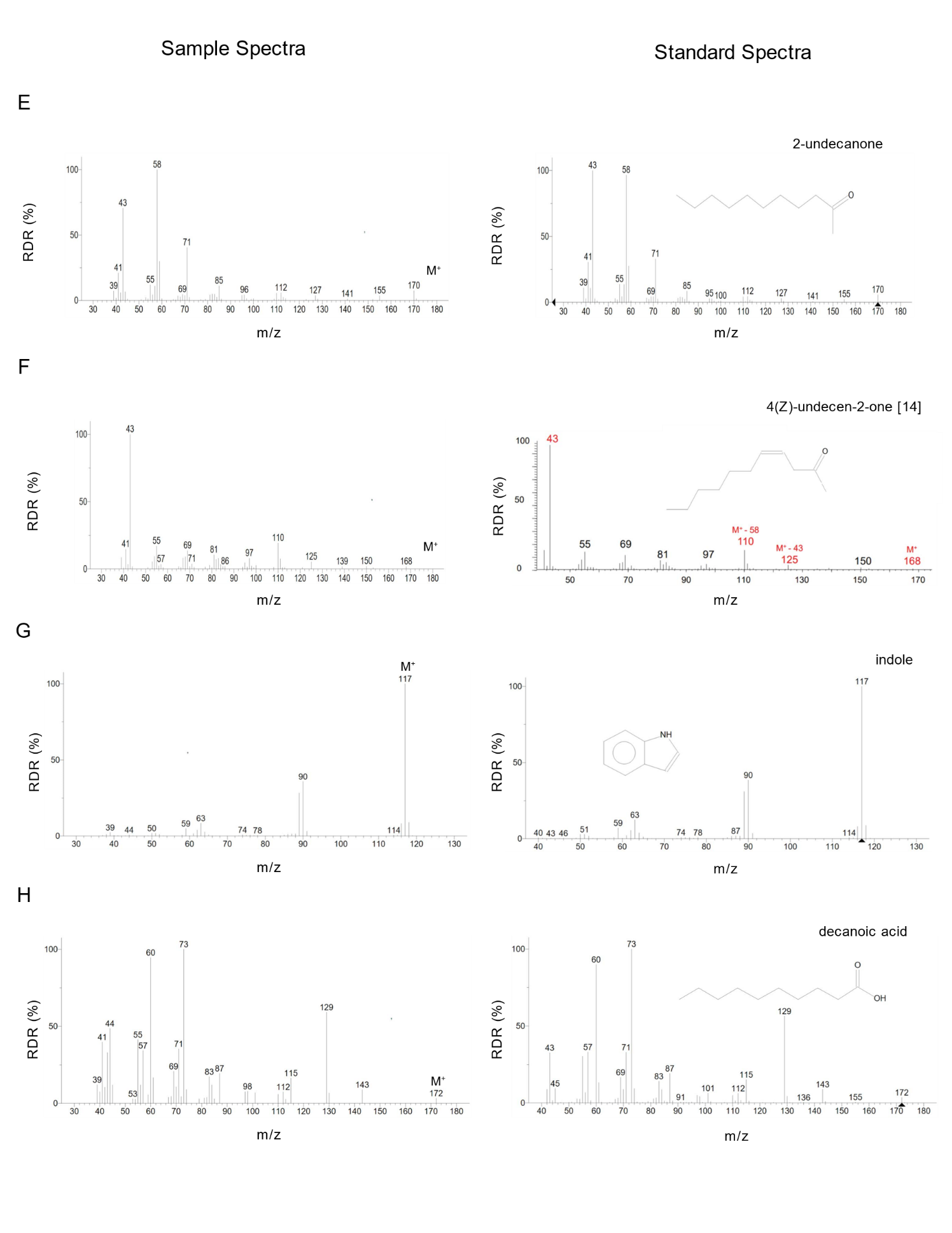

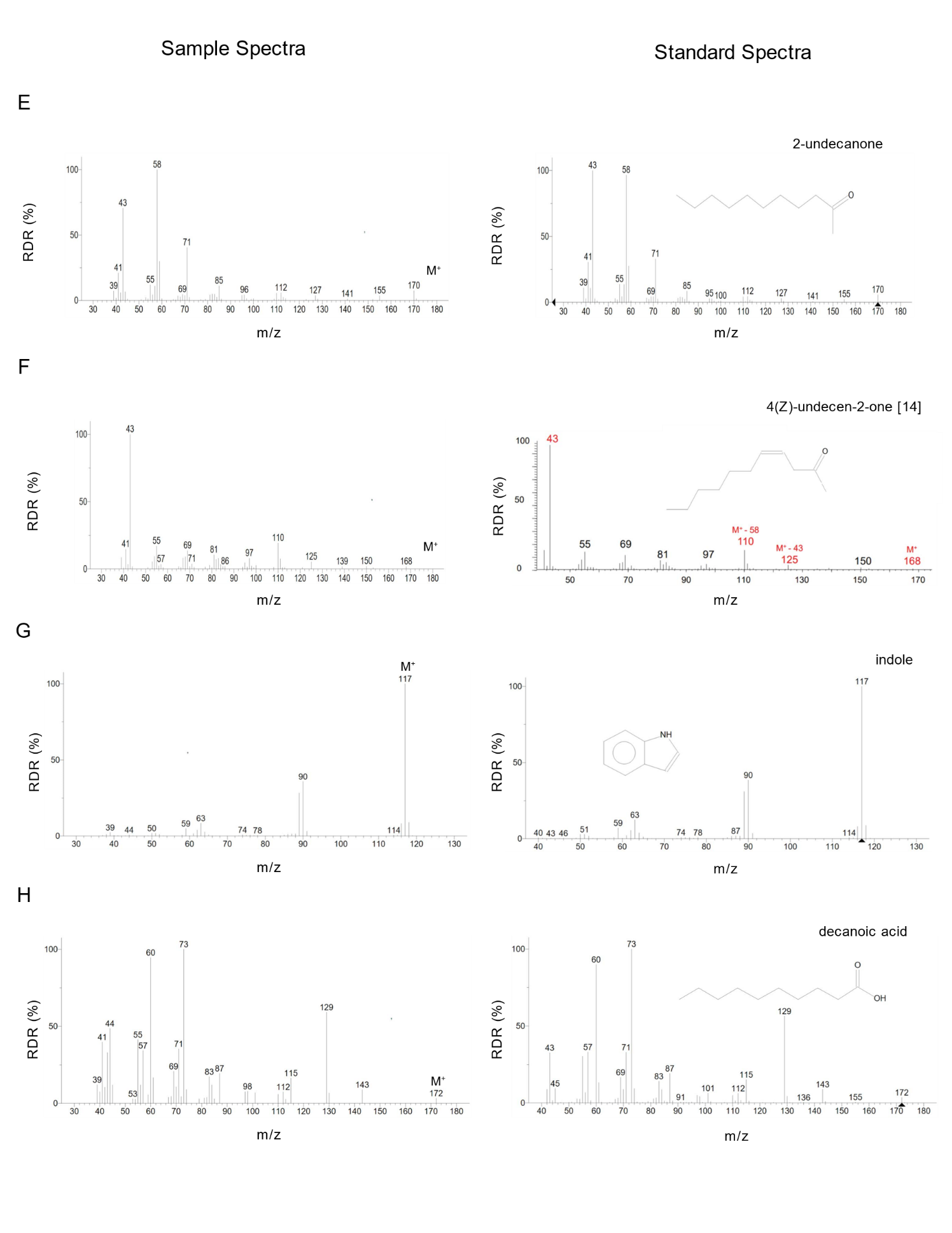


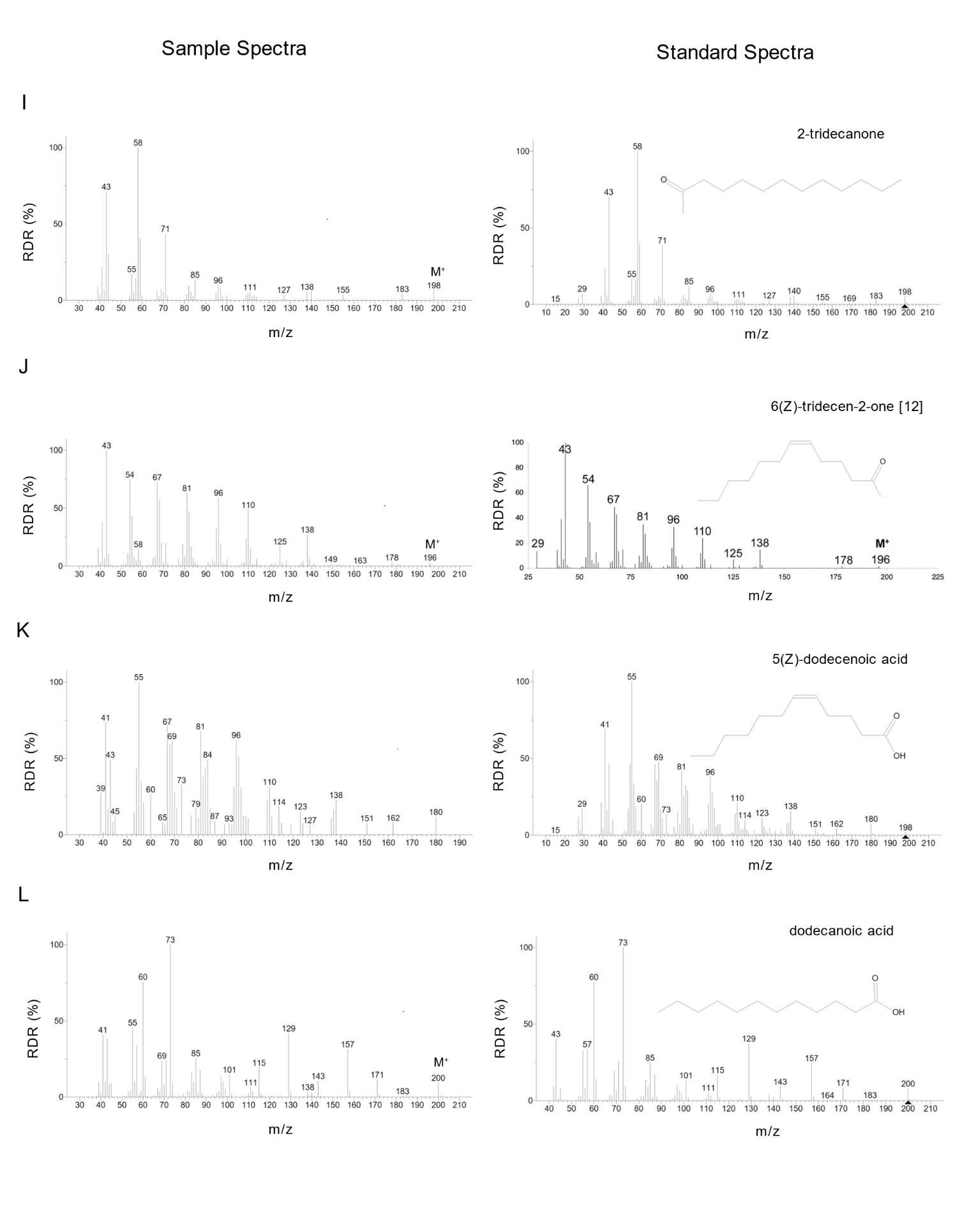


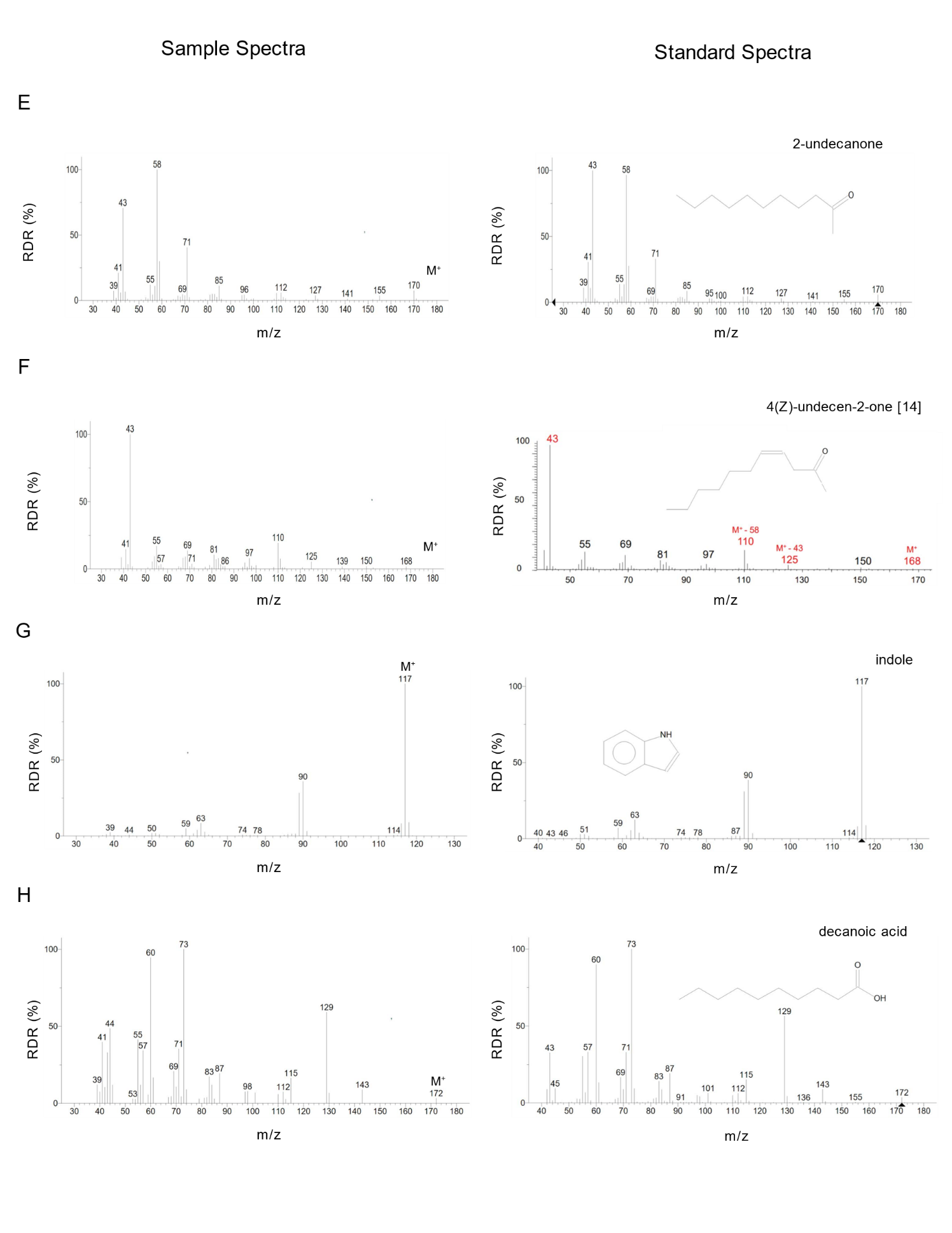
**
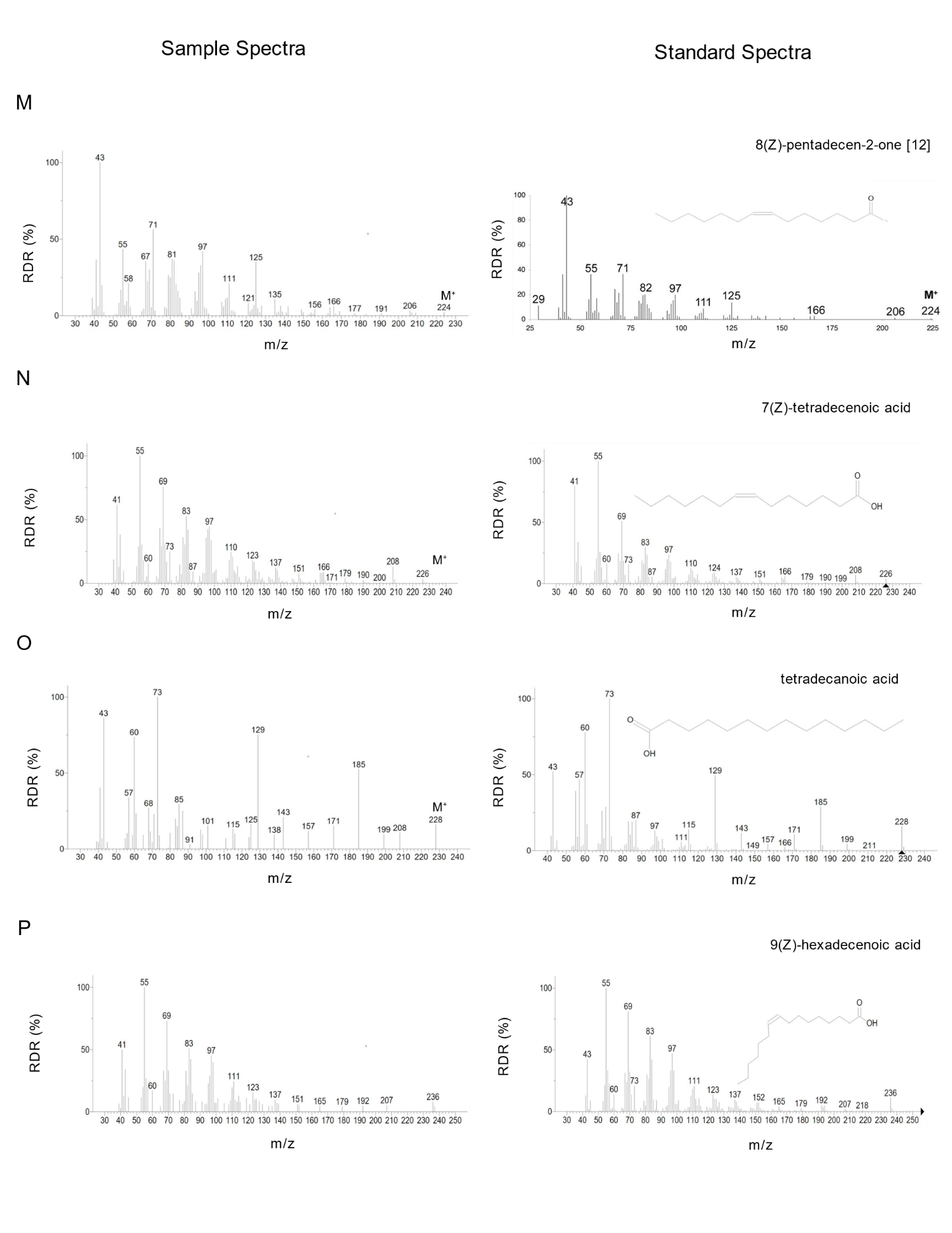
**

**
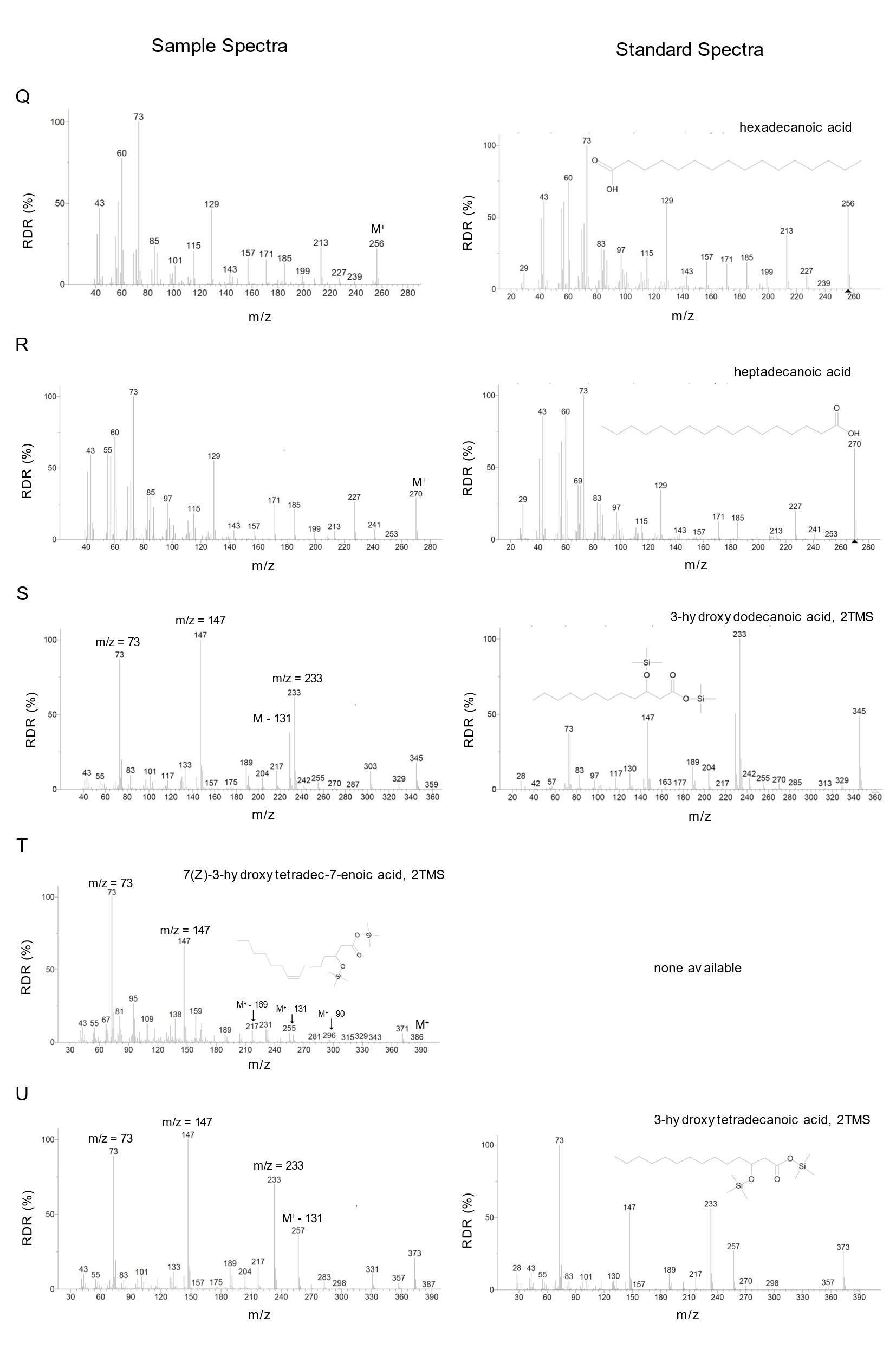
**

**
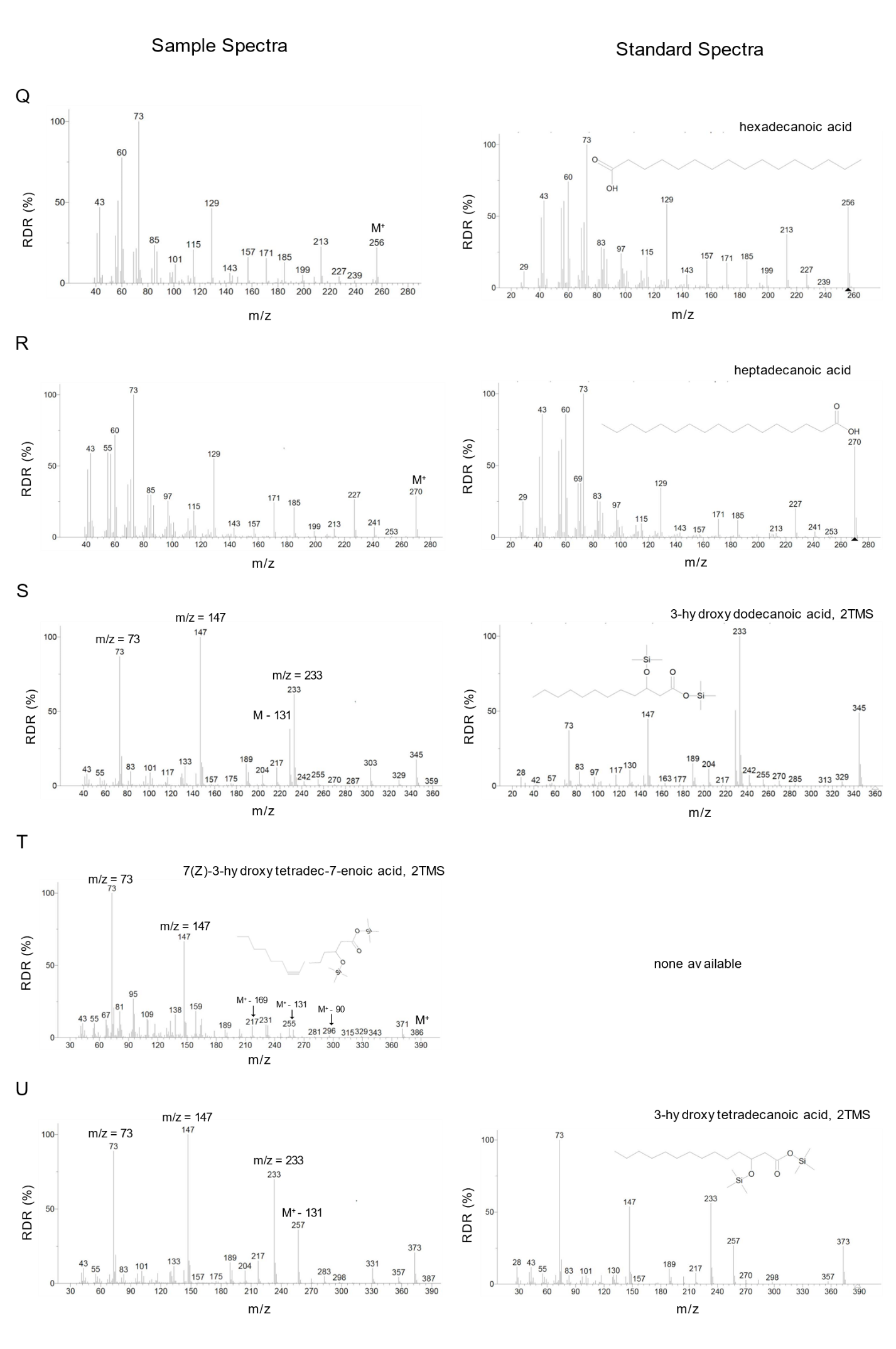
**

**
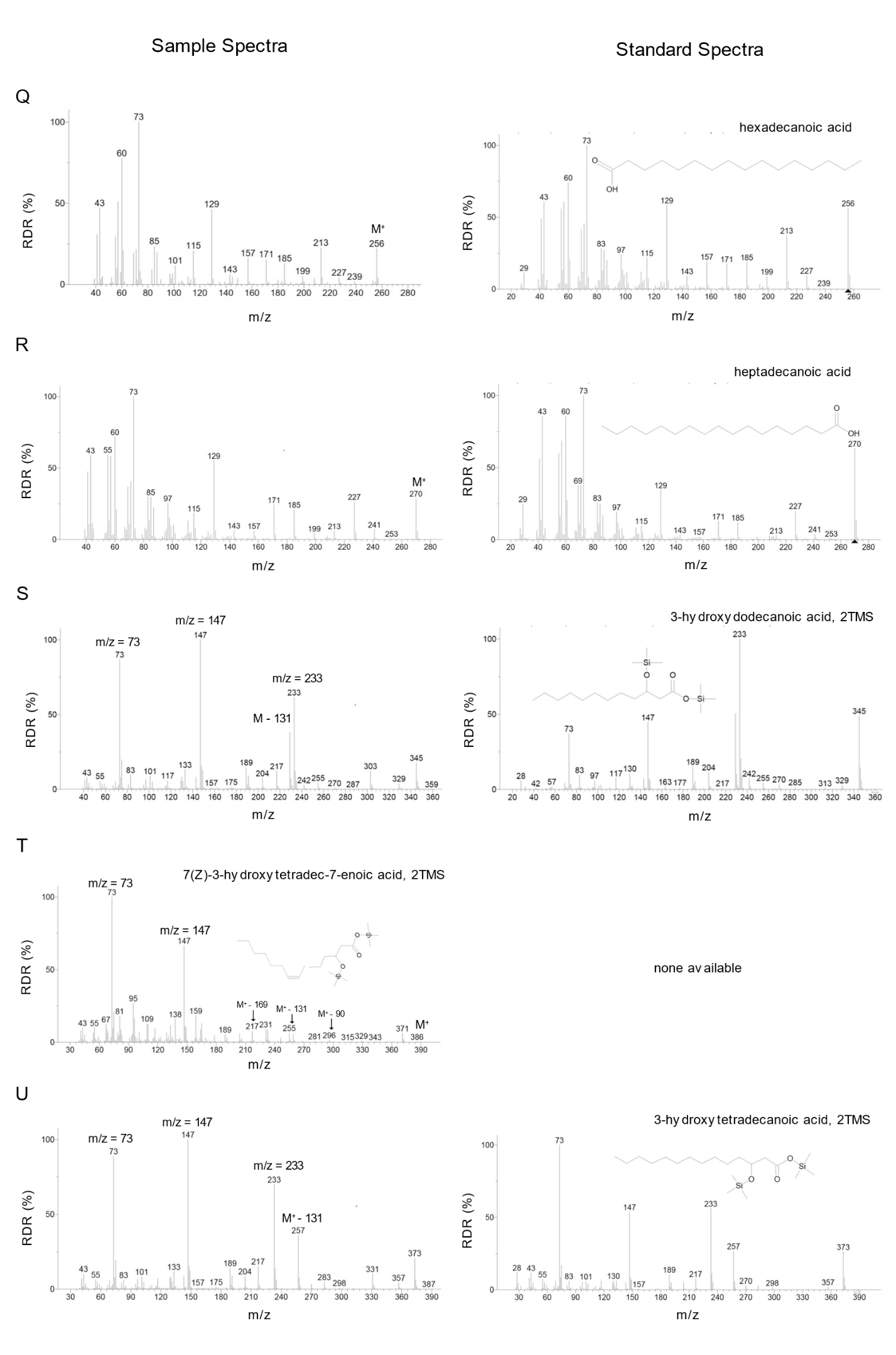
**
